# Complete sequences of *Schizosaccharomyces pombe* subtelomeres reveal multiple patterns of genome variation

**DOI:** 10.1038/s41467-020-20595-1

**Published:** 2021-01-27

**Authors:** Yusuke Oizumi, Takuto Kaji, Sanki Tashiro, Yumiko Takeshita, Yuko Date, Junko Kanoh

**Affiliations:** 1 Department of Life Sciences, Graduate School of Arts and Sciences, The University of Tokyo, 3-8-1 Komaba, Meguro-ku, Tokyo, 153-8902 Japan; 2 Institute for Protein Research, Osaka University, 3-2 Yamadaoka, Suita, Osaka, 565-0871 Japan; 3 Department of Biological Sciences, Graduate School of Science, The University of Tokyo, 7-3-1 Hongo, Bunkyo-ku, Tokyo, 113-0033 Japan; 4 Present Address: Institute of Molecular Biology, University of Oregon, 1370 Franklin Blvd, Eugene, OR USA

**Keywords:** Evolutionary biology, Structural variation, DNA sequencing

## Abstract

Genome sequences have been determined for many model organisms; however, repetitive regions such as centromeres, telomeres, and subtelomeres have not yet been sequenced completely. Here, we report the complete sequences of subtelomeric homologous (*SH*) regions of the fission yeast *Schizosaccharomyces pombe*. We overcame technical difficulties to obtain subtelomeric repetitive sequences by constructing strains that possess single *SH* regions of a standard laboratory strain. In addition, some natural isolates of *S. pombe* were analyzed using previous sequencing data. Whole sequences of *SH* regions revealed that each *SH* region consists of two distinct parts with mosaics of multiple common segments or blocks showing high variation among subtelomeres and strains. Subtelomere regions show relatively high frequency of nucleotide variations among strains compared with the other chromosomal regions. Furthermore, we identified subtelomeric RecQ-type helicase genes, *tlh3* and *tlh4*, which add to the already known *tlh1* and *tlh2*, and found that the *tlh1–4* genes show high sequence variation with missense mutations, insertions, and deletions but no severe effects on their RNA expression. Our results indicate that *SH* sequences are highly polymorphic and hot spots for genome variation. These features of subtelomeres may have contributed to genome diversity and, conversely, various diseases.

## Abstract (web summary)

Sequencing and mapping of long repetitive regions can be challenging due to technical difficulties in sequencing and assembly of the sequence data. Here authors report the complete sequences of subtelomeric homologous (SH) regions of the fission yeast Schizosaccharomyces pombe to reveal highly polymorphic and hot spots for genome variation features.

## Introduction

Genomic DNA sequences provide fundamental information for biological study. The genomes of model organisms, such as *Saccharomyces cerevisiae* (*S. cerevisiae*)^1^, *Schizosaccharomyces pombe* (*S. pombe*)^2^, *Caenorhabditis elegans*^3^, *Drosophila melanogaster*^4^, *Arabidopsis thaliana*^5^, and *Homo sapiens*^6–8^, have been sequenced in the past two decades, and most parts of these sequences have been reported. However, sequencing of long repetitive regions has not been completed because of technical difficulties in sequencing and chromosome allocation of such regions, as well as frequent occurrence of mutations and structural changes caused by chromosome rearrangements, such as recombination, translocation, chromosome breakage, and fusion^9–13^.

Incomplete genomic DNA information can lead to inaccurate data in some experiments. For instance, we are unable to determine the precise chromatin localization of proteins in repetitive regions without actual DNA sequences. Evaluation of protein localization by chromatin immunoprecipitation assays involves PCR with sets of representative primers that target repetitive sequences or Southern blot analysis with representative probes. Chromatin localization values obtained using representative primers or probes merely show averages of all regions that have the target sequences, and they do not reflect actual patterns of chromatin association. Next-generation sequencers (NGSs) do not solve this problem if complete genome sequences are not provided. In addition, there may be uncharacterized genes in un-sequenced regions. Therefore, complete sequences of genomic DNA are crucial for accurate analyses and a deeper understanding of model organisms.

Telomeres, which exist at chromosome ends and possess species-specific tandem repeat sequences, play crucial roles in several cellular activities required for cell survival, including protection of chromosome ends, length regulation of telomere-specific repeat DNA, and regulation of chromosome movements during mitosis and meiosis^14–17^. Subtelomeres, which are adjacent to telomeres, have sequences distinct from telomere repeats and generally contain multiple species-specific segments that share high similarity with other subtelomeres. In the budding yeast *S. cerevisiae*, the subtelomeres contain X and Y′ elements, the latter of which includes the open reading frame (ORF) of a helicase gene^18^. In humans, the subtelomeres are mosaics of ~50 types of common segments containing various ORFs, such as those for the *DUX4* gene, which is related to facioscapulohumeral muscular dystrophy, and for the olfactory receptor family genes^9,19,20^. Although substantial knowledge of telomeres has accumulated, research on subtelomeres has progressed slowly compared with research on other chromosomal regions because of technical difficulties caused by long and repetitive nature of this region and partially unknown sequences.

**Fig. 1:**
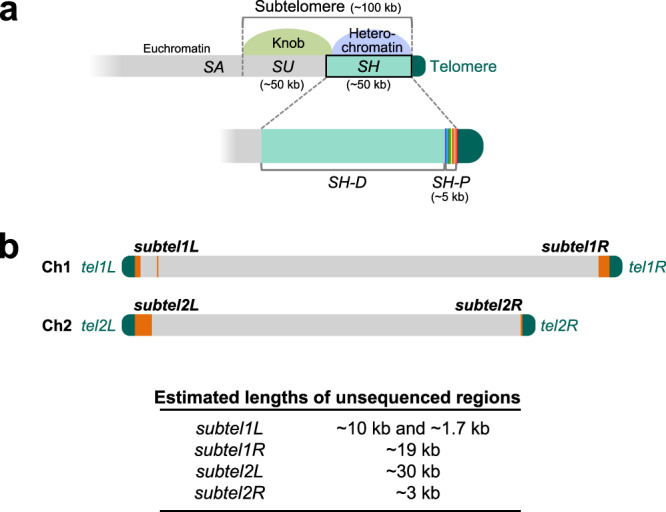
Structures and previously unsequenced regions of subtelomeres in *S. pombe 972* strain. **a** Schematic illustration of the structures of subtelomeres (~100 kb) of Ch1 and Ch2 in strain *972*. The *SH* region (~50 kb) shows high sequence identity (>90%) with other subtelomeres. Subtelomeric heterochromatin is formed around the *SH* region^21^. An *SH* sequence is composed of two characteristic regions, *SH-P* (~5 kb) and *SH-D*. In contrast, the *SH*-adjacent *SU* region (~50 kb) shows low sequence identities with other subtelomeres, but forms a highly condensed knob structure that is shared among them^24^. *SA* indicates a subtelomere-adjacent euchromatin region. **b** Schematic illustration of unsequenced regions of subtelomeres in Ch1 and Ch2 of strain *972* according to PomBase (indicated by orange boxes). *Tel1L*, *tel1R*, *tel2L*, and *tel2R* indicate telomeres at the left and right arms of Ch1 and those of Ch2, respectively. *Subtel1L*, *subtel1R*, *subtel2L*, and *subtel2R* indicate subtelomeres at the left and right arms of Ch1 and those of Ch2, respectively. Lengths of unsequenced regions are estimated based on the assumption that these *SH* sequences show high similarity with that of *subtel2R* of PomBase. Note that Ch3 is omitted in this panel (see Fig. 2a for the ends of Ch3 in strain *972*).

The fission yeast *S. pombe* is one of the most commonly used yeast model organisms for biological study. It preferentially proliferates as haploid in nutrient-rich media and possesses only three chromosomes (chromosome 1 [Ch1], 5.6 Mb; Ch2, 4.6 Mb; Ch3, 3.5 Mb), which enables the whole package of genetic analyses, such as screening for both dominant and recessive mutations, and generation of cells with circular chromosomes by deleting telomere DNA. *S. pombe* subtelomeres spanning ~100 kb are subdivided into two regions of ~50 kb each, the telomere-adjacent and telomere-distal regions (Fig. 1a). The telomere-adjacent regions (*SH* [subtelomeric homologous] regions) of subtelomeres contain *SH* sequences, which share high similarity (>90% identity) with at least one other subtelomere in *S. pombe*^21^ and form heterochromatin structures^22,23^. This *SH* region is subdivided into two regions, the telomere-proximal *SH-P* and telomere-distal *SH-D* regions by their different features in *972* strains (Fig. 1a; see below). In contrast, the *SH-*adjacent regions (*SU* [subtelomeric unique] regions) share almost no sequence similarity with other subtelomeres, but exhibit common highly condensed chromatin structures called knobs^24^ (Fig. 1a). Because of high sequence similarity, it is very difficult to distinguish individual *SH* regions of subtelomeres at different chromosome ends and to assemble repetitive subtelomeric sequences accurately, even if we use latest NGSs. Therefore, parts of *SH* regions remain un-sequenced for 19 years after vast majority of the *S. pombe* genome sequence was reported (*S. pombe* genome database, PomBase: http://www.PomBase.org/status/sequencing-status)^2^ (Fig. 1b). Previously, parts of the four *SH* regions have been cloned and sequenced (pNSU series, see below)^25^; however, they have not yet been allocated to specific subtelomeres (see PomBase).

## Results

### Construction of strains containing single *SH* regions of *972*

**Fig. 2:**
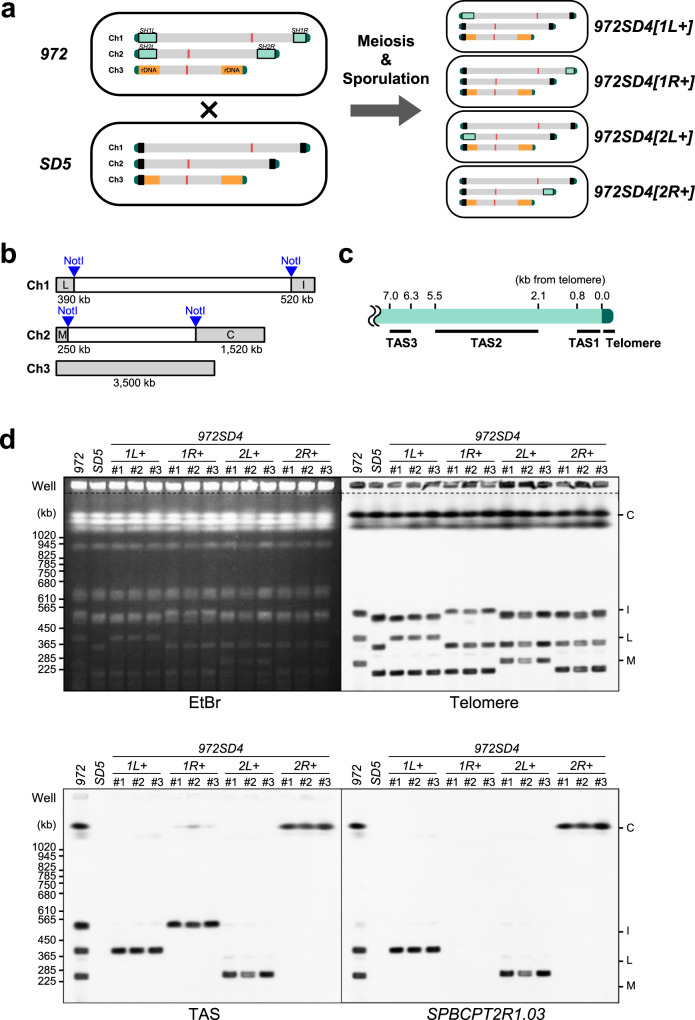
Construction of *972SD4* strains containing a single *SH* region of strain *972*. **a** Construction strategy of *972SD4* strains. Pale green boxes, *SH* regions adjacent to telomeres; black boxes, marker genes (*his7*^+^ or *ura4*^+^) that replaced *SH* regions^21^; orange boxes, rDNA repeats; and red boxes, centromeres. Strains *972* and *SD5* were crossed, and meiosis and sporulation (spore formation) were induced. Progeny with single *SH* regions were obtained. **b** Schematic illustration of telomere-containing NotI restriction fragments (shown in gray). Fragments L, I, M, and C contain *SH1L*, *SH1R*, *SH2L*, and *SH2R*, respectively. The size of each fragment is shown underneath. **c** Schematic illustration of the positions of DNA fragments detected by probes for telomeres and TAS (TAS1–3). Shown are the distances from telomeres in pNSU70. **d** Analyses of the chromosome end structures in *972SD4* strains. NotI-digested chromosomal DNAs of three independent strains of each *972SD4* were analyzed by PFGE followed by Southern blotting using telomere, TAS, or *SPBCPT2R1.03* ORF probes. EtBr, ethidium bromide staining of the gel after PFGE. Note that *SH1R* lacks part of the *SH* region homologous to *SPBCPT2R1.03* according to PomBase. Consistently, *972SD4[1R*+*]* strains did not show an *SPBCPT2R1.03*. signal (also see Fig. 5). These data were reproduced twice.

To overcome the difficulty in allocating each *SH* sequence to a specific subtelomere, we constructed strains containing single *SH* regions of the standard *S. pombe* strain *972* (*h*^−^). Strain *972* used in this study (a derivative of the original *972* (ref. ^26^)), which has not been crossed with other strains, possesses four *SH* sequences (*SH1L*, *SH1R*, *SH2L*, and *SH2R*, as shown in Fig. 2a) adjacent to the telomeres of Ch1 and Ch2, but no *SH* sequence in Ch3 (note that some descendent strains of *972* possess a partial (~16 kb-long) *SH* sequence adjacent to the telomeres of the left and/or right arms of Ch3; see Supplementary Fig. 1)^21,27,28^. We previously produced the *SD5* (*SH* deletion 5) strain, in which all five *SH* regions that are located at both ends of Ch1 and Ch2, and the left end of Ch3 were replaced with a marker gene (*his7*^+^ or *ura4*^+^) in a nonstandard strain JP1225 background^21^. Strain *972* was crossed with *SD5*, and the first or second filial progeny were analyzed for the presence or absence of each *SH* region by pulse-field gel electrophoresis (PFGE) followed by Southern blotting (Fig. 2a–d; also see “Methods” section). We screened for strains that exhibit a single band of telomere-associated sequence (TAS)^29^ (i.e., *SH*). We obtained strains that contain a single *SH* region of *972* and named them *972SD4[1L*+, *1R*+, *2L*+, or *2R*+*]*, since they carry deletions of four *SH* regions in the original *SD5* and one intact *SH* region from *972*. Each intact *SH* region in *972SD4* was named as *972SD4-SH1L*, *972SD4-SH1R*, *972SD4-SH2L*, or *972SD4-SH2R*.

### Cloning and sequencing of *SH-P* regions

From the data in PomBase, multiple common segments aligned in a mosaic pattern were expected for the *SH-P* region (see below). In order to accurately assemble such repetitive sequences, we amplified the *SH-P* region (~5 kb) in each *972SD4* by PCR and cloned into a vector. Partial deletion series of the *SH-P* fragments was constructed by digesting the plasmids with restriction enzymes followed by treatment with exo- and endo-nucleases. Re-circularized plasmids that carry the *SH-P* fragments with various lengths were sequenced using primers that anneal to the vector (Supplementary Fig. 2, see “Methods” section).

### *SH-P* regions exhibit highly variable mosaic structures consisting of common segments

**Fig. 3:**
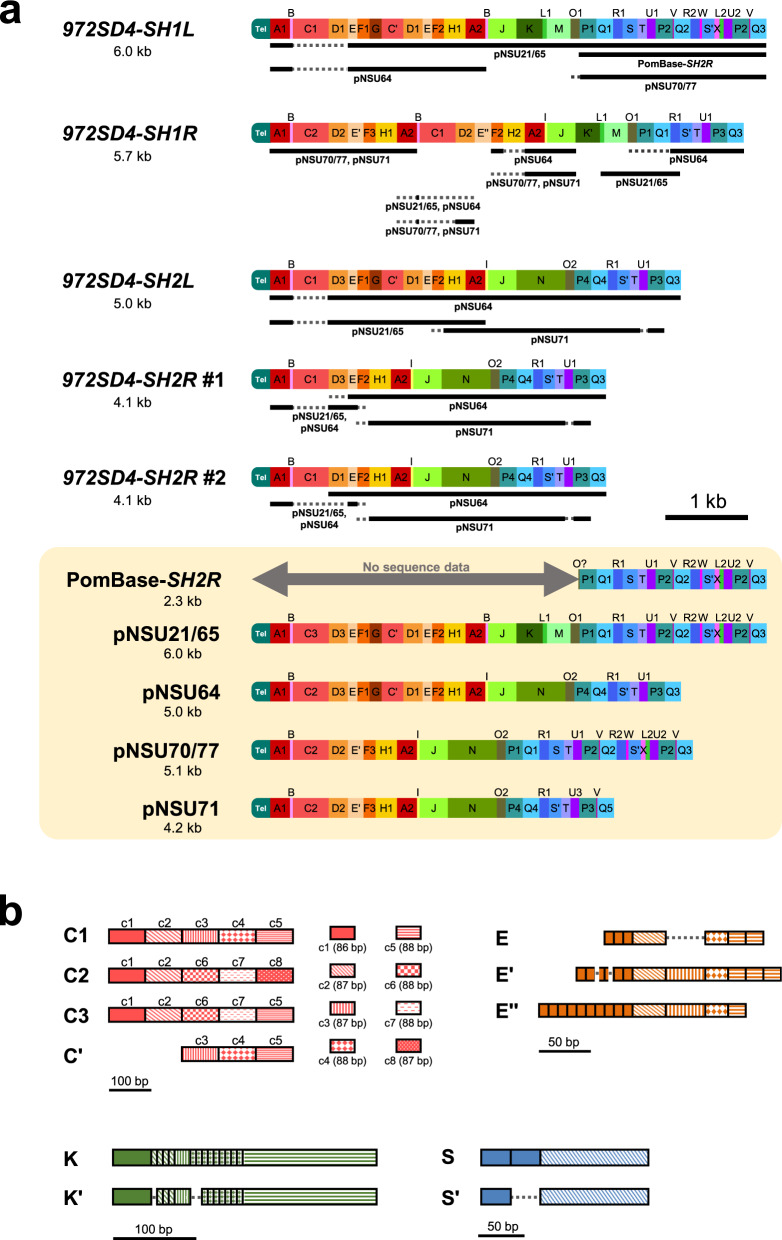
Highly variable *SH-P* regions consist of common segments in strain *972*. **a** Schematics of *SH-P* regions. The *SH-P* sequences were classified into common segments (A–X) and their variants (see Supplementary Fig. 3 for each sequence). *SH-P* regions of *972SD4* strains, a partial *SH2R* region in PomBase (PomBase-*SH2R*), and *SH-P* regions of a pNSU series in PomBase are shown. The total length of each *SH-P* region is indicated. Note that *972SD4-SH1L*, *972SD4-SH1R*, and *972SD4-SH2L* obtained from two independent *972SD4* strains (i.e., #1 and #2 clones) are identical, whereas *972SD4-SH2R* #1 and #2 contain different variants D, D1 and D3. Black bars indicate relatively long regions that show the same segments and variants with those of pNSUs or PomBase-*SH2R*, whereas gray dotted lines indicate regions that show the same segments but different variants. **b** Subtypes of C, E, K, and S consist of common sequence motifs with different copy numbers. The boxes with the same color and pattern show 100% sequence identity. Segment C consists of three variants, C1–3, that are composed of homologous sequence motifs, c1–8, that show >82% identities with each other (see Supplementary Fig. 3c, c* for details).

We classified the sequences of *SH-P* regions of the *972SD4* strains, a part of *SH2R* in PomBase (PomBase-*SH2R*), and insertions in the pNSU series in PomBase^25^ into common segments (A–X) as follows. First, the sequences of *SH-P* regions were classified into common segments that meet the criteria of ≥ 50 bp and >95% identity using NCBI nucleotide BLAST (blastn) program (v2.10.0+, https://blast.ncbi.nlm.nih.gov/Blast.cgi). Then, gaps between the segments were classified into additional segments that meet the criteria ≥14 bp and >95% identity. Segments were classified into variants (e.g., A1 and A2) that meet the criteria of 100% identity (Fig. 3a and Supplementary Fig. 3). To reduce the number of common segments, exceptional rules were applied for subtypes C (C1–3) and C′, E, E′, and E′′, K and K′, and S and S′, which contain different copies of several common sequence motifs that show 100% identities except for motifs in subtype C, c1–8 (Fig. 3b and Supplementary Fig. 3). *SH-P* regions in two independent strains (#1 and #2) of *972SD4[1L*+*]*, *972SD4[1R*+*]*, and *972SD4[2L*+*]* exhibit 100% sequence identity, suggesting that no mutation or rearrangement have been introduced to the *SH-P* sequences of these *972SD4* strains during crossing, amplification by PCR, cloning using *Escherichia coli* (*E. coli*), and construction of serial deletion mutants. In contrast, two strains of *972SD4[2R*+*]* contain different variants of segment D, D1 and D3, which show differences in two nucleotides, suggesting that the two point mutations at segment D and/or interchromosomal recombination have occurred in *972* or *972SD4[2R*+*]* (Fig. 3a and Supplementary Fig. 3d).

We found that none of the *SH-P* regions of *972SD4* show the same pattern in the alignment of segments to each other; however, two pairs of regions, *972SD4-SH1L* and pNSU21/65, and *972SD4-SH2L* and pNSU64, each exhibit the same segment patterns over the whole *SH-P* regions, suggesting the possibility that these pairs were derived from the same subtelomeres. However, the compositions of the C and D variants are different (C1 vs. C2 or C3, and D1 vs. D3; Fig. 3a, gray dotted lines). We found that segments C in *972SD4* strains are particularly different from those of pNSUs (Fig. 3a, gray dotted lines); i.e., variant C1 is the majority in the *SH-P* sequences of *972SD4*, whereas variant C2 is the majority in those of pNSUs. These data suggest that segment C is prone to mutation and recombination possibly due to its highly repetitive structure (Fig. 3b).

In contrast to *972SD4-SH1L* and *972SD4-SH2L*, *972SD4-SH1R* and *972SD4-SH2R* show combinations of pNSU patterns (Fig. 3a). Moreover, subtypes E′′ and K′, and variant H2 are unique to *972SD4-SH1R* among the *SH-P* sequences of *972SD4* and PomBase-*972*, suggesting that multiple times of mutation and recombination have occurred at *972SD4-SH1R*. Surprisingly, *972SD4-SH2R* exhibits a pattern different from a part of the PomBase*-SH2R* sequence; indeed, the pattern of PomBase*-SH2R* is found in a part of *972SD4-SH1L*, implying that *SH1L* and *SH2R* have exchanged their chromosomal positions over repeated rounds of cell divisions.

Although we identified several variants for each segment, these variants are not randomly combined, and partial sequences show the same alignments; for instance, there are two common alignments: P1-Q1-----V-Q3 (in *972SD4-SH1L*, PomBase-*SH2R*, pNSU21/65, and pNSU70/77) and E-F2-----P3-Q3 (in *972SD4-SH2L*, *972SD4-SH2R* #1, *972SD4-SH2R* #2, and pNSU64). Overall changes in the segment and variant compositions imply that *SH-P* regions are prone to nucleotide change and chromosome rearrangement.

### *SH-P* regions exhibit high variation in segmental arrangement among *972* strains and natural isolates of *S. pombe*

**Fig. 4:**
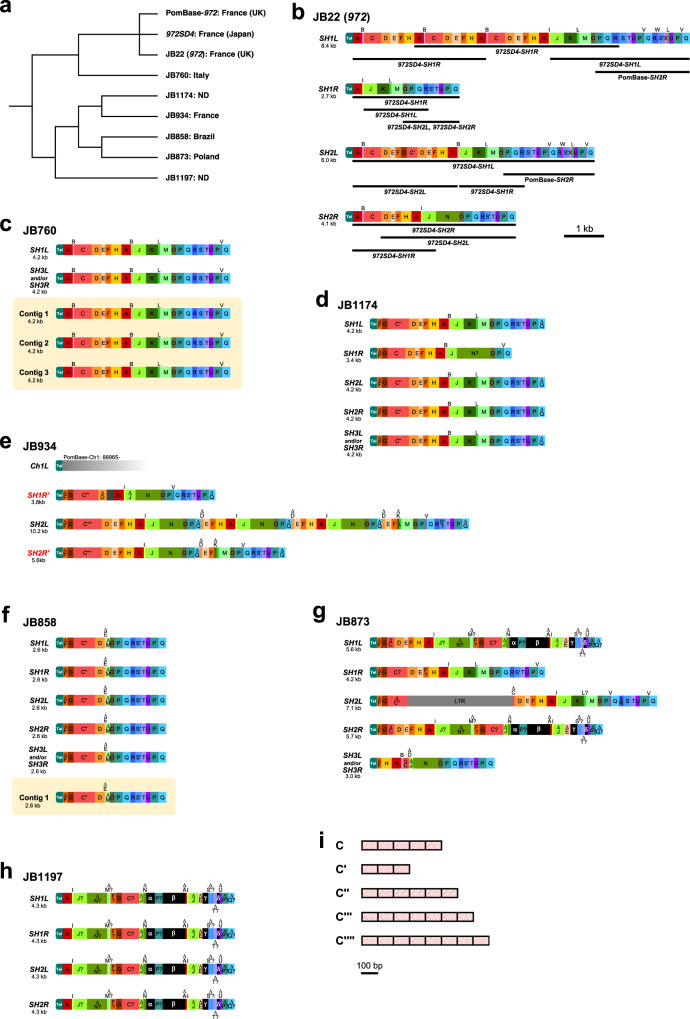
High variation in *SH-P* regions in diverse *S. pombe* strains. **a** Phylogenetic relationships among *972* and JB strains based on genome-wide sequences according to a previous study by Tusso et al.^30^. Locations where the strains were collected are indicated^33^. ND, not defined. Regarding *972*, locations where the strains are stocked and analyzed are indicated in parentheses. **b** Schematics of the *SH-P* sequences in strain JB22 (*972*) with common segments. Sequences of indicated segments exhibit 90% or higher identities with those of PomBase or *972SD4*. Black bars indicate relatively long regions that show the same segmental arrangements with those of *972SD4* and PomBase-*SH2R*. **c** Schematics of the *SH-P* sequences in strain JB760. Second bar shows an *SH-P* region of *SH3L* and/or *SH3R* of Ch3. Contigs 1–3 include *SH-P* regions that are not allocated to specific subtelomeres. **d** Schematics of the *SH-P* sequences in strain JB1174. C′′ is a subtype of segment C which contains six units of c motifs (see Figs. 3b and 4i). ∆ indicates a partial sequence of each segment with no less than 10 bp decrease in total length. “?” indicates 75–90% sequence identity with the corresponding segment. **e** Schematics of the *SH-P* sequences in strain JB934. C′′′ and C′′′′, subtypes of segment C, contain seven and eight units of c motifs, respectively (see Figs. 3b and 4i). *SH1R*′ and *SH2R*′ (highlighted in red) indicate that they are located at *subtel2R* and *subtel1R* in JB934, but overall sequences of telomere-side regions of *Ch2R* and *Ch1R* are homologous to those *Ch1R* and *Ch2R* in *972*, respectively (see Supplementary Fig. 4). Dark gray box in *SH1R*′ indicates a sequence that has not been found in *972* strains and is not shared by the subtelomeres in other JB strains. **f** Schematics of the *SH-P* sequences in strain JB858. Contig 1 includes an *SH-P* region that is not allocated to a specific subtelomere. **g** Schematics of the *SH-P* sequences in strain JB873. Black boxes indicate common segments shared by some of JB strains, although their sequences have not been found in *972* strains (see Supplementary Fig. 5 for their sequences). Note that a partial LTR sequence is inserted at *SH2L*. **h** Schematics of the *SH-P* sequences in strain JB1197. **i** Subtypes of segment C. Note that the types of c motifs are not specified due to the relatively low reliability of sequences by long-read DNA sequencing.

To elucidate how *SH-P* regions have changed in the course of culturing or evolution, we next extracted uncharacterized sequences of the *SH-P* regions in various natural isolates of *S. pombe* in J. Bähler’s laboratory in UK (JB strains), using previous raw data by Tusso et al. (long-read sequencing using Nanopore MinION and PacBio RS II)^30^ (Fig. 4a, see “Methods” section for details). Our analyses on chromosome configuration suggested that chromosome end fragment of *Ch1R* has been swapped with that of *Ch2R* in JB934 in comparison with *972*, as described previously (Supplementary Fig. 4)^30^. Although long-read sequencing data lack accuracy, we were able to analyze patterns of segments (A–X) and subtypes, but not at the variant level (see figure legends of Fig. 4 for details).

JB22 (*972*), another clone of *972*, has *SH* regions only at the ends of Ch1 and Ch2, not in Ch3, as in the strain *972* in our laboratory. However, *SH-P* regions in JB22 (*972*) have different segment patterns and lengths from those in *972SD4* except for *SH2R*, which does not contain a segment alignment homologous with PomBase-*SH2R*. These data indicate that chromosome rearrangement occurs frequently in *SH-P* regions during culturing in laboratories (Figs. 3a and 4b).

Segment patterns and lengths of *SH-P* regions in each *972* strain are divergent, whereas JB760, JB1174 (except for *SH1R*), JB858, and JB1197 exhibit almost uniform segment patterns and lengths among the subtelomeres (Fig. 4c, d, f, h). It is unclear which type is the original *SH-P* in *S. pombe*; however, the divergent property is likely ancestral because repeated interchromosomal rearrangements will result in uniform patterns of *SH-P* regions.

We detected some sequences that have not been found in *972* strains but shared by several subtelomeres in JB strains (indicated by black boxes α–δ in Fig. 4 and Supplementary Fig. 5). Particularly, partial sequences of *SH1L* and *SH2R* in JB873 (segments A-I-----∆P-∆Q containing black boxes) are highly homologous with the *SH-P* sequences in JB1197 (Fig. 4g, h). Furthermore, JB934 does not possess any *SH* sequence in *Ch1L*, and JB873 contains partial sequence of long terminal repeat (LTR) of retrotransposon in the *SH-P* region of *SH2L* (Fig. 4e, g).

Interestingly, there are two major types of structures at chromosome ends, telomere-segment A-segment B (Tel-A-B) and telomere-partial segment F-segment G (Tel-∆F-G). Tel-A-B is found in *972* and JB760 (Figs. 3a and 4b, c), whereas Tel-∆F-G is found in JB1174, JB934, JB858, and JB873 (Fig. 4d–g). We found that the sequences of segments A and B is highly similar to those of partial segment F and segment G, suggesting the possibility that homologous recombination (HR) between them resulted in a Tel-∆F-G structure (Supplementary Fig. 6a). Other possibilities are that nucleotide changes have occurred in either A-B or ∆F-G (Supplementary Fig. 6b), or that the G-rich sequences located at the telomere-proximal ends of segment A and ∆F formed G-quadruplex-like structures and stalled replication fork progression^31^, which resulted in chromosome breakage accompanied by de novo telomere synthesis (Supplementary Figs. 3 and 6c). Overall diversity of the *SH-P* regions in JB strains further support our hypothesis that *SH-P* regions are hot spots for genome evolution.

### *SH-D* regions in *972* strains are variable and accumulate insertions and deletions

**Fig. 5:**
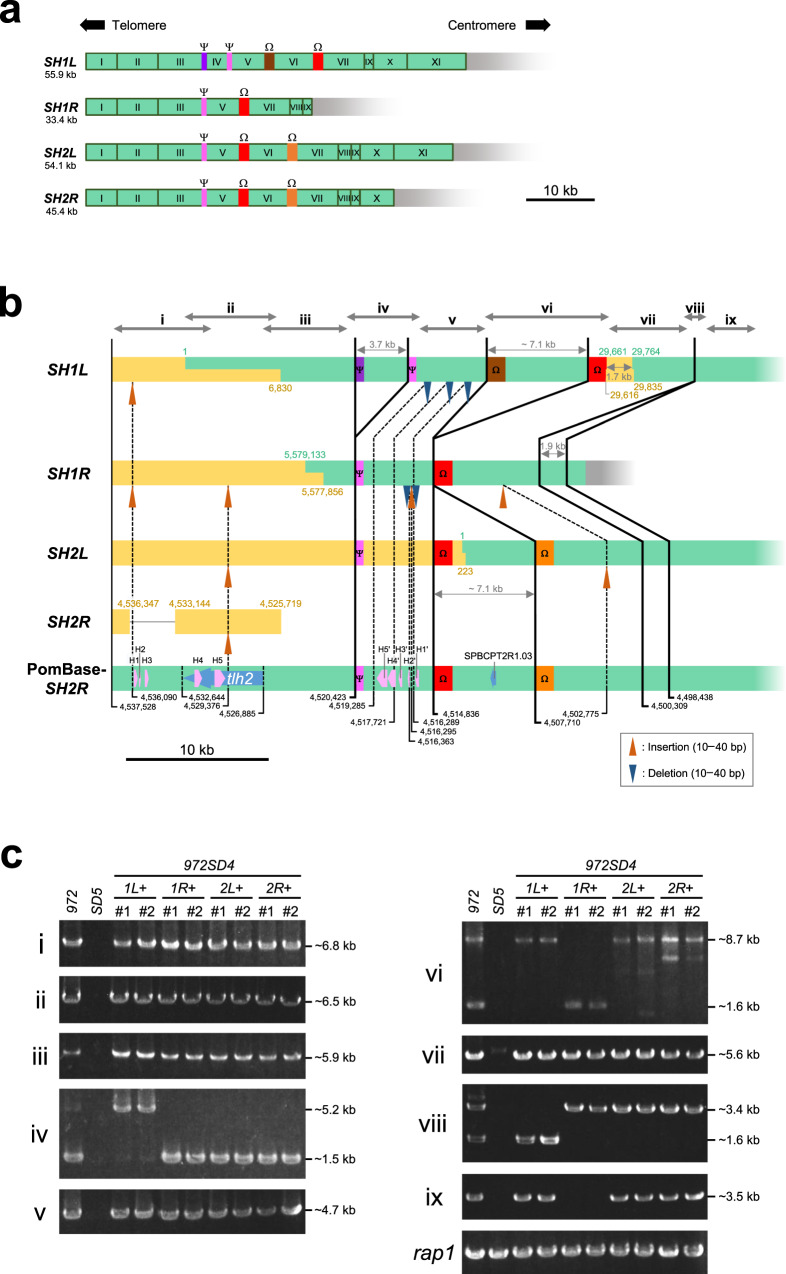
Sequence variations of *SH-D* regions in *972SD4* and PomBase-*972*. **a** Schematic illustration of homologous block (I–XI) and box (Ψ and Ω) sequences in the *SH-D* regions in *972SD4* and PomBase-*972* (see Supplementary Table 1 for their positions in PomBase). All *SH* sequences are aligned with telomeres to the left and centromeres to the right. **b** Comparison of *SH-D* regions between subtelomeres. Sequences of *SH1L*, *SH1R*, and *SH2L* in PomBase (indicated by green boxes) were combined with sequences newly determined in this study (yellow boxes, showing #1 clones of each *SH* sequence), whereas newly sequenced *SH2R* fragments are shown separately with PomBase-*SH2R*. Green and yellow numbers indicate positions in each chromosome in PomBase at the ends of overlaps between PomBase and newly determined regions. All *SH* sequences are aligned with telomeres to the left and centromeres to the right. Insertions (orange arrowheads) or deletions (blue arrowheads) of 10–40 bp are shown in comparison with PomBase*-SH2R* (thin dotted lines). Insertions and deletions of <10 bp are omitted in this panel. The thick lines connect the corresponding positions in chromosomes that are boundaries of the long regions with sequence alterations. Purple and pink boxes (Ψ) and brown, red, and orange boxes (Ω) indicate homologous sequences at the ends of the long sequence changes, 3.7 and ~7.1 kb, respectively (see Supplementary Fig. 7 for the sequences and identities between them). Numbers in black indicate chromosomal position in PomBase-*SH2R*. Chromosomal positions of insertions and deletions are indicated by the positions in PomBase-*SH2R* that are immediately before the changes (closer to the telomeres). Blue arrows in PomBase-*SH2R* indicate the positions of ORFs for *tlh2*^+^ and *SPBCPT2R1.03*. Pale pink arrows in PomBase-*SH2R* indicate homologous regions (H1–5 and their inverted sequences, H1′–5′) that are utilized for chromosome fusion when telomeres are lost^53^. Gray arrows at the top indicate ranges of PCR products i–ix analyzed in **c**. Note that the PCR fragments i–ix in **b** roughly correspond to blocks I–IX in **a**. **c** Lengths of the *SH-D* regions in *972SD4* strains. DNA fragments i–ix were amplified by PCR using genomic DNAs of the *972* (JK107), *SD5* (ST3479), and two independent *972SD4* strains (#1 and #2) as templates. Approximate DNA lengths estimated by the sequences in PomBase and *972SD4* are indicated on the right. The *rap1* locus was amplified as a control. PCRs were performed at least twice.

We also determined sequences of *SH-D* regions in *972SD4* strains by integrating the partial *SH-D* sequences in PomBase and our newly determined *SH-D* sequences. The integrated *SH-D* sequences were classified into common block sequences I–XI (>90% identities between the sequences of the same blocks), and supplemental homologous box sequences Ψ and Ω (Fig. 5a and Supplementary Table 1). In contrast to the *SH-P* regions, the *SH-D* regions in strain *972* do not show duplications or differences in orders of blocks I–XI. However, we found differences in length; multiple insertions or deletions were identified in these regions (Fig. 5b).

There are three big differences between subtelomeres (Fig. 5b, thick lines; note that part of them has been described in a previous study^32^). First, there are 3.7 kb deletions in *SH1R*, *SH2L*, and *SH2R* at position 4,520,423 of PomBase-*SH2R*. Second, there is a 7.1 kb deletion in *SH1R* at nucleotides 4,514,836–4,507,710 of PomBase-*SH2R*. This deletion was detected in the three independent strains of *972SD4[1R*+*]* by PFGE-Southern analysis (Fig. 2d). Third, there is a 1.9 kb deletion in *SH1L* at nucleotides 4,500,309–4,498,438 of PomBase-*SH2R*.

Intriguingly, boxes Ψ exist at the ends of the 3.7 kb change (Fig. 5a, b, purple and pink boxes, Supplementary Fig. 7a, b, and Supplementary Table 1). Similarly, boxes Ω exist at the ends of 7.1 kb changes (Fig. 5a, b, brown, red, and orange boxes, Supplementary Fig. 7c, d, and Supplementary Table 1). These data imply that the deletions and/or insertions have occurred using these homologous sequences. It is noteworthy that among Ω sequences that are indicated by different colored boxes, there are multiple insertions and deletions in the region where various repeat sequences are arranged intricately, and the sequence of *ΩSH1L-L* (indicated by a brown box) shows lowest sequence identity (83–85%) with other red or orange boxes (Supplementary Fig. 7c, d).

There are also smaller insertions or deletions in *SH-D* regions (Fig. 5b, thin dotted lines for changes of 10–40 bp compared with PomBase-*SH2R*). Many of these are observed in no less than two *SH-D* regions, suggesting that these changes have been copied to other *SH* regions by chromosome rearrangement. It is also noteworthy that the newly sequenced *SH2R* in *972SD4[2R*+*]* contains an insertion at position 4,529,376 of PomBase-*SH2R*, indicating that this insertion has been introduced to *SH2R* of strain *972* in laboratories after separated from PomBase-*972*.

To examine stability of the *SH-D* regions, their DNA structures in two independent strains of *972SD4[1L*+*]*, *972SD4[1R*+*]*, *972SD4[2L*+*]*, and *972SD4[2R*+*]* were analyzed by PCR using multiple primer sets (PCR products i–ix are indicated by gray arrows in Fig. 5b, top. Note that the fragments i–ix in Fig. 5b roughly correspond to blocks I–IX in Fig. 5a). We found that lengths of the all PCR products matched those predicted from PomBase sequence (Fig. 5c), indicating that the overall DNA structures of *SH-D* regions are stably maintained between PomBase-*972* and *972SD4* strains in contrast to those of *SH-P* regions. We calculated the full length of each *SH* region (*SH-P* and *SH-D*): *SH1L*, 61.9 kb; *SH1R*, 39.1 kb; *SH2L*, 59.1 kb; and *SH2R*, 49.5 kb, with *SH1R* having the shortest *SH* sequence, although these lengths are likely to change through chromosome rearrangements at *SH* regions.

**Fig. 6:**
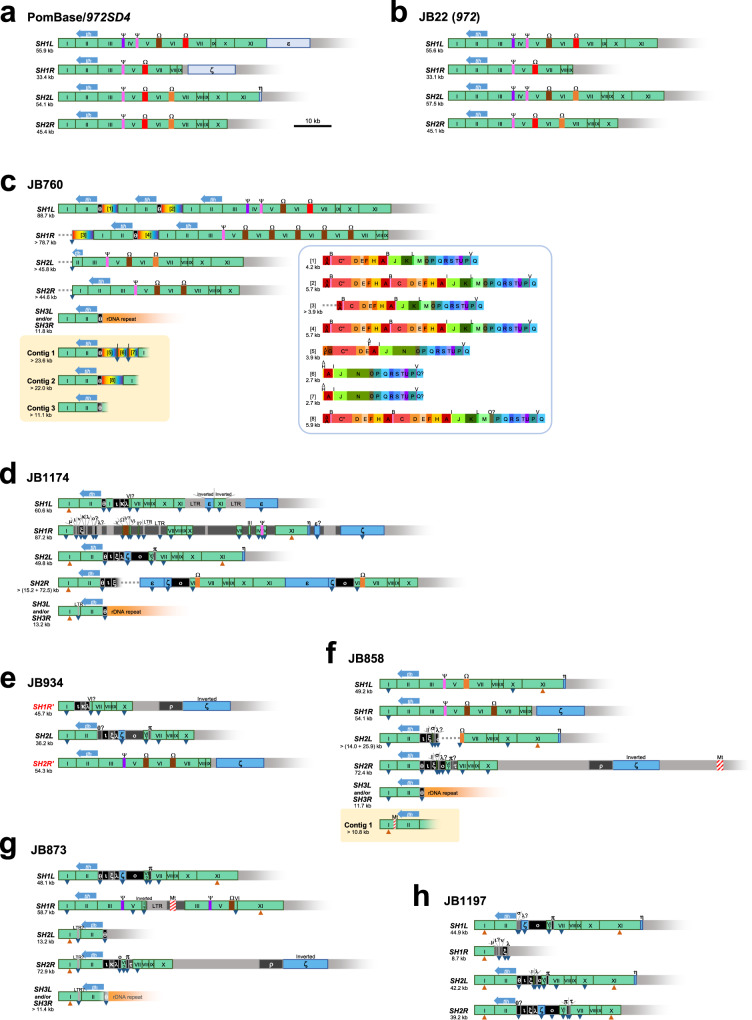
Sequence variations of *SH-D* regions in JB strains. **a** Schematic illustration of homologous block and box sequences in the *SH-D* regions in *972SD4* and PomBase-*972*. Note that sequences of pale blue boxes, ε–η, in the *SU* regions are shared by multiple subtelomeres in some of JB strains; and thus, they are defined as *SH* (see Fig. 6d–h, blue boxes). ORFs of the *tlh* genes located in block II are indicated by blue arrows. **b** Schematics of the *SH-D* sequences in strain JB22 (*972*) in common blocks and boxes. Sequences of indicated blocks and boxes exhibit at least 90% identities with those of PomBase-*972*. Total length of each *SH-D* region is indicated. **c** Schematics of the *SH-D* sequences in strain JB760. Black boxes indicate common sequences that are shared by some other JB strains but not found in *972* strains. Note that the sequence of θ is homologous with that of SAS (see main text and Supplementary Fig. 1 for the details). Contigs 1–3 are the same as those in Fig. 4c, respectively. Boxes in rainbow color [1]–[8], *SH-P*-like sequences; “?”, sequence with <90% identity but substantial similarity with the corresponding segment, block or box (E-value < 10^−10^ in NCBI nucleotide BLAST [blastn] search); blue triangle, shorter length of the corresponding segment, block or box; gray dotted line, no sequence information available; blocks or boxes with gradation, unreliable sequence information with less than five reads. **d** Schematics of the *SH-D* sequences in strain JB1174. Inverted, opposite sequence direction from that in the corresponding block or box in *972*; blue box, sequence shared by multiple subtelomeres as *SH* in some JB strains; dark gray box, sequence that has not been found in PomBase and not shared by the other JB strains; orange triangle, longer length of the corresponding block or box. **e** Schematics of the *SH-D* sequences in strain JB934. **f** Schematics of the *SH-D* sequences in strain JB858. Contig 1 is the same as that in Fig. 4f. Shaded box with red lines, an insertion of a part of mitochondrial genome. **g** Schematics of the *SH-D* sequences in strain JB873. **h** Schematics of the *SH-D* sequences in strain JB1197.

To examine evolutional change of *SH-D* regions, we next analyzed *SH-D* regions in JB strains using previous NGS data (long-read sequencing) by Tusso et al.^30^. The *SH-D* regions in JB22 (*972*) show clear differences compared with those in PomBase-*972* and *972SD4*, although the overall pattern of common blocks is very similar (Fig. 6a, b). Block IV and a purple box (Ψ) sequences are present in both *SH1L* and *SH2L* in JB22 (*972*), but not in *SH2L* in *972SD4*. In addition, a brown box sequence (Ω) resides between blocks V and VI in *SH1L* and *SH2L* in JB22 (*972*), whereas the sequence is replaced by a red box sequence (Ω) in *SH2L* in *972SD4*. These data suggest the possibility that HR has occurred between the *SH-D* regions in *SH1L* and *SH2L* in JB22 (*972*). Thus, the *SH-D* regions in *972* strains are not highly stable; rather, changeable via recombination.

### *SH-D* regions exhibit striking variations among JB strains

The *SH-D* regions of the other JB strains show striking variation in numbers and orders of block sequences. Furthermore, various box sequences that are not categorized as *SH-D* in *972* strains are shared by multiple *SH-D* regions in JB strains (Fig. 6c–h). It is noteworthy that *972* strains have no *SH* sequence in Ch3, whereas JB760, JB1174, JB858, and JB873 possess *SH-P* and *SH-D* sequences in Ch3 (Figs. 4 and 6). Intriguingly, some descendent strains of *972* also possess *SH* sequences at either or both ends of Ch3 (Supplementary Fig. 1, KYP33 and JP1225). These data suggest two possibilities; one is that another standard strain *975* (*h*^+^) contains *SH* sequence(s) in Ch3, and it has been transferred to descendent strains via mating and meiosis. Another is that *SH* regions in Ch1 or Ch2 have been translocated to Ch3 via interchromosomal recombination. In fact, the *SH-P* sequence in JP1225 shows high similarity with those of *972SD4*-*SH2R* and pNSU71 (Fig. 3a and Supplementary Fig. 1c). However, the former is more possible because of the existence of a black box θ sequence associated with *SH-D* in Ch3, which has not been found in *972* strains (see below).

Interestingly, multiple copies of brown box sequences (Ω) and *SH-P* sequences [1]–[8] are found in the *SH-D* sequence in JB760 (Fig. 6c). Moreover, adjacent to block II is a black box θ. The alignment of I-II-θ is also found in Ch3 of JB1174, JB858, and JB873, although partial LTR sequences are inserted in block I in JB1174 and JB873 (Fig. 6d, f, g). Intriguingly, the sequence of box θ is almost identical with that of SAS (spanning ~1.1 kb), which was identified as a subtelomere-associated sequence adjacent to block II in Ch3 in the descendent strains of *972* previously^21,27^ (Supplementary Fig. 1b–d). Thus, structures of *SH-D* regions in Ch3 are highly conserved in *S. pombe* in contrast to other parts of *SH-D* possibly because the ends of Ch3 are located in the nucleolus apart from those of Ch1 and Ch2 located in the nucleus in vegetatively growing cells^17^, which may restrain interchromosomal recombination between Ch3 and Ch1 or Ch2.

The *SH-D* regions of JB strains except for JB22 (*972*) and JB760 showed high variation in their compositions and lengths; however, they share some common features. (1) Blocks I and II are highly conserved. (2) Multiple copies of parts of the *SU* regions in *972* (indicated by pale blue boxes, ε–η, in Fig. 6a, see Supplementary Table 1 for their positions) are found in the *SH* regions in these strains (indicated by blue boxes in Fig. 6). (3) Multiple black boxes, θ–τ, which are not found in *972* are also shared by the subtelomeres of JB strains except for JB22 (*972*) (Fig. 6). (4) Blocks I and XI (green) and boxes ε (blue) and ζ (blue) are found inverted compared with those in *972* (Fig. 6d, e, g). (5) Surprisingly, the *SU* region of JB858 and the *SH-D* regions of JB858 and JB873 contain sequences of parts of mitochondrial genome (indicated by Mt in Fig. 6f, g). The overall changes in *SH-D* regions in JB strains indicate that complexed chromosome rearrangements have occurred in the course of evolution of *S. pombe* even after strain *972* has been isolated.

### Subtelomeres are hot spots for mutations

**Fig. 7:**
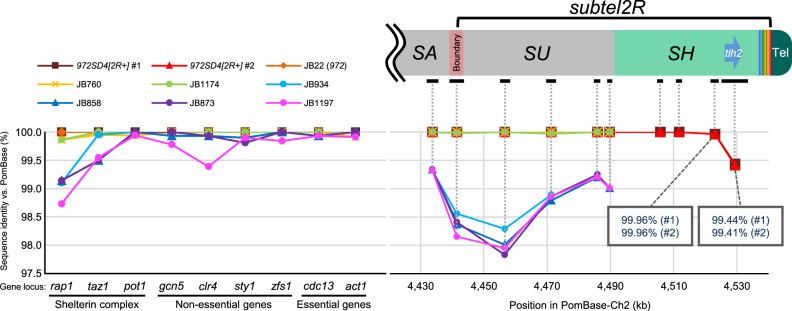
Subtelomeres are hot spots for sequence variation. Sequence identities between PomBase-*972* and other *S. pombe* strains used in this study. Sequences of the *SH-D* and *SU* regions, the subtelomere boundary, and the *SA* region in *Ch2R*, and those of other gene loci of three categories were analyzed (see Supplementary Table 2 for the details). Note that two independent *972SD4[2R*+*]* strains (clones #1 and #2) contain different sequences around the *tlh2* gene locus.

Given that the *SH-P* regions of *972SD4* contain multiple nucleotide changes compared with those in PomBase-*972* (Fig. 3 and Supplementary Fig. 3), we examined whether mutation rates are specifically high in subtelomeres. First, sequences of multiple loci in the *SH-D* of *SH2R* in two independent *972SD4[2R*+*]* strains (#1 and #2) were determined and compared with those of PomBase-*972* (Fig. 7 and Supplementary Table 2). We found that regions around the *tlh2* gene locus exhibit high rates of mutations. Most of them are point mutations, but some are changes of numbers of repeat sequences, such as [T]_n_. It should be noted that the two *972SD4[2R*+*]* strains possess different sequences in the *tlh2* locus (99.44% vs. 99.41% identities with PomBase). In contrast, the telomere-distal half of *SH-D* region and all chromosomal regions outside of *SH*, i.e., the *SU* region, the subtelomere boundary region, the *SA* region (see Fig. 1a), and various gene loci in Ch1 or Ch2 in the two *972SD4[2R*+*]* strains show 100% sequence identities with those in PomBase-*972*, indicating strict preservation of their DNA sequences through repeated rounds of cell division. These data suggest that the telomere-proximal half of *SH-D* regions (~20 kb), as well as *SH-P* regions are particularly prone to the accumulation of mutations.

We next examined mutation rates in JB strains using previous NGS data (long-read sequencing by Tusso et al.^30^ and short-read sequencing with high accuracy using Illumina HiSeq 2000 by Jeffares et al.^33^, see “Methods” section for details). Only the chromosomal loci outside of *SH* were analyzed because *SH* regions are not distinguishable using short reads. We found that JB22 (*972*) showed 100% sequence identities with PomBase-*972* at the all loci examined (Fig. 7 and Supplementary Table 2), indicating that genome integrity is strictly maintained through repeated rounds of cell division at these loci.

In striking contrast, JB strains other than JB22 (*972*) exhibit high mutation rates at the *SU*, subtelomere boundary, and *SA* regions in *Ch2R* (Fig. 7 and Supplementary Table 2), indicating that subtelomeres are prone to nucleotide changes during the long-time course of *S. pombe* evolution. Interestingly, some JB strains show relatively high mutation rates at the chromosome loci of nonessential genes, especially the *rap1* gene (Fig. 7 and Supplementary Table 2). Rap1 is a subunit of the shelterin complex, which protects chromosome ends and regulates various telomere functions^15,34^. Rap1 is recruited to telomeres partly through interaction with a telomere DNA-binding protein Taz1 and associates with multiple proteins to regulate various telomere functions^34–36^. Amino acid changes of Rap1 are rarely found in the regions for interactions with its partners (Supplementary Fig. 8). We found one amino acid change, glutamic acid (E) 671 to arginine, in the RCT (Rap1 C-terminal) domain of Rap1, which mediates interaction of Rap1 with Taz1; however, it was suggested that E671 is not involved in their direct interaction^37^. Moreover, one amino acid change, E424 to alanine, is found in the DD (dimerization domain) of Taz1, which is important for Taz1 binding to telomere DNA; however, E424 is located outside of the direct interaction domain^38^. Furthermore, we found that the sequences of telomere repeats in JB strains are highly similar to those in *972* strains. Thus, it seems that the principal functions of Rap1 and Taz1 are conserved during the course of *S. pombe* evolution. It is likely that the higher mutation rates in the *rap1* and *taz1* genes are because their gene products are more tolerant to amino acid changes than other gene products.

### Identification of additional members of the subtelomeric RecQ helicase gene family

**Fig. 8:**
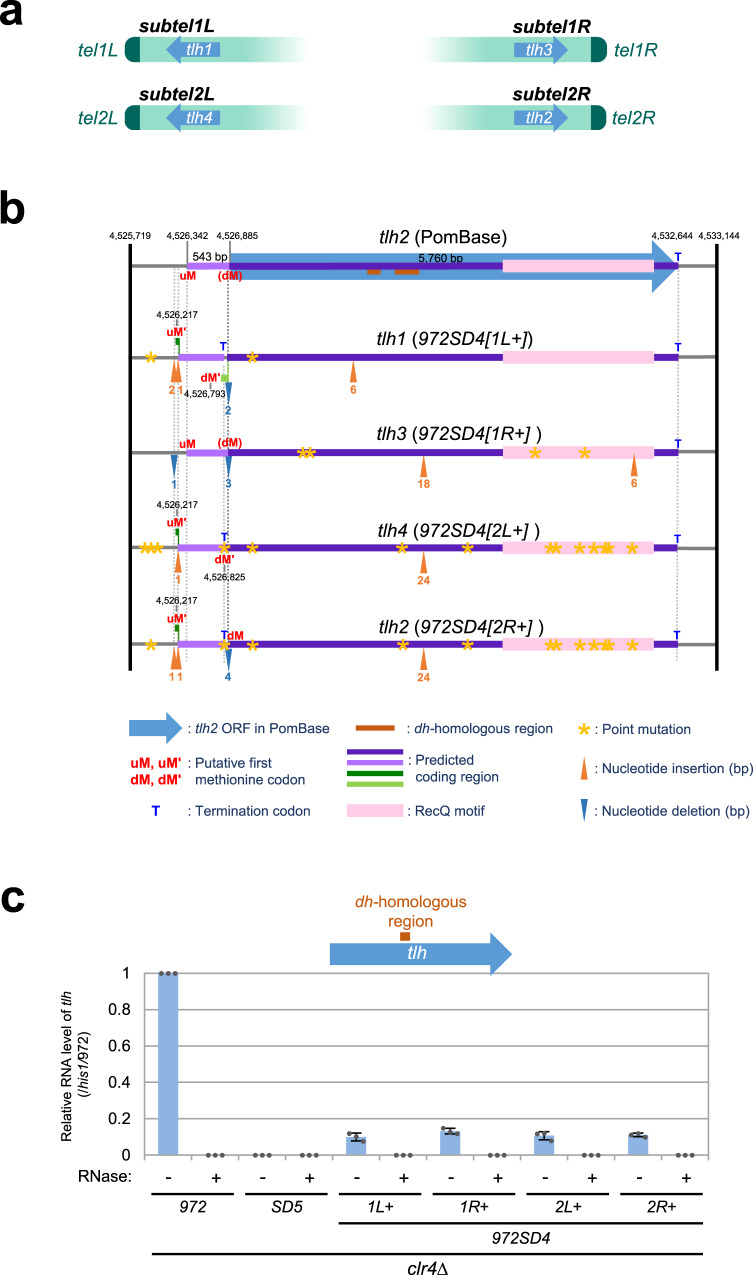
Identification of *tlh* genes with multiple mutations. **a** Identification of *tlh3* and *tlh4*. Chromosomal locations of the four *tlh* genes are shown. **b** Summary of predicted ORFs and nucleotide changes in the *tlh* genes of *972SD4* compared with the *tlh2* gene in PomBase. The top panel shows the *tlh2* locus in PomBase. Seven digit numbers indicate chromosomal positions on Ch2 in PomBase. Blue arrow with a purple line, the *tlh2* ORF defined in PomBase; brown bar, *dh*-homologous region; light purple line, predicted coding region which is in-frame with that of the *tlh2* ORF in PomBase; light or dark green line, predicted coding region in the reading frame different from that of the predicted *tlh2* ORF in PomBase; yellow star, point mutation (no frame shift); orange arrowhead, nucleotide insertion (numbers of inserted nucleotides are shown below); blue arrowhead, nucleotide deletion (numbers of deleted nucleotides are shown below); dM the original first methionine codon of the *tlh2* ORF in PomBase, uM putative first methionine codon upstream of dM for PomBase-*tlh2*, uM′ putative first methionine codon for the short ORF, dM′ putative first methionine codon for the long ORF, T termination codon. **c** Expression of RNA of the *dh*-homologous region in the *tlh* genes in strains *972*, *SD5*, and *972SD4* in a *clr4*∆ background was analyzed by quantitative RT-PCR in the presence (+) or absence (−) of RNase. Expression level of *tlh*^+^ relative to that of *his1*^+^ was normalized to that in *972* (−RNase). Data are presented as mean values ± SD (standard deviations). *N* = 3 biologically independent experiments (each value is indicated by a gray dot).

In genome sequences in PomBase*-972*, there are two RecQ helicase genes, *tlh1* (partial) and *tlh2*, which have been allocated to *SH1L* and *SH2R*, respectively. Parts of the DNA sequences of *tlh1/2* are homologous with the *dh* repeat sequence of pericentromeres and serve as templates for small interfering RNA (siRNA) produced by RNA interference (RNAi) machinery; further, the siRNA participates in the initiation of subtelomeric heterochromatin formation^22,23^. Our sequencing data of *SH* regions newly identified two members of the *tlh* gene family, *tlh3* and *tlh4*, in *SH1R* and *SH2L*, respectively. Thus, genome of strain *972* contains four *tlh* genes in total (Fig. 8a and Supplementary Fig. 9).

The descendent strains of *972* possess additional *tlh* ORF(s) in Ch3 (Supplementary Fig. 1), that may have transferred from their other chromosome ends or from mating partners. We also found multiple putative *tlh* genes in the *SH-D* regions of JB strains (Fig. 6, blue arrows). All JB strains possess at least two *tlh* genes because *tlh* genes are located in block II, which is highly conserved among the strains.

### The *tlh* genes contain multiple nucleotide changes

Examination of the *tlh* sequences raised the possibility that the ORF for the *tlh2* gene is not properly defined in PomBase. In the sequence of PomBase-*SH2R*, there is an in-frame methionine (Met) codon (uMet [upstream Met]) 543 bases (corresponding to 181 amino acids) upstream of the original first Met codon (dMet [downstream Met]) that was defined by PomBase (Fig. 8b and Supplementary Fig. 9). The sequences surrounding these Met codons match the Kozak consensus sequence (A/GNNATGG, the initiation codon underlined), which participates in the initiation of translation in eukaryotes^39^, suggesting that the uMet may be the true initiation codon for translation.

Unexpectedly, each of the newly sequenced *tlh1–4* genes of *972SD4* was found to contain multiple nucleotide changes (i.e., point mutations, insertions, and deletions) compared with the *tlh2* gene in PomBase (Fig. 8b and Supplementary Fig. 9). There are tandem repeat sequences at the loci where insertion occurred in the long coding region: [ATGACA]_n_ at the most N-terminal insertion (the 6 bp insertion), [CGACAA]_n_ at the middle insertion (the 18 or 24 bp insertion), and [TGATGG]_n_ at the most C-terminal insertion (the 6 bp insertion), suggesting that these repetitive sequences are highly prone to recombination (Fig. 8b, orange arrowheads). Because of these changes, the uMet′ codons of *tlh1*, *tlh2*, and *tlh4* located upstream of uMet are no longer in-frame to the long coding sequence from the dMet codons, and only short coding sequences are predicted because of a termination codon derived by a point mutation (Fig. 8b, light purple lines with a dark green line). Intriguingly, multiple nucleotide changes, including point mutations, insertions, and deletions, did not introduce premature termination codons within the long ORFs of the *tlh* genes in *972SD4*. Similarly, the *tlh* gene(s) of Ch3 in the *972* descendent strains (KYP33 and JP1225) lack 24 bp immediate downstream of the dMet codons; however, no frame shift occurs, and uMet′ codons are supplied by the SAS sequence in the same codon frame (Supplementary Fig. 1d).

### RNA expression from the *tlh* genes in *972SD4*

To examine our assumption that the *tlh* genes have longer protein-coding sequence than that previously predicted by PomBase, we determined ranges of *tlh* RNA expression by reverse transcription (RT)-PCR (Supplementary Fig. 10). We detected RNAs of *tlh* genes that contain at least 950 bases upstream of the dMet codon (~400 bp upstream of the uMet codon). These data suggest that the *tlh* genes may have protein-coding sequences that are potentially 543 bases longer than that defined in PomBase (Fig. 8b, a light purple line in the top panel).

To examine whether the *tlh* genes in *972SD4* carrying multiple mutations are capable to produce *dh* RNAs that participate in the formation of subtelomeric heterochromatin, we determined the RNA expression level from the *dh*-homologous region of each *tlh* gene in *clr4*∆ cells where the strong gene silencing by subtelomeric heterochromatin is omitted^23^. We found that these four *tlh* genes express *dh* RNAs; however, the expression levels were lower than one-fourth of that in the wild-type strain possibly due to lack of positive feedback regulation by other *tlh* RNA expression (Fig. 8c). These results suggest that DNAs of the *tlh* genes are prone to mutations without severely affecting RNA expression.

## Discussion

**Fig. 9:**
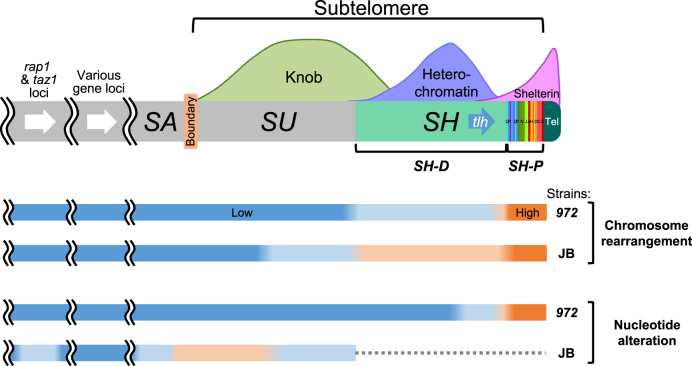
Summary of features of *S. pombe* subtelomeres. The *S. pombe SH* region is composed of two parts: the *SH-P* region with a mosaic of multiple segments, and the *SH-D* region with multiple blocks and boxes containing deletions and insertions. Colors of bars indicate relative rates of chromosome rearrangement or nucleotide alteration versus PomBase (orange, high rate; blue, low rate). The *SH-P* region shows high rates of chromosome rearrangement and nucleotide alteration even between the *972* strains (*972SD4* and JB22 vs. PomBase), whereas the *SH-D* region shows lower rates of these changes compared with those in the *SH-P* region. The *SU*, boundary, and *SA* regions and the *rap1* and *taz1* loci also exhibit medium levels of these changes in the JB strains that are phylogenetically distant from *972*. Thus, chromosomal regions around the subtelomeres (and the *rap1* and *taz1* loci) are prone to genome diversity. Dotted line indicates a region that was unable to be analyzed with the NGS data.

This study describes in-depth analyses of subtelomeres in *S. pombe*. We obtained complete sequences of subtelomeres in the standard *S. pombe* strain *972* by producing strains with single *SH* regions. We also extracted *SH* sequences of some natural isolates of *S. pombe* strains (JB strains) from previous NGS data. The whole sequences revealed that *SH* regions are composed of two parts: the telomere-adjacent *SH-P* region and telomere-distal *SH-D* region. The *SH-P* region is a mosaic of multiple common segments that vary among subtelomeres and strains, suggesting that this region is highly prone to chromosomal rearrangement during cell divisions. In contrast, the *SH-D* region shows high sequence similarity among subtelomeres and *972* strains, although there are some insertions, deletions, and chromosomal rearrangement, suggesting that the overall DNA structure of this region is stably maintained during short-term culturing. However, JB strains other than JB22 (*972*) exhibited striking variation in the structures of *SH-D* regions, indicating that *SH-D* regions are also susceptible to chromosomal rearrangement during long-term evolution of *S. pombe*. Interestingly, not only *SH* but also *SU* regions exhibit high rates of nucleotide changes among strains, whereas chromosomal regions outside of this region are subject to highly strict genome preservation. Thus, subtelomeres are hot spots for genome evolution and exhibit multiple patterns of genome variation (Fig. 9).

Human subtelomeres (*SH* regions in humans) are also mosaics of multiple common segments that correspond to the *SH-P* region in *S. pombe*. However, they contain no sequence equivalent to that of the *SH-D* region, i.e., a relatively long common sequence shared by all subtelomeres^9,19^. Common segments of the same categories are mostly nonidentical (~90–100% identities), and the location and copy number of each segment vary among individuals^9,40^. In *S. cerevisiae*, the subtelomeres have common X and Y′ elements, and ORFs of proteins such as PAU and FLO families; however, copy numbers of the Y′ element and the ORFs are highly variable among strains^18,41^. Based on these findings and studies in other species, along with our results in *S. pombe*, we propose that high variation in *SH* sequences is a common feature in eukaryotes.

What underlies this high variation of *SH* regions? First, DNA double-strand breaks (DSBs) are repaired by either HR or nonhomologous end joining. Vegetative cell cycle of the wild-type *S. pombe* strain lacks G_1_ phase because cells already possess sufficient mass to proceed to S phase when the previous mitosis is completed. Therefore, ~80% period of the *S. pombe* cell cycle is G_2_ phase, when HR predominates for DSB repair^42^. Because of the high sequence identities among *SH* regions, DNA repair by HR may occur frequently between *SH* regions of different chromosomes (interchromosomal repairs), as well as between sister chromosomes (intrachromosomal repairs), which causes gross rearrangement of chromosomes. Second, repetitive sequences within *SH* regions may be recognized by HR machineries, causing amplification or deletion of the repeat units. Third, repetitive sequences, including *S. pombe* telomeres and subtelomeres, are regions intrinsically difficult to replicate during S phase^43–45^. Replication fork collapse and erosion of telomeres and subtelomeres can result in formation of single-ended DNA breaks that are repaired by break-induced replication (BIR)^46^. Recent studies suggested that BIR is a highly inaccurate DNA repair mechanism, and causes high levels of mutations and chromosome rearrangements^47–52^. Therefore, BIR may cause high rates of mutations and chromosome rearrangements in *SH* regions. Fourth, the *SH-D* region serve as a fusion point of chromosome circularization when telomeres are lost^53^. *S. pombe* has only three chromosomes, which enables cells to survive telomere crisis by self-circularization of each chromosome^29^. Chromosome end fusions of Ch1 and Ch2 take place between H1–5 and their inverted sequences, H1′–5′, which are located in blocks I, II, and V (Fig. 5b, pink arrows)^53^. It is possible that chromosome circularization and re-linearization promote chromosome rearrangement of *SH-D* regions.

Importantly, in the subtelomeres (i.e., the *SH* and *SU* regions) of *S. pombe*, there is no gene essential for cell growth under normal culture conditions, and deletion of all *SH* regions does not affect cell growth per se^21^. Genomes of other species also contain multiple copies of the same genes in *SH* regions. This may explain why cells can continue to grow, even with mutations in *SH* (and *SU*) regions, resulting in the accumulation of mutations. The *SU* regions are known to form knob bodies that are highly condensed chromatin structures, which may prevent precise DNA replication or normal DNA repair, and causes the accumulation of nucleotide alterations^21,24^ (Fig. 9).

Surprisingly, the *tlh* genes in *972SD4* contain no nonsense mutation in their ORFs, although they contained various mutations compared with the *tlh2* gene in PomBase. We found that some of the mutations cause alterations in the amino acid sequences of the conserved RecQ motif (Supplementary Fig. 11). Absence of premature termination codon is important for suppression of mRNA degradation mediated by the NMD (nonsense-mediated mRNA decay) mechanism^54^. Thus, it is likely that the principal function of the *tlh* genes is to produce RNAs containing *dh* sequences, and that presence of the *tlh* ORFs is advantageous for their normal RNA expression, which induces heterochromatin formation. Although a previous study suggested that the *tlh* genes are involved in survival after telomere shortening^55^, functions of the Tlh protein is not clarified yet.

Over 70 years since Dr. Urs Leupold isolated *S. pombe* standard strains, *968* (*h*^90^), *972* (*h*^−^), and *975* (*h*^+^), from the Delft culture^26^, it has been believed that these three strains possess almost the same genetic information except for the mating-type genes. This and previous studies have shown that *972* has no *SH* sequence in Ch3, whereas some of its descendent strains possess *SH* region(s) with SAS in Ch3^21,27,28^ (Fig. 6 and Supplementary Fig. 1). It is noteworthy that the SAS sequence is absent from the genome of *972*. The three standard strains have been traveling over the world to produce numerous number of descendent strains through mating and meiosis^56^. Therefore, the assumption is that *968* and/or *975* had already had *SH* region(s) in Ch3 when they were first isolated.

Taken together, we propose that subtelomeres are highly polymorphic chromosomal regions and contribute to genome evolution. In this study, we have shown that the DNA sequence of the *tlh2* gene has changed from that of the original strain *972* after repeated rounds of cell division (Fig. 8b). Human *SH* regions also contain various genes, such as *DUX4* (associated with facioscapulohumeral muscular dystrophy) and olfactory receptor genes^9,19^. Thus, the high rates of polymorphisms in *SH* regions may contribute to human diversity and sometimes to disease susceptibilities. It is intriguing to investigate correlation between various human diseases and *SH* sequences. Overall, genome rearrangement, deletion, insertion, and mutation can cause changes in ORFs, which may result in diversification of species.

## Methods

### Strains and general techniques for *S. pombe*

*S. pombe* strains used in this study are listed in Supplementary Table 3. Growth media and basic genetic and biochemical techniques used in this study were described previously^57–59^.

### Construction of *972SD4* strains

To construct *972SD4* strains carrying single *SH* regions of the standard wild-type strain *972*, the *SD5* strain (ST3524)^21^, in which all five *SH* regions were replaced with selective marker genes (*his7*^+^ or *ura4*^+^), was crossed with strain *972*, and then the progeny were crossed back with the *SD5* strain (ST3479 or ST3524) again. The presence or absence of each *SH* region in the resulting progeny were examined by PFGE followed by Southern blotting.

### PFGE

PFGE of NotI-digested chromosomal DNA was performed using a CHEF-DR III Pulsed-Field Electrophoresis System (BioRad) under the following conditions: 1% SeaKem Gold Agarose (Lonza) in 0.5× TBE; temperature, 10 °C; initial switch time, 40 s; final switch time, 80 s; run time, 18 h; voltage gradient, 6.8 V/cm; and angle, 120°.

### Southern blotting

NotI-digested chromosomal DNA was separated by PFGE and subjected to Southern blotting. Telomeric DNA and TAS fragments (TAS1, TAS2, and TAS3) were excised from pNSU70^25^, and used as the telomere and TAS probes, respectively. The *SPBCPT2R1.03* ORF was amplified by PCR and used as the probe that specifically recognizes the *SH-D* regions of *subtel1L*, *subtel2L*, and *subtel2R*, but not *subtel1R* in *972*. These DNA fragments were labeled with digoxigenin (DIG), using a DIG High Prime DNA Labeling and Detection Starter Kit II (Roche), and signals were detected according to the manufacturer’s instructions.

### Cloning and sequencing of *SH* regions

DNA fragments containing *SH-P* regions (~5 kb) were amplified by PCR from genomic DNA of each *972SD4* strain using Phusion High-Fidelity DNA polymerase (Thermo Fisher) and the following primers.

$${\!\!\!\!}{\mathrm{jk}}1861\!:\!5^\prime \!- {\mathrm{ACTAGTGGATCCCCCTGTAACCACGTAACCTTGTAACC}} - 3^\prime$$

$${\mathrm{jk}}1862\!:\!5^\prime\! - {\mathrm{GAATTCCTGCAGCCCGGTTTGAGCATCTGTCAGAGGTAA}} - 3^\prime$$

Each DNA fragment was inserted at the Sma1 site of pBluescript SK(−) (Stratagene) using In-Fusion HD Cloning Kit (Clontech). The resulting plasmids were digested with Kpn1 and Xho1 at the multiple cloning site of the vector and treated with exonuclease III (Takara) and mung bean nuclease (Takara) for fixed times to obtain deletion series from the XhoI cutting site (5′-protruding end). Both ends of the plasmids were blunted with Klenow fragments (Takara) and ligated with DNA ligase (Takara). The re-circularized plasmids were cloned using *E. coli*, XL1-Blue (*recA1 endA1 gyrA96 thi-1 hsdR17 supE44 relA1 lac* [F′ *proAB lacI*^q^*Z*∆*M15* Tn*10* (Tet^r^)]), and then sequenced using the following primers that anneal to the vector.

$${\mathrm{st}}13\,\left( {{\mathrm{M}}13\,{\mathrm{primer}}\,{\mathrm{M}}3} \right){\!}:{\!}5^\prime \!- {\mathrm{GTAAAACGACGGCCAGT}} - 3^\prime$$

$${\mathrm{st}}14\,\left( {{\mathrm{M}}13\,{\mathrm{primer}}\,{\mathrm{RV}}} \right){\!}:{\!}5^\prime\! - {\mathrm{CAGGAAACAGCTATGAC}} - 3^\prime$$

The DNA sequence of *SH-P* was assembled using the overlapping sequences of serial deletion plasmids (Supplementary Fig. 2). Two independent strains of each *972SD4* were analyzed.

DNA fragments (1.3–2.9 kb) of the *SH-D* region were amplified by PCR and sequenced, except for the regions with some repeats that were sequenced using the deletion method described above. Two independent strains of each *972SD4* were analyzed.

DNA sequences were determined using BigDye Terminator v3.1 Cycle Sequencing Kit (Applied Biosystems), Prism 3130*xl* Genetic Analyzer (Applied Biosystems), and DNA Sequencing Analysis Software v5.4 (Applied Biosystems). In addition, some DNA sequences were determined by Eurofins Genomics Inc.

### Analyses of previous NGS data of JB strains

To analyze DNA sequences at subtelomeres and other chromosomal loci in JB strains, we utilized previous raw data from NGS by Tusso et al. (long-read sequencing by Nanopore MinION and PacBio RS II; NCBI Sequence Read Archive, PRJNA527756 [https://www.ncbi.nlm.nih.gov/bioproject/PRJNA527756])^30^ and those by Jeffares et al. (short-read sequencing by Illumina HiSeq 2000; European Nucleotide Archive, PRJEB2733 [https://www.ebi.ac.uk/ena/browser/view/PRJEB2733])^33^.

De novo assembly of the long reads (by MinION) were performed as follows. Adaptor and its adjacent (10–20 bp) sequences in raw read data were trimmed using Porechop (v0.2.4, https://github.com/rrwick/Porechop) and fastp (v0.20.1)^60^. The trimmed long reads were assembled by Canu 2.0 (ref. ^61^), using the NIG supercomputer. Chromosome configurations compared with those in strain *972* were analyzed using MUMmer4 (ref. ^62^).

To determine sequences at *SH* regions, we collected long reads that contain telomere repeats and telomere-adjacent segments at their ends using NCBI nucleotide BLAST (blastn). The collected reads were classified into several categories using AliView (v1.26)^63^ and MAFFT (v7.453)^64^, and the sequences of each category were combined into one consensus sequence using Minimap2 (v.2.17-r941)^65^ and Racon (v1.4.13)^66^. Locations of the *SH* sequences were determined by search for homologous sequences in the chromosome assembly described above. Long-read data (by PacBio RS II) were also used to improve the quality of sequences.

To determine sequences other than *SH*, we searched target sequences in the de novo assembly described above and polished them by Pilon (v1.23)^67^ using short-read data.

### RNA analyses

Total RNA was purified from exponentially growing cells as described previously^28^. For the RT-PCR, complementary DNA was synthesized with random primers using a High-Capacity cDNA Reverse Transcription Kit (Applied Biosystems) and analyzed by conventional PCR (Supplementary Fig. 10) or quantitative PCR using a StepOne Real-Time PCR System (Fig. 8c). Primer sequences are listed in Supplementary Table 4.

### Reporting summary

Further information on research design is available in the Nature Research Reporting Summary linked to this article.

## Supplementary information

Supplementary information

Reporting Summary

## Data Availability

DNA sequences of newly sequenced *SH* regions are available in the DNA Data Bank of Japan (DDBJ) under accession codes LC521649 (~1.7 kb of the *SH-D* region [*SH1L*] in *972SD4[1L*+*]* #1), LC521650 (~1.7 kb of the *SH-D* region [*SH1L*] in *972SD4[1**L*+*]* #2), LC521651 (the *SH1L* region in *972SD4[1**L*+*]* #1), LC521652 (the *SH1L* region in *972SD4[1**L*+*]* #2), LC521653 (the *SH1R* region in *972SD4[1**R*+*]* #1), LC521654 (the *SH1R* region in *972SD4[1**R*+*]* #2), LC521655 (the *SH2L* region in *972SD4[2**L*+*]* #1), LC521656 (the *SH2L* region in *972SD4[2**L*+*]* #2), LC521657 (the *SH2R* region in *972SD4[2**R*+*]* #1), and LC521658 (the *SH2R* region in *972SD4[2**R*+*]* #2). Figures 3, 5a, b, 6a, 7, and 8a, b, Supplementary Figs. 3, 6, 7, 9, and 11, and Supplementary Table 2 are associated with these sequence data. Source data are provided with this paper.

## Source data

Source Data

## References

[1] Goffeau A (1997). The yeast genome directory. Nature.

[2] Wood V (2002). The genome sequence of *Schizosaccharomyces pombe*. Nature.

[3] *C. elegans* Sequencing Consortium. Genome sequence of the nematode *C. elegans*: a platform for investigating biology. *Science* **282**, 2012–2018 (1998). 10.1126/science.282.5396.2012 9851916

[4] Adams MD (2000). The genome sequence of *Drosophila melanogaster*. Science.

[5] The *Arabidopsis* Genome Initiative. Analysis of the genome sequence of the flowering plant *Arabidopsis thaliana*. *Nature* **408**, 796–815 (2000). 10.1038/35048692 11130711

[6] Lander ES (2001). Initial sequencing and analysis of the human genome. Nature.

[7] Venter JC (2001). The sequence of the human genome. Science.

[8] International Human Genome Sequencing Consortium. (2004). Finishing the euchromatic sequence of the human genome. Nature.

[9] Linardopoulou EV (2005). Human subtelomeres are hot spots of interchromosomal recombination and segmental duplication. Nature.

[10] Arlt MF, Durkin SG, Ragland RL, Glover TW (2006). Common fragile sites as targets for chromosome rearrangements. DNA Repair.

[11] Kurosawa K, Ohta K (2011). Genetic diversification by somatic gene conversion. Genes.

[12] Almeida H, Godinho Ferreira M (2013). Spontaneous telomere to telomere fusions occur in unperturbed fission yeast cells. Nucleic Acids Res..

[13] Chen NWG (2018). Common bean subtelomeres are hot spots of recombination and favor resistance gene evolution. Front. Plant Sci..

[14] Chikashige Y (1994). Telomere-led premeiotic chromosome movement in fission yeast. Science.

[15] Miyoshi T, Kanoh J, Saito M, Ishikawa F (2008). Fission yeast Pot1-Tpp1 protects telomeres and regulates telomere length. Science.

[16] de Lange T (2009). How telomeres solve the end-protection problem. Science.

[17] Fujita I (2012). Telomere-nuclear envelope dissociation promoted by Rap1 phosphorylation ensures faithful chromosome segregation. Curr. Biol..

[18] Louis EJ (1995). The chromosome ends of *Saccharomyces cerevisiae*. Yeast.

[19] Stong N (2014). Subtelomeric CTCF and cohesin binding site organization using improved subtelomere assemblies and a novel annotation pipeline. Genome Res..

[20] Stadler G (2013). Telomere position effect regulates DUX4 in human facioscapulohumeral muscular dystrophy. Nat. Struct. Mol. Biol..

[21] Tashiro S, Nishihara Y, Kugou K, Ohta K, Kanoh J (2017). Subtelomeres constitute a safeguard for gene expression and chromosome homeostasis. Nucleic Acids Res..

[22] Cam HP (2005). Comprehensive analysis of heterochromatin- and RNAi-mediated epigenetic control of the fission yeast genome. Nat. Genet..

[23] Kanoh J, Sadaie M, Urano T, Ishikawa F (2005). Telomere binding protein Taz1 establishes Swi6 heterochromatin independently of RNAi at telomeres. Curr. Biol..

[24] Matsuda A (2015). Highly condensed chromatins are formed adjacent to subtelomeric and decondensed silent chromatin in fission yeast. Nat. Commun..

[25] Sugawara, N. F. DNA sequences at the telomeres of the fission yeast *S. pombe*. Ph.D. Thesis, Harvard University (1988).

[26] Leupold U (1950). Die vererbung von homothallie und heterothallie bei *Schizosaccharomyces pombe*. C. R. Trav. Lab. Carlsberg Ser. Physiol..

[27] Ohno Y, Ogiyama Y, Kubota Y, Kubo T, Ishii K (2016). Acentric chromosome ends are prone to fusion with functional chromosome ends through a homology-directed rearrangement. Nucleic Acids Res..

[28] Tashiro S (2016). Shugoshin forms a specialized chromatin domain at subtelomeres that regulates transcription and replication timing. Nat. Commun..

[29] Nakamura TM, Cooper JP, Cech TR (1998). Two modes of survival of fission yeast without telomerase. Science.

[30] Tusso S (2019). Ancestral admixture is the main determinant of global biodiversity in fission yeast. Mol. Biol. Evol..

[31] Bochman ML, Paeschke K, Zakian VA (2012). DNA secondary structures: stability and function of G-quadruplex structures. Nat. Rev. Genet..

[32] Chaudari A, Huberman JA (2012). Identification of two telomere-proximal fission yeast DNA replication origins constrained by nearby cis-acting sequences to replicate in late S phase. F1000Res..

[33] Jeffares DC (2015). The genomic and phenotypic diversity of *Schizosaccharomyces pombe*. Nat. Genet..

[34] Fujita I, Tanaka M, Kanoh J (2012). Identification of the functional domains of the telomere protein Rap1 in *Schizosaccharomyces pombe*. PLoS ONE.

[35] Cooper, J. P., Nimmo, E. R., Allshire, R. C. & Cech, T. R. Regulation of telomere length and function by a Myb-domain protein in fission yeast. *Nature* **385**, 744–747 (1997). 10.1038/385744a0 9034194

[36] Kanoh J, Ishikawa F (2001). spRap1 and spRif1, recruited to telomeres by Taz1, are essential for telomere function in fission yeast. Curr. Biol..

[37] Chen Y (2011). A conserved motif within RAP1 has diversified roles in telomere protection and regulation in different organisms. Nat. Struct. Mol. Biol..

[38] Deng W (2015). Fission yeast telomere-binding protein Taz1 is a functional but not a structural counterpart of human TRF1 and TRF2. Cell Res..

[39] Kozak M (1987). An analysis of 5’-noncoding sequences from 699 vertebrate messenger RNAs. Nucleic Acids Res..

[40] Young E, Abid HZ, Kwok PY, Riethman H, Xiao M (2020). Comprehensive analysis of human subtelomeres by whole genome mapping. PLoS Genet..

[41] Yue JX (2017). Contrasting evolutionary genome dynamics between domesticated and wild yeasts. Nat. Genet..

[42] Ferreira MG, Cooper JP (2004). Two modes of DNA double-strand break repair are reciprocally regulated through the fission yeast cell cycle. Genes Dev..

[43] Miller KM, Rog O, Cooper JP (2006). Semi-conservative DNA replication through telomeres requires Taz1. Nature.

[44] Takikawa M, Tarumoto Y, Ishikawa F (2017). Fission yeast Stn1 is crucial for semi-conservative replication at telomeres and subtelomeres. Nucleic Acids Res..

[45] Ivanova T (2020). Budding yeast complete DNA synthesis after chromosome segregation begins. Nat. Commun..

[46] Kramara J, Osia B, Malkova A (2018). Break-induced replication: the where, the why, and the how. Trends Genet..

[47] Smith CE, Llorente B, Symington LS (2007). Template switching during break-induced replication. Nature.

[48] Deem A (2011). Break-induced replication is highly inaccurate. PLoS Biol..

[49] Pardo B, Aguilera A (2012). Complex chromosomal rearrangements mediated by break-induced replication involve structure-selective endonucleases. PLoS Genet..

[50] Anand RP (2014). Chromosome rearrangements via template switching between diverged repeated sequences. Genes Dev..

[51] Sakofsky CJ (2014). Break-induced replication is a source of mutation clusters underlying kataegis. Cell Rep..

[52] Sakofsky CJ (2015). Translesion polymerases drive microhomology-mediated break-induced replication leading to complex chromosomal rearrangements. Mol. Cell.

[53] Wang X, Baumann P (2008). Chromosome fusions following telomere loss are mediated by single-strand annealing. Mol. Cell.

[54] Behm-Ansmant I (2007). mRNA quality control: an ancient machinery recognizes and degrades mRNAs with nonsense codons. FEBS Lett..

[55] Mandell JG, Goodrich KJ, Bahler J, Cech TR (2005). Expression of a RecQ helicase homolog affects progression through crisis in fission yeast lacking telomerase. J. Biol. Chem..

[56] Fantes PA, Hoffman CS (2016). A Brief history of *Schizosaccharomyces pombe* research: a perspective over the past 70 years. Genetics.

[57] Bahler J (1998). Heterologous modules for efficient and versatile PCR-based gene targeting in *Schizosaccharomyces pombe*. Yeast.

[58] Forsburg SL, Rhind N (2006). Basic methods for fission yeast. Yeast.

[59] Moreno S, Klar A, Nurse P (1991). Molecular genetic analysis of fission yeast *Schizosaccharomyces pombe*. Methods Enzymol..

[60] Chen S, Zhou Y, Chen Y, Gu J (2018). fastp: an ultra-fast all-in-one FASTQ preprocessor. Bioinformatics.

[61] Koren S (2017). Canu: scalable and accurate long-read assembly via adaptive k-mer weighting and repeat separation. Genome Res..

[62] Marcais G (2018). MUMmer4: A fast and versatile genome alignment system. PLoS Comput. Biol..

[63] Larsson A (2014). AliView: a fast and lightweight alignment viewer and editor for large datasets. Bioinformatics.

[64] Katoh K, Misawa K, Kuma K, Miyata T (2002). MAFFT: a novel method for rapid multiple sequence alignment based on fast Fourier transform. Nucleic Acids Res..

[65] Li H (2018). Minimap2: pairwise alignment for nucleotide sequences. Bioinformatics.

[66] Vaser R, Sovic I, Nagarajan N, Sikic M (2017). Fast and accurate *de novo* genome assembly from long uncorrected reads. Genome Res..

[67] Walker BJ (2014). Pilon: an integrated tool for comprehensive microbial variant detection and genome assembly improvement. PLoS ONE.

