# Supplementary material for: Complete sequences of Schizosaccharomyces pombe subtelomeres reveal multiple patterns of genome variation
Source: Nat Commun. 2021 Jan 27;12:611. doi: 10.1038/s41467-020-20595-1 (PMC7840980; doi:10.1038/s41467-020-20595-1)
Supplement: Supplementary file 1 — Supplementary information [file 41467_2020_20595_MOESM1_ESM.pdf]

Supplementary information

for

Complete sequences of *Schizosaccharomyces pombe* subtelomeres reveal multiple patterns of genome variation

Yusuke Oizumi[1,2], Takuto Kaji[2], Sanki Tashiro[2,3], Yumiko Takeshita[2],

Yuko Date[1], and Junko Kanoh[1,2,4]*

[1] Department of Life Sciences, Graduate School of Arts and Sciences, the University of Tokyo, 3-8-1 Komaba, Meguro-ku, Tokyo 153-8902, Japan

[2] Former address: Institute for Protein Research, Osaka University, 3-2 Yamadaoka, Suita, Osaka 565-0871, Japan

[3] Present address: Institute of Molecular Biology, University of Oregon, 1370 Franklin Blvd, Eugene, OR, USA

[4] Department of Biological Sciences, Graduate School of Science, the University of Tokyo, 7-3-1 Hongo, Bunkyo-ku, Tokyo 113-0033, Japan

* Correspondence: jkanoh@bio.c.u-tokyo.ac.jp

Contents: Supplementary Figures 1-11

Supplementary Tables 1–4

Supplementary References

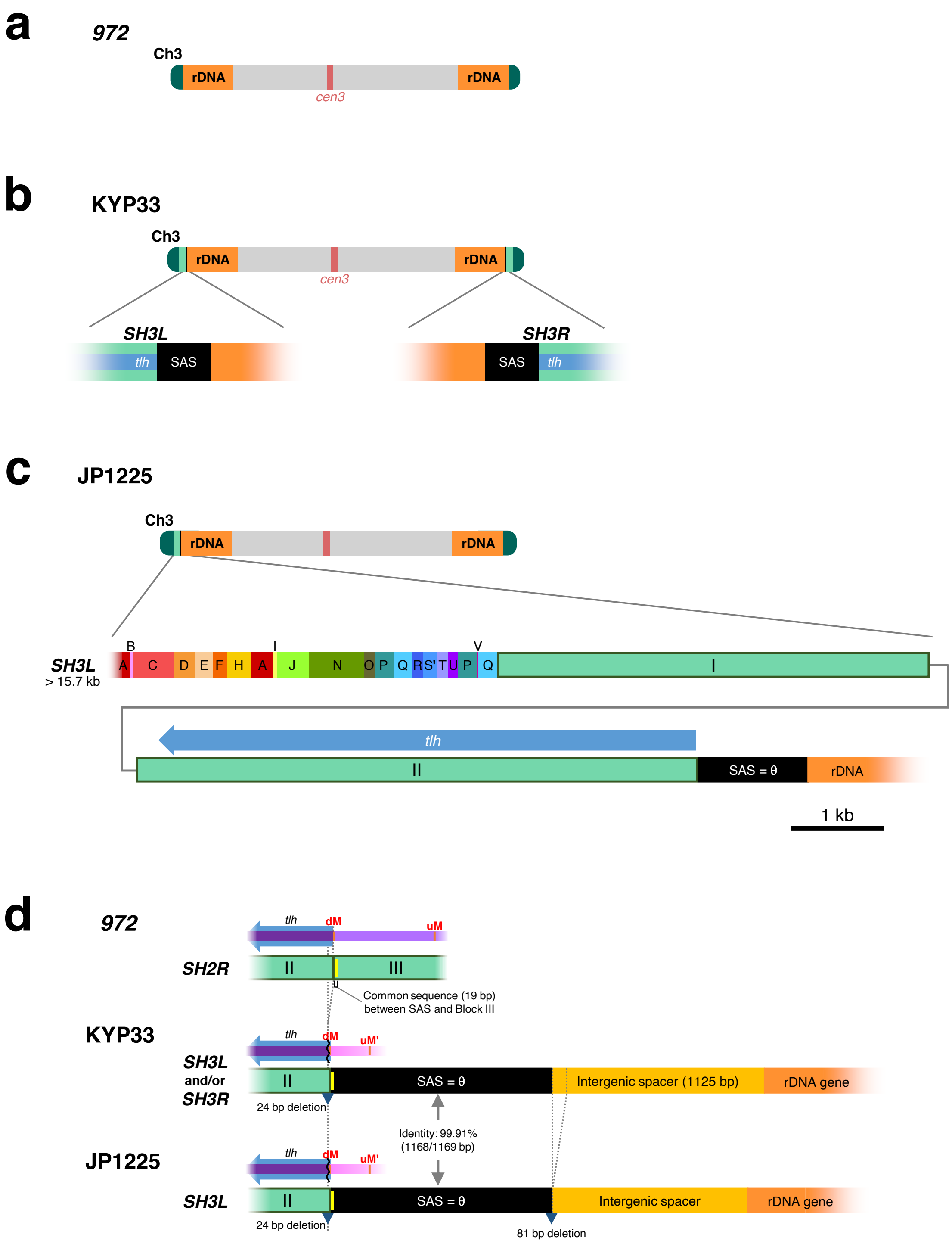

Supplementary Fig. 1 Structures of chromosome ends of Ch3 in descendent strains of *972*.

(a) Strain *972* has no subtelomere sequence in Ch3.

(b) Strain KYP33 (*h⁻ leu1-32 ura4-D18*) has subtelomeres including *SH* and SAS (subtelomere-associated sequence) sequences at both ends of Ch3[1].

(c) Strain JP1225 (*h⁻ ade6-M216 leu1-32 ura4-D18 his7-366*) has a subtelomere including *SH-P* (homologous with pNSU71, see Fig. 3a), blocks I and II, and SAS at the left arm of Ch3[2].

(d) Detailed information for the Ch3 ends in the three strains. The sequence of SAS which has not been found in PomBase-*972*, is the same as that of box θ (see Fig. 6). Yellow box, common sequence (19 bp) between SAS and block III; blue arrow with a purple line, homologous region with the *tlh2* ORF defined in PomBase; pale purple line, predicted extension of the coding region of the *tlh2* gene as described in Fig. 8b; pink line, predicted in-frame extension of the coding region in the same reading frame; blue arrowhead, nucleotide deletion; dM (downstream M), the original first methionine codon of the *tlh2* ORF in PomBase-*972* or its corresponding first methionine codon in KYP33 and JP1225; uM (upstream M) and uM′, putative first methionine codons upstream of dM (also see the main text and Fig. 8b). Note that the 24 bp-deletions just downstream of dM codon in KYP33 and JP1225 do not cause frame shifts or premature nonsense mutation.

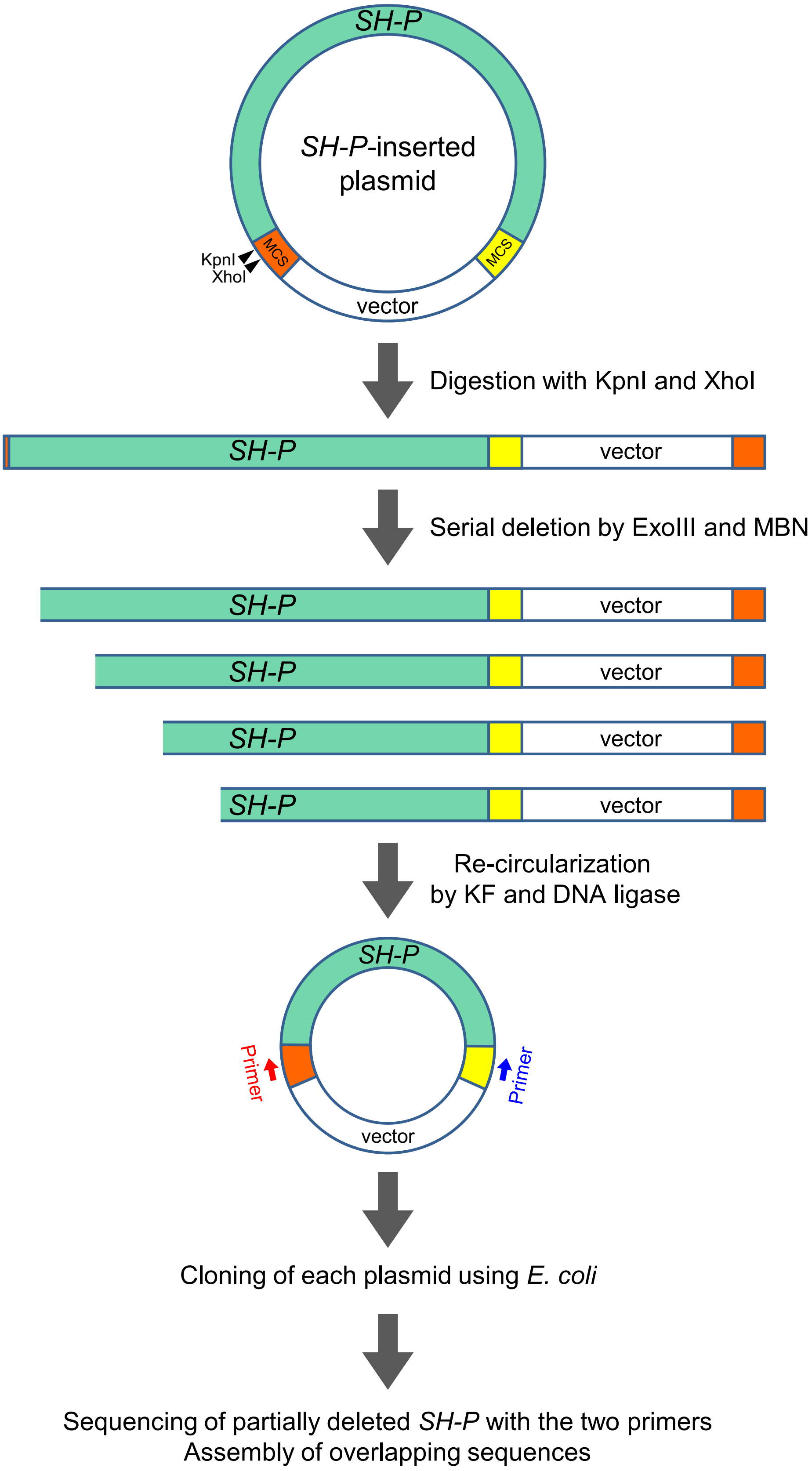
SH-P
SH-P-inserted plasmid
KpnI
XhoI
MCS
MCS
vector
Digestion with KpnI and XhoI
SH-P
vector
Serial deletion by ExoIII and MBN
SH-P
vector
SH-P
vector
SH-P
vector
SH-P
vector
Re-circularization
by KF and DNA ligase
SH-P
Primer
Primer
vector
Cloning of each plasmid using E. coli
Sequencing of partially deleted SH-P with the two primers
Assembly of overlapping sequences

Supplementary Fig. 2 Sequencing of *SH-P* regions by the serial deletion method.

The *SH-P* region with various common segments (~5 kb) was amplified by PCR from a *972SD4* strain, and inserted into a vector. The resultant plasmid was digested with restriction enzymes, KpnI and XhoI, at the multi-cloning sites (MCSs) of the vector. The linearized plasmid was serially deleted from the 5′-overhang at XhoI site by treating with exonuclease III (ExoIII) and mung bean nuclease (MBN) for fixed times. After deletion of the *SH-P* DNA, both ends of the plasmid were blunted by klenow fragment (KF) and ligated by DNA ligase, and then re-circularized plasmids were cloned using *Escherichia coli* (*E. coli*). The partially deleted *SH-P* DNAs were sequenced using primers that anneal to MCSs of the vector. The sequence reads were assembled using overlapping sequences.

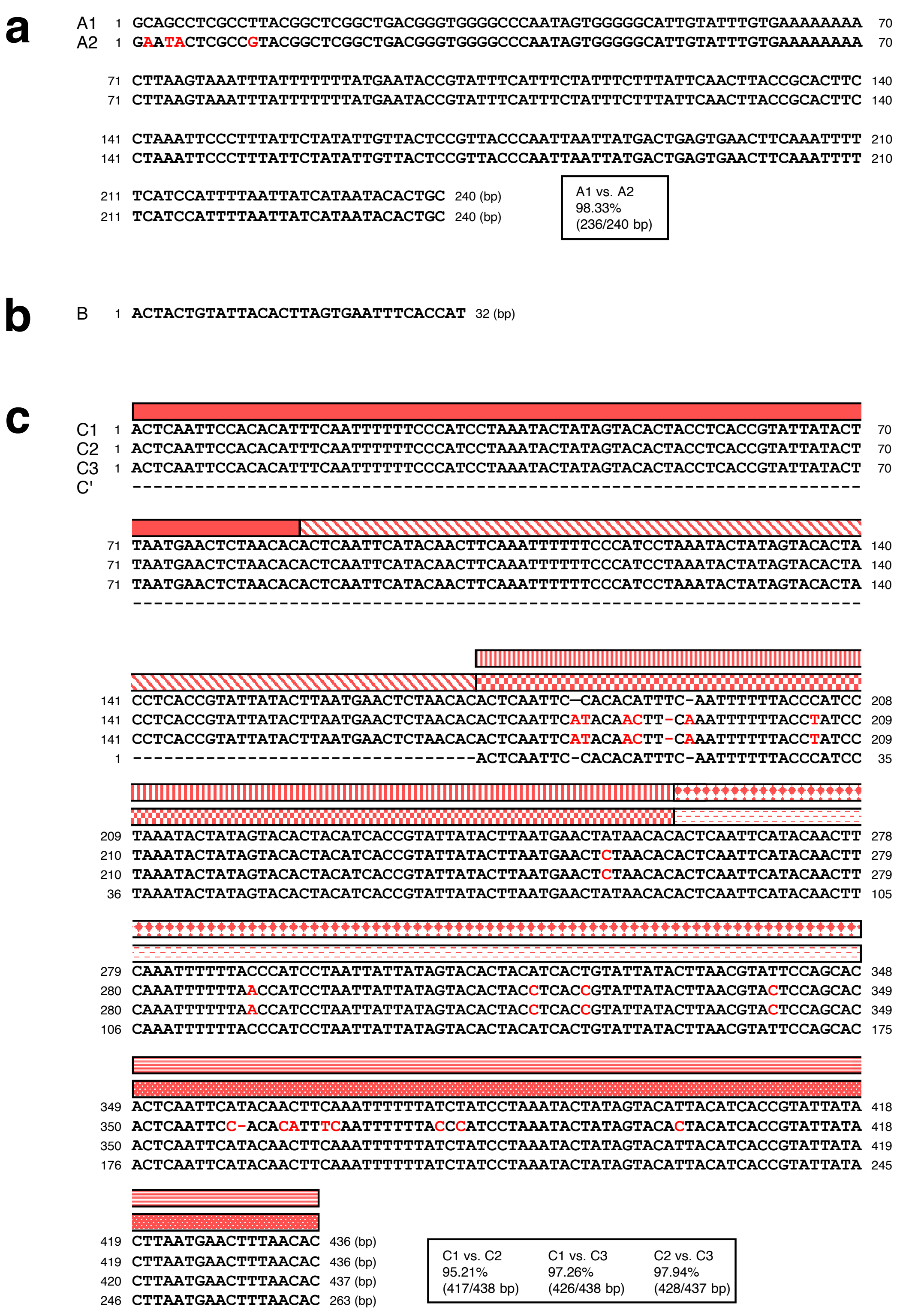
a
A1 1 GCAGCCTCGCCTTACGGCTCGGCTGACGGGTGGGGCCCAATAGTGGGGGCATTGTATTTGTGAAAAAAAA 70
A2 1 GAATACTCGCCGTACGGCTCGGCTGACGGGTGGGGCCCAATAGTGGGGGCATTGTATTTGTGAAAAAAAA 70
71 CTTAAGTAAATTTATTTTTTTATGAATACCGTATTTCATTTCTATTTCTTTATTCAACTTACCGCACTTC 140
71 CTTAAGTAAATTTATTTTTTTATGAATACCGTATTTCATTTCTATTTCTTTATTCAACTTACCGCACTTC 140
141 CTAAATTCCCTTTATTCTATATTGTTACTCCGTTACCCAATTAATTATGACTGAGTGAACTTCAAATTTT 210
141 CTAAATTCCCTTTATTCTATATTGTTACTCCGTTACCCAATTAATTATGACTGAGTGAACTTCAAATTTT 210
211 TCATCCATTTTAATTATCATAATACACTGC 240 (bp)
211 TCATCCATTTTAATTATCATAATACACTGC 240 (bp)
A1 vs. A2
98.33%
(236/240 bp)
b
B 1 ACTACTGTATTACACTTAGTGAATTTCACCAT 32 (bp)
c
C1 1 ACTCAATTCCACACATTTCAATTTTTTCCCATCCTAAATACTATAGTACACTACCTCACCGTATTATACT 70
C2 1 ACTCAATTCCACACATTTCAATTTTTTCCCATCCTAAATACTATAGTACACTACCTCACCGTATTATACT 70
C3 1 ACTCAATTCCACACATTTCAATTTTTTCCCATCCTAAATACTATAGTACACTACCTCACCGTATTATACT 70
C' --
71 TAATGAACTCTAACACACTCAATTCATACAACTTCAAATTTTTTCCCATCCTAAATACTATAGTACACTA 140
71 TAATGAACTCTAACACACTCAATTCATACAACTTCAAATTTTTTCCCATCCTAAATACTATAGTACACTA 140
71 TAATGAACTCTAACACACTCAATTCATACAACTTCAAATTTTTTCCCATCCTAAATACTATAGTACACTA 140
----
141 CCTCACCGTATTATACTTAATGAACTCTAACACACTCAATTC-CACACATTTC-AATTTTTTACCCATCC 208
141 CCTCACCGTATTATACTTAATGAACTCTAACACACTCAATTCATACAACTT-CAAATTTTTTACCTATCC 209
141 CCTCACCGTATTATACTTAATGAACTCTAACACACTCAATTCATACAACTT-CAAATTTTTTACCTATCC 209
1 ---------------------------------ACTCAATTC-CACACATTTC-AATTTTTTACCCATCC 35
209 TAAATACTATAGTACACTACATCACCGTATTATACTTAATGAACTATAACACACTCAATTCATACAACTT 278
210 TAAATACTATAGTACACTACATCACCGTATTATACTTAATGAACTCTAACACACTCAATTCATACAACTT 279
210 TAAATACTATAGTACACTACATCACCGTATTATACTTAATGAACTCTAACACACTCAATTCATACAACTT 279
36 TAAATACTATAGTACACTACATCACCGTATTATACTTAATGAACTATAACACACTCAATTCATACAACTT 105
279 CAAATTTTTTACCCATCCTAATTATTATAGTACACTACATCACTGTATTATACTTAACGTATTCCAGCAC 348
280 CAAATTTTTTAACCATCCTAATTATTATAGTACACTACCTCACCGTATTATACTTAACGTACTCCAGCAC 349
280 CAAATTTTTTAACCATCCTAATTATTATAGTACACTACCTCACCGTATTATACTTAACGTACTCCAGCAC 349
106 CAAATTTTTTACCCATCCTAATTATTATAGTACACTACATCACTGTATTATACTTAACGTATTCCAGCAC 175
349 ACTCAATTCATACAACTTCAAATTTTTTATCTATCCTAAATACTATAGTACATTACATCACCGTATTATA 418
350 ACTCAATTCC-ACACATTTCAATTTTTTACCCATCCTAAATACTATAGTACACTACATCACCGTATTATA 418
350 ACTCAATTCATACAACTTCAAATTTTTTATCTATCCTAAATACTATAGTACATTACATCACCGTATTATA 419
176 ACTCAATTCATACAACTTCAAATTTTTTATCTATCCTAAATACTATAGTACATTACATCACCGTATTATA 245
419 CTTAATGAACTTTAACAC 436 (bp)
419 CTTAATGAACTTTAACAC 436 (bp)
420 CTTAATGAACTTTAACAC 437 (bp)
246 CTTAATGAACTTTAACAC 263 (bp)
C1 vs. C2
95.21%
(417/438 bp)
C1 vs. C3
97.26%
(426/438 bp)
C2 vs. C3
97.94%
(428/437 bp)

c*

```
c1  1 ACTCAATTCC-ACACATTTCAA-TTTTTT-CCCATCCTAAATACTATAGTACACTACCTCACCGTATTAT 67
c2  1 ACTCAATTCATACAACTT-CAAATTTTTT-CCCATCCTAAATACTATAGTACACTACCTCACCGTATTAT 68
c3  1 ACTCAATTCC-ACACATTTCAA-TTTTTTACCCATCCTAAATACTATAGTACACTACATCACCGTATTAT 68
c4  1 ACTCAATTCATACAACTT-CAAATTTTTTACCCATCCTAATTATTATAGTACACTACATCACTGTATTAT 69
c5  1 ACTCAATTCATACAACTT-CAAATTTTTTATCTATCCTAAATACTATAGTACATTACATCACCGTATTAT 69
c6  1 ACTCAATTCATACAACTT-CAAATTTTTTACCTATCCTAAATACTATAGTACACTACATCACCGTATTAT 69
c7  1 ACTCAATTCATACAACTT-CAAATTTTTTAACCATCCTAATTATTATAGTACACTACCTCACCGTATTAT 69
c8  1 ACTCAATTCC-ACACATTTCAA-TTTTTTACCCATCCTAAATACTATAGTACACTACATCACCGTATTAT 68

   68 ACTTAATGAACTCTAACAC 86 (bp)
   69 ACTTAATGAACTCTAACAC 87 (bp)
   69 ACTTAATGAACTATAACAC 87 (bp)
   70 ACTTAACGTATTCCAGCAC 88 (bp)
   70 ACTTAATGAACTTTAACAC 88 (bp)
   70 ACTTAATGAACTCTAACAC 88 (bp)
   70 ACTTAACGTACTCCAGCAC 88 (bp)
   69 ACTTAATGAACTTTAACAC 87 (bp)
```

	c2		c3		c4		c5		c6		c7		c8	
	Match /Total(bp)	Identity(%)	Match /Total(bp)	Identity(%)	Match /Total(bp)	Identity(%)	Match /Total(bp)	Identity(%)	Match /Total(bp)	Identity(%)	Match /Total(bp)	Identity(%)	Match /Total(bp)	Identity(%)
c1	82/88	93.18	84/87	96.55	73/89	82.02	77/89	86.52	80/89	89.89	75/89	84.27	84/87	96.55
c2			80/89	89.89	78/88	88.64	82/88	93.18	85/88	96.59	80/88	90.91	80/89	89.89
c3					74/89	83.15	79/89	88.76	81/89	91.01	74/89	83.15	86/87	98.85
c4							76/88	86.36	79/88	89.77	84/88	95.45	74/89	83.15
c5									85/88	96.59	77/88	87.50	80/89	89.89
c6											79/88	89.77	81/89	91.01
c7													74/89	83.15

d

```
D1    1 ACTCAGTTCAAATCAACTTCAAATTTTTCATCCGTTTTATTCACCCATGCACACTACACCACCGTATTAC 70
D2    1 ACTCAGTTCAAATCAACTTCAAATTTTTCATCCGTTTTATTCACCCATACACACTACACCACCTTATTAC 70
D3    1 ACTCAGTTCAAATCAACTTCAAATTTTTCATCCGTTTTATTCACCCATACACACTACACCACCTTATTAC 70

     71 AATTAGTGAATTCCAACATAGTCTAATTCCATGAACTTCAACTTTTTGTCCATCTTCACTATTGCACTAC 140
     71 AATTAGTGAATTCCAACATAGTCTAATTCCATGAACTTCAACTTTTTGTCCATCTTCACTATTGCACTAC 140
     71 AATTAGTGAATTCCAACATAGTCTAATTCCATGAACTTCAACTTTTTGTCCATCTTCACTATTGCACTAC 140

    141 CCTACATCCTCGTATTCTACTTAATCTACTCCAACACCTTTTAATCCATTCATCTTCTTTTCTTTTACTC 210
    141 CCTACATCCTCGTATTCTACTTAATCTACTCCAACACCTTTTAATCCATTCATCTTCTTTTCTTTTACTC 210
    141 CCTACATCCTCGTATTCTACTTAATCTACTCCAACACCTTTTAATCCATTCATCTTCTTTTCTTTTACTC 210

    211 TTTTATTTTATTTATTTTATT 231 (bp)
    211 TTTTATTTTATTTATTCTATT 231 (bp)
    211 TTTTATTTTATTTATTTTATT 231 (bp)
```

D1 vs. D2	D1 vs. D3	D2 vs. D3
98.70%	99.13%	99.57%
(228/231 bp)	(229/231 bp)	(230/231 bp)

e

```
E     1 ---------------------------------------------------------------TATTCTA 7
E'    1 ------------------------------------TATTCTATTTATTCTATT----CTATTTATTC-- 28
E''   1 TATTCTATTTATTCTATTTATTCTATTTATTCTATTTATTCTATTTATTCTATTTATTCTATTTATTCTA 70

      8 TTTATTCTATTTATTCTATTCCATTACTTTCTTCCTCTTCTTTCTTCCATAC------------------ 59
     29 --TATTCTATTTATTCTATTCCATTACTTTCTTCCTCTTCTTTCTTCCATACTCTATCCTACCTACTCTC 96
     71 TTTATTCTATTTATTCTATTCCATTACTTTCTTCCTCTTCTTTCTTCCATACTCTATCCTACCTACTCTC 140

     60 --------------------CCTACCTACTCTACCTATTCTC-----------------CTCCTTCATTT 92
     97 TACCTACTCTACCTACTCTACCTACCTACTCTACCTATTCTCCTCCTTCATTTCCTTCTCTCCTTCATTT 166
    141 TACCTACTCTACCTACTCTACCTACCTACTCTACCTATTCTC---------------------------- 182

     93 CCTTCTCTCCTTCATTTCCTTCT 115 (bp)
    167 CCTTCTCTCCTTCATTTCCTTCT 189 (bp)
    183 ------CTCCTTCATTTCCTTCT 199 (bp)
```

f

```
F1   1 CTCTCTCTAAAAACGTTTAACGAATACTCGCCTTACGGCTCGGCTGACGGGTGGGGG-CCAATAGTGGGG  69
F2   1 CTCTCTCTAAAAACGTTTAACGAATACTCGCCTTACGGCTCGGCTGACGGGTGGGGC-CCAATAGTGGGG  69
F3   1 CTCTCTCTAAAAACGTTTAACGAATACTCGCCTTACGGCTCGGCTGACGGGTGGGGGTCCAGTAGTGGGG  70

    70 GCATTTTATATATGTGAAAAAAACTTAAGTAAATTTAAATTTTTATAAATAACGCAACTTAATTATCTTT 139
    70 GCA--TTGTATATGTGAAAAAAACTTAAGTAAATTTAAATTTTTATAAATAACGCAGCTTAATTATCTTT 137
    71 GCA--TTGTATATGTGAAAAAAACTTAAGTAAATTTAAATTTTTATAAATAACGCAGCTTAATTATCTTT 138

   140 CTTTATCT 147 (bp)
   138 CTTTATCT 145 (bp)
   139 CTTTATCT 146 (bp)
```

F1 vs. F2	F1 vs. F3	F2 vs. F3
96.60%	95.95%	97.95%
(142/147 bp)	(142/148 bp)	(143/146 bp)

g

```
G    1 ATATTACCGTACGTTCTAATTGCTCCTTATTCTACATTGTTACTTTGTAACCCAATTAATTATGACTGAG  70
    71 TGAACTTCAAATTTTTCATCCATTACCATTACCATATTACGCTGCACTACAGTATTACACTTAGTGAATT 140
   141 TCAACAT 147 (bp)
```

h

```
H1   1 TCATTATCATACTACGCTGCACAACTGTAATCTACTTAATCTACTCCAATATAGTCCTCTGCTTTTATCT  70
H2   1 TCATTATCATACTACGCTGCACAACTATAATCTACTTAATGTACTCCAATATAGTCCTCTGCTTTTATCT  70

    71 TCTTTCCTCTTACTTTACTTATTTCTTTTACTCTCTTACTTTATTTATTTACTCTCCTTACTCTGTTTCT 140
    71 TCTTTCCTCTTACTTTACTTATTTCTTTTACTCTCTTACTTTATTTATTTACTCTCCTTACTCTGTTTCT 140

   141 TCTATTCTATTCATCTTACTTCTTCCTCATTTCTTCTCTCTTCTCTTCTATATCTTCTACTCTTTCATAC 210
   141 TCTATTCTATTCATCTTACTTCTTCCTCATTTCTTCTCTCTTCTCTTCTATATCTTCTACTCTTTCATAC 210

   211 CTCTCTTACCTCCATCCATTATCCATTCTCTCTCTCCAAAATCGTTTAAC 260 (bp)
   211 CTCTCTTACCTCCATCCATTATCCATTCTCTCTCTCCAAAATCGTTTAAC 260 (bp)
```

H1 vs. H2
99.23%
(258/260 bp)

i

```
I    1 GCCCCTTTATTCTACTTACTGCACCCTAACGC 32 (bp)
```

j

```
J    1 ACTCAAGCCCACACATTTCAACTTTTTATTCACCATATCTCATTATATCTCCTAATCATACATAATGAAT  70
    71 CCTACCACGCTCAATTCAACCCAACTTCATTTTCTTCATTCATTTTCATTACCATAGTACGCTCCACAAC 140
   141 TGTAATCTACTTAATGTACTCCAACATACTCAACTTCCACACATTTCAAAATTTTTTTACCCATTTTAT 210
   211 TTACCCCTATTATACTTACCGTACTCCAACACACTCAATACATACCAACTTCAAATTTTTTATGCATCTG 280
   281 ACTTTCCAATATACACTATGTATCCATCCTTATTTACTTTACTTTCTAATACTCTCTTACTT 342 (bp)
```

k

```
K    1 TAATTACTTCTTTATTCATCTTATTCTATTTATTTTATTTATTCCTTACTCCCTACTCCCTACTCCCTAC  70
K'   1 TAATTACTTCTTTATTCATCTTATTCTATTTATTTTATTTATTCCT-------TACTCCCTACTCCCTAC  63

    71 TCCCTACTCTCTACTCTATTCTGTATTCTATATTCTATATTCTATATTCTATATTCTATATTCTATATTC 140
    64 TCCCTACTCTCTACTCTATTCTG--------------TATTCTATATTCTATATTCTATATTCTATATTC 119

   141 TATATTCTATATTCTATATTTTCTTTTACCTCCTTTATCTTTCGTTAACATGTTTAACGAATACTCGCTT 210
   120 TATATTCTATATTCTATATTTTCTTTTACCTCCTTTATCTTTCGTTAACATGTTTAACGAATACTCGCTT 189

   211 TACAGCTCGTTACCCACCTCACACATCACTGACTCAACTTCAAATTTCTCAACCGTACTCATTACCATAG 280
   190 TACAGCTCGTTACCCACCTCACACATCACTGACTCAACTTCAAATTTCTCAACCGTACTCATTACCATAG 259

   281 TACGCTGCGCAACTGTAATCTACTTAATGAACCCTAA 317 (bp)
   260 TACGCTGCGCAACTGTAATCTACTTAATGAACCCTAA 296 (bp)
```

l

```
L1  1 CACACTCAATTCAAATCAACTTCAAATTTTTCATTCGTTTTATTCACCCATG 52 (bp)
L2  1 CACACTCAATTCAAATCAACTTCAAATTTTTCATCCGTTTTATTCACTCATG 52 (bp)
```

L1 vs. L2
96.15%
(50/52 bp)

m

```
M    1 TACACTATACTACCTTATTACCCTTAGTGAACCATAACACACTCACTTCAACCCAACTTCAAATTTTTCA  70
    71 TCCGTTTTATTCCACTCATGTACACTACACCACCTTATTCCTACACATTTTTATTCATTTTACTTTCTTA 140
   141 TTCTCCTACTCTTCTTATTCCATTATTTTCTTCCTCTTCTTTCTTCCATACTCTACTCTATCTGTTCTAC 210
   211 CTATTCTCCTCCTTCCTTCTCTCCTTTATTCTGCCCCTTCATTTTCTCCTTCATTTCCTTATACTTCTCT 280
   281 AACTACG 287 (bp)
```

n

```
N    1 TGATTACTTCTTCATTCTCCTACTTTATTTATTTTATTCATTTTATTTTATTTTATTTTATTTATTCGTT  70
    71 TTTAAATATTTAATTTACTCTCCTTACTTTATTTTTTTGATTTCTACTCTCTTCTATATATCCTACTCTT 140
   141 ATATTCTCTCCTTCATTTCCTTATTCCATATTGTTACTTCGTTATCCAGCTCACCATGACTGAGTCAACT 210
   211 TCAAATTTTTCATCCCCTTCAATTACCAAAGTCCACTACAATTCTCTATTTATATTCCTGCATCCCAACA 280
   281 CATTATTTCAACCACTTGTCTATCATAATTACTATAGTTCATTACTCATCCCTATTCTACTTAATGCACT 350
   351 CCAATATAGTCCCCTGCTTTTATCTTCTTTCCTCTTACTTTAATTGCTTCTTTATTCTCCTACCTTATTT 420
   421 ATTTTATTTATTTTATTTATTTTATTTTATTTTATTTATTTTATTTTACTCTCCTTACTCTATTTCTTCT 490
   491 ATATATTCTACTTATATATCCTGCTCCTTCATCCTACTCTCTTCTCTCTATATTTTACTCTTAAATTCTA 560
   561 TCTGTTACATTCTTGTCTTTCGTTAACAACA 591 (bp)
```

o

```
O1  1 TTTAACGATTACTCGCCTTACGGCTCGTTACTTGCTTCACACCTGACTAAGTGAACTTTGAATTTTTTTT 70
O2  1 TTTAACGATTACTCGCCTTACGTCTCGTTGCTCGCTTCACACCTGACTAAGTGAACTTTGAATTTTTTTT 70

   71 CCTTTATTAAAATGACCATCCTCAAAATTTCCCATCCTC 109 (bp)
   71 CCTTTATTAAAATGACCATCCTCAAAATTTCCCATCCTC 109 (bp)
```

O1 vs. O2
97.25%
(106/109 bp)

p

```
P1    1 CCCGACGACATATCACTCAACAAACGTTTTCAATAATTTTTTTTTCTTTTATCAATCATTTTATAATTTA  70
P2    1 CCCGACGACATATCACTCAACAAACTTTTTCAATAATTTTTTTTTCTTTTATTAATCATTTTATAATTTA  70
P3    1 CCCGACGACATATCACTCAACAAACTTTTTCAATAATTTTTTTTTCTTTTATTAATCATTTTATAATTTA  70
P4    1 CCCGACGACATATCACTCAACAAACGTTTTCAATAATTTTTTTTTCTTTTATCAATCATTTTATGATTTA  70

     71 TTTTCAATTTTCAACTGCCTGCTGTTTGTCGTTCATTGGCAAATAAATATTTTTCATTATTTTCTATCAT 140
     71 TTTTCAATTTTCAACTGCCTGCTGTTTGTCATTCATTGGCAAATAAATATTTTTCATTATTTTCTATCAT 140
     71 TTTTCAATTTTCAACTGCCTGCTGTTTGTCGTTCATTGGCAAATAAATATTTTTCATTATTTTCTATCAT 140
     71 TTTTCAATTTTCAACTGCCTGCTGTTTGTCGTTCATTGGCAAATAATTATTTTTCATTATTTTCTATCAT 140

    141 TATTTAATATTGTCTTTATTTGTGTTCCAATATTTGTATTTATATTTTTTATTTCATTTTATTTTG 206 (bp)
    141 TATTTAATATTGTCTTTATTTGTGTTCCAATATTTGTATTTATATTTTTTATTTCATTTTATTTTG 206 (bp)
    141 TATTTAATATTGTCTTTATTTGTGTTCCAATATTTGTATTTATATTTTTTATTTCATTTTATTTTG 206 (bp)
    141 TATTTAATATTGTCTTTATTTGTGTTCCAATATTTGTATTTATATTTTTTATTTCATTTTATTTTG 206 (bp)
```

	P2		P3		P4	
	Match /Total (bp)	Identity (%)	Match /Total (bp)	Identity (%)	Match /Total (bp)	Identity (%)
P1	203/206	98.54	204/206	99.03	204/206	99.03
P2			205/206	99.51	201/206	97.57
P3					202/206	98.06

q

```
Q1   1 TTAATTTTATTTATTTCTGTATATTTATTCGTTTTTAAATATTTTATTTTATTTTATTTCATTTCATTTC 70
Q2   1 TTAATTTTATTTATTTCTATATATTTATTCGTTTTTAAATATTTTATTTTATTTTATTTCATTTCATTTC 70
Q3   1 TTAATTTTATTTATTTCTATATATTTATTCGTTTTTAAATATTTTATTTTATTTT-----ATTTCATTTC 65
Q4   1 TTAATTTTATTTATTTCTGTATATTTATTCGTTTTTAAATATTTTATTTTATTTTATTTTATTTCATTTC 70
Q5   1 TTAATTTTATTTATTTCTATATATTTATTCGTTTTTAAATATTTTATTTTATTTTATTTCATTTCATTTC 70

    71 ATTTTATTTTTTTATTTATTTTACACAATCATATTATTTGTTTAACATGACTAACATAAGCTCAATTTTC 140
    71 ATTTTATTTTTTTATTTATTTTACACAATCATATTATTTGTTTAACATGACTAACATAAGCTCAATTTTC 140
    66 ATTTTATTTTTTTATTTATTTTACGCAATCATATTATTTATTTAACATATCTAACATAAGCTCAATTTTC 135
    71 ATTTTATTTTTTTATTTATTTTACACAATCATATTATTTGTTTAACATGACTAACATAAGCTCAATTTTC 140
    71 ATTTTATTTTTTTATTTATTTTACGCAATCATATTATTTATTTAACATATCTAACATAAGCTCAATTTTC 140

   141 AAACACTTCATTTATCAAAGTACACCGCATGTTCCTATTATACTTACTGCACTCCAACATA 201 (bp)
   141 AAACACTTCATTTATCAAAGTACACCGCATGTTCCTATTATACTTACTGCACTCCAACATA 201 (bp)
   136 AAACACTTCATTTATCAAAGTACACCGCATGTTCCTATTATACTTACTGCACTCCAACATA 196 (bp)
   141 AAACACTTCATTTATCAAAGTACACCGCATGTTCCTATTATACTTACTGCACTCCAACATA 201 (bp)
   141 AAACACTTCATTTATCAAAGTACACCGCATGTTCCTATTATACTTACTGCACTCCAACATA 201 (bp)
```

	Q2		Q3		Q4		Q5	
	Match /Total (bp)	Identity (%)	Match /Total (bp)	Identity (%)	Match /Total (bp)	Identity (%)	Match /Total (bp)	Identity (%)
Q1	200/201	99.50	191/201	95.02	200/201	99.50	196/201	97.51
Q2			192/201	95.52	199/201	99.00	197/201	98.01
Q3					191/201	95.02	196/201	97.51
Q4							195/201	97.01

r

```
R1   1 GTCCCCTTCATTGTATCTTCTTTTTTCTTACTTTACTTACTTCTTTGTTCTCCCATTTTTTATTATTATT 70
R2   1 GTCCCCTTCATTGTATCTTCTTTTTTCTTACTTTACTTACTTCTTTGTTCTCCCATTTTTTATTATTATT 70

    71 TCTTATTCCATTACTTTCTTCCTCTTCTTTCTTCCATATTCTAC 114 (bp)
    71 TCTTATTCCATTACTTTCTTCCTCTTCTTTCTTCCATACTCTAC 114 (bp)
```

R1 vs. R2
99.12%
(113/114 bp)

s

```
S    1 TCTACCTATTCTGCTTTACTTATTCTACCTATTCTACCTATTCTGCTTTACTTATTCTACCTATCCAATA 70
S'   1 TCTACCTATTCTGCTTTACTTATTCTACCTAT--------------------------------CCAATA 38

    71 TTTATACTTCCTATGTTTTACCTCTTTTATATTTCGTTAACATGTTTAACGATTACTCGCTTTACTGCTC 140
    39 TTTATACTTCCTATGTTTTACCTCTTTTATATTTCGTTAACATGTTTAACGATTACTCGCTTTACTGCTC 108

   141 GTTACCCACCTGAATCAAAACTGAGTCAACTTCATATTTTT 181 (bp)
   109 GTTACCCACCTGAATCAAAACTGAGTCAACTTCATATTTTT 149 (bp)
```

t

```
T    1 CATCCATCTTGATTATCATAATACACTGCGTATCTTTATTCAACTTACTGCATTACAACACAGCCTTATT 70
    71 CTATAAACTTCAAGTTTTCATTCATCTATCCTCATTCGCCATAT 114 (bp)
```

u

```
U1   1 TGCACTACATCCCCTTATTATACTTACTGCATCCCAACACACTCACTTCCACTCATTTCAGTGTTTTGTC 70
U2   1 CACACTACATCCCCTTATTATACTTACTGCATCCCAACACACTCACTTCCACTCATTTCAGTGTTTTGTC 70
U3   1 TGCACTACATCCCCTTATTATACTTACTGCATCCCAACACACTCACTTCCACTCATTTCAGTGTTTTATC 70

    71 CATCTTCACTACCACACTACACTACTCATTCTT 103 (bp)
    71 CATCTTCACTACCACACTACACTACTCATTCTT 103 (bp)
    71 CATCTTCACTACCACACTACACTACTCATTCTT 103 (bp)
```

U1 vs. U2 98.06% (101/103 bp)
U1 vs. U3 99.03% (102/103 bp)
U2 vs. U3 97.09% (100/103 bp)

v V 1 **TTAATTTTATTTCA** 14 (bp)

w W 1 **TCTACTTATTCTACCTATACCTATTCTAC** 29 (bp)

x X 1 **TAACCATCCTAATTATTATAGTACACTACTTGTCCCTATCACCTTTAACGTACTCCAG** 58 (bp)

Supplementary Fig. 3 Sequences of common segments in *SH-P* regions.

(a–x) Sequence alignments of variants and subtypes of segments A–X in Fig. 3. Nucleotide changes among variants are shown in red. Percentages of sequence identities among variants are indicated. Positions of common sequence motifs in segments C, E, K, and S are indicated by boxes according to Fig. 3b. Panel c* shows the sequence alignment of the common sequence motifs of segment C, c1–8. Nucleotide changes are indicated in red. The table below shows percentages of sequence identities between them.

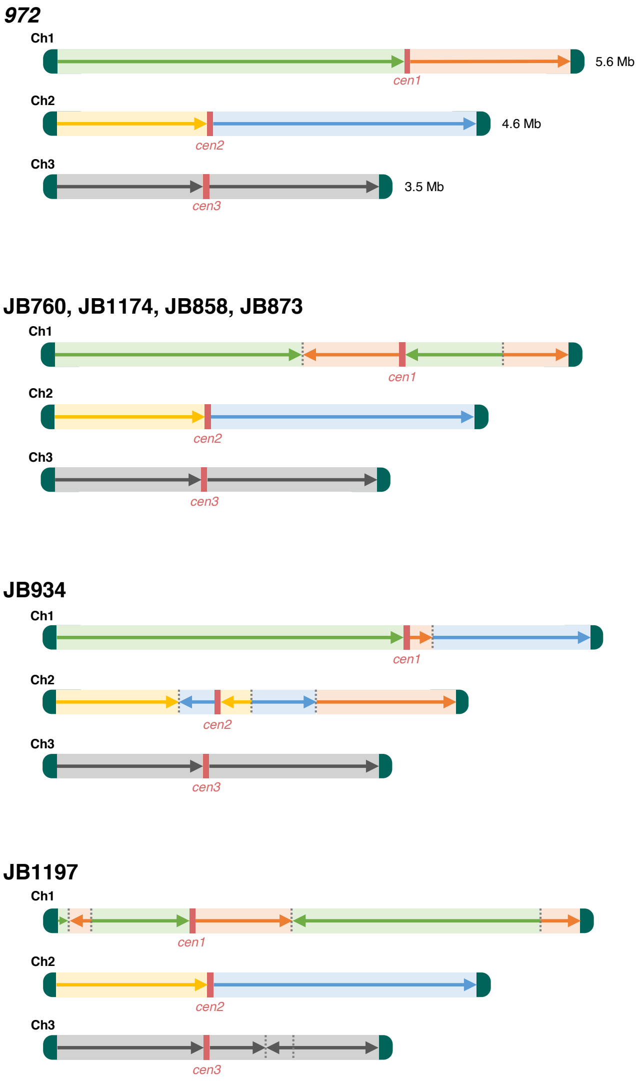

Supplementary Fig. 4 Chromosome configurations of *972* and JB strains.

Schematic illustrations of chromosome structures in comparison with *972*. Arrows indicate sequence directions, left to right arms, in *972*. Note that positions of *subtel1R* and *subtel2R* in JB934 are swapped compared with those in *972* (also see Fig. 4e).

α

JB873-*SH1L* (216 bp)

TATTTACTCTCTTTATTCTATTTACTCTCTTTACTCTCTTCTCTGTATATCCTACTCTT
ATATTCTATCTGTTGCCTTCTTGTCTTTCGTTAACTACGTTTAGCGAATACTCGCCTT
ACGTCTCGTTAACTGCCCAATTCACACATCGCCGACTCAACTTTGAATTTTTCGCCC
ACCTTGTTTACCAAATTAACTATCCTCTACATTTTTCATCCT

β

JB873-*SH1L* (574 bp)

TGTTAATTTTATCTTTTTTTTTCTATTTATCTTTCTATATTTATTCGTTTTTAATTCTTT
TTTTTTTTTTTTTTTTATTTTATTCAACACTACCACATTATTTGTCCAACATGACTAAC
TCAACTTCATTCAAATCTTTCATCCATTCCATTGATCATACACTATTTATCCTTATTA
TACTTACTGACTTCTAACATAATACAACTAATTCAGCTTCAATCTTCAAATAATGAA
TCTTATTGATCGCCTTATTGTTACTTTATGTATTCTAACACACTTTACCCTCTCAACT
AATTATCTTCATTCAATTCCTCCTCCTCTTATTCTCTTCTTCATCTTACCTTCTACTTT
TTCATCTTCATCTTATTTTCTTCTTTTTCTTTCTTTACTCTCTTCTCTCTTCTCTCTTCT
CTTCTCTTCTATACCTACTCTACCTATATATTCTATATATTCTATCTCTCCTACCTCTT
CTACTATCATATCTTCATTCCACTTTATTCCCTTATCCTTCCTTAACAGCACTTAACG
AATACTTGCCCTACAGCTCGTTACTCTCTTCACACACGTCTCAGTC

γ

JB873-*SH1L* (242 bp)

TATTCTATTTTATTCTATTTATTTTATTCTATTTATTTTATTCTATTTATTTTATTCTAT
TTATTTTATTTACTCTCCTTACTCTATTCCTCATCTTCTTTTCTCTTTTACTTCAATATT
CCTCTTCATCCTACTCTGTTACTTTCTTATTCTATTTATTTACTTTATTTTTTTTTATTT
TTTTTACTTTATTATTTATTTCCGTACTCTCTTATTTTATTTACTCTATTCTATATTCT
ATA

δ

JB873-*SH1L* (68 bp)

CATTTTTTCGTCCACTTTATTCCCTCGTGTACACTACATATCTTTATTCACATTACTG
CGCTCCAACA

θ

JB760-contig 1 (1169 bp)

GGCAGTAGAAATGTGAAAACAACAAAATGAAGGGATAAATAATGTATCAACAAAT
AAAAAAAAGTTTGCATTTATATAACGAATGTAAAACAGTAGCCAGCCCAGTCAGCC
ATCGCTTATCAAACCCTGTTTGAACCAGCCTAAAAAGTGAAATAGACCAGCCATAC
GAAATGACAGAAATGACAGCCATGTCAAAGAGCATCCAAGTATAAAAAGATTGAA
ACTTTTTTTCCAACTTGACAAGTTTCGACTTGAAACTTTTTTATTCTCCTCCCTCTA
CCCCTTTACATACTACCTTTGACCTAGTTTTCCTCGATTTTGTAGAAAGTGGTGCTAC
TTTGTGAATTTCATCCATATCGGTGCTTCTAGGGTAGGGTTTGACATTTGTCGAACG
CAATACGCAGTACTACAGAAAAATCCACTTCCCACCAAAGGGTTTTGCCTCACCCT
TCCTATTACTTTGAATACCAAAAGATAATCCATTTCTCTCTAAAATCTTTATGTATA
GCGAATTTGATAAAATTAAAATGATTTCTTTTCTTTTCTTTTCTTCTTTTTATTCAAC
AAATTTTTTTAAAGGTTCATGAAATAATAAGTTGTGTAACCAAACAATTCCATTTTA
TTGCAATGAAAAAAAAAAAGAAAAGTAATACAATTGTTTTTTTTCGAAATTTGTAA
TTTTATATTTATTTAAACAATTTCATTGAATAAACAATTTTTTATTCATTTTAATTTA
TTTTTATTTTATTTTTATTTTTTATTTTTTATTTTTTATTTTTTATTCATTTTAATTTATT
TATTTTTTTTTATTTTTATTTTTTCCTTTTTCCCTACTCCTCCTTATTCATAAGTGAGA
TACGACTCATTTCATAACTGAGATGTCCTTTTTTCCTTACTCTACGTTATTCGTAAGT
GAGATGGCATTGACATGGCATGGCATTGCATTGAAACGATGACTCTGGTCTGTTCT
CGTTCCATTTCATTCCCTCTCGTTACTCATCGTTGATTCTCATCGAAATACGTTCTCT
ATCCCTAACTCTCCCTATTCATAACTGAGATAGGACATGCCAAAACAACCGAGATA
GACTCGTTCTCTCTCGTTAAATTCCAATTCTTACGTCATTTCCCCATCTCACAAACCA
TCCAATCCTACCCATCTCCACCTCTA

ι

JB1174-*SH1L* (1840 bp)

AAAGTATTCCCATCCATTCCTCTCTATTTCCCTTTTATTTCATTCACTCACCGTATTT
CTCTCCAATCTCCCTTCACCCATCACTCACTCACCAACTTATCCAATCTCTCTTCACC
TAATCGCCTACTCTTCACCCACTCCTTTCCATTCACTTGATTCACTTGTTTTACCATT
CTTTTTTCACTTCCTCGTATTCAATTGATTTTCTTTAGCCATGTCGCTCCTTTTCATAA
ACTGTATGGTTTTGACTTTATAATTCAAACATTCTTGTAATTAGTATGGTGTTCATTT
GGTTAACGATTGAATGATAGTCATTTATACATTTCTTTCTTTTACAACAACAGCTAT

TATAATTTACTGTTTATTTCAACCAATTTATTTTTTTTTAAAAATCTAACATATATG
AAACAAATTCCTTATTGTATTCACAACAAGCTTGCATTTTATTGCAAAATGTAAAGA
AATATTTTTATTTATTATTTATTATTTATTATTTATTATTTATTATTTATTATTTATTA
TTTATTATTTATTATTTATCTTATTTTTATTTTATTTTTTTTTTTTTATCGATCTTAGT
GTACATGTCCCTTTTATAAAAAAAAAAAATAAAATAAAATAAAATTCTCTTCAAAA
AAAAAAAATATCATCAAACCACAACATATTGTCGAATACACTTTTTATAATATATTT
TCAATACTACGTGTAAAGTATTTTACCAACCATTTTTCGTTTTGTCTTAGTATTCAAT
TTAGTTCGTAATGTCGATTACCAAATTCCATCTTCTTTTGCCGTATTTTGTAACCACA
ATATATAACTCATCTTAGCATCGAACACTTCTTCAAGAACGAAATATGCAATGCTA
ATAAACGGTGATACTTTCTATCAATTCCTTAATATGGAATATCATGCAAACAACATT
TGAAAAATGTTGCCACAAATAAACATCCTTCCATTAATTTACATCGTTAATTAGCAA
TTGCTTGAAGGTATTTTTTTTTTTTTGAAACTTTTGACAGCATTTGGTCAGTTGGGAT
GGATGGTAATTTGACAAGGCACATGTCTTATACGCAACGGAAAAACATTGCAAATG
TTGCAATTGTCCCGTTTAATCATCCACTGAGTGGGAAACCATTTTTTTCTTGTTTCTG
CATGCATTCTTAGAAGATGCGCAGGAAACTTTTAATAATAATACGATGTCGGCTCA
ATTACAATTATTAAGCGAAACGAATGACAAATACTACATATCATACATCGTTTACC
AATTGTAATCGTACTGTTCGTGAATGCTGAGAAACTTTATGTCTAGTGTACTAGCAC
TTTTTTAACTTGCAACGCTAAGCATACCCAGACGCATAAATACATTAAGCCAATGG
CATGGCCATAATCACTTACTTTAAAGTCTTCATCCAAAGTTACGTACGATGAACATT
TGAACATCGTACAACAAGCTGGTGAACGTGGTGGTGCTGATTGTGATGGTGATGCT
GATACTGTTAGTCGCGCTGTGCTGGTGCTACTGGTACTGGTACTGATACTCATAGTA
CCATTCAACTTGGCAAGTATAAGACTTATGTGTGTAAGCGGTAAAATTCAATGAGT
AAACTCCACTAAAAAAAATTTTCACAACTCGATCGATTCTTTTGAGTTTACAAACAT
GAACTTTCAAGTTACACCTTTTCCATTAACATGTTCGTTGATTGTGTGGGTGTCGAT
CATAAGAATGTTGAACGAACTGTCTAATGGGGTAACAAAACAAATTAAACAGTCTT
TCTCATGAACTTGAAAAACTTTGAACTTTTTTAGTTGGTTCATAATTCTTGCACGCT
GTAGTACC

κ

JB1174-*SH1L* (1193 bp)

CTGAACATCTTGAGTTATGAGAGATTCGATTAGTATTTCAAACGGCCGAACCATTG
CATAATATAATATCTCAATGCCACAATAGAAAGAGAAACGGGTAAAATCGAGCAA
TGGGCTTTTCAGCAAAAAAACTTGTTGCGCGTTTTATCGTAGTGACTAAAAATCATC

AATCTTCCCAAGTAAACAAACAATTGCCTTACCGAGTCTTTAGAGTTTTGAAATGTC
CAGTACGATTGTTCTTGAGACCTAGCTGGCTGTCCTGCCGATATTGTGATTAAAAGG
TATAGTAACTCATTGAATTGGGACGCGAGTTGTCTGTATTGATTCAAAGCTGGCAC
ACACCAGGGAAGCTGCTTTGGGACGAATTGCAACCTGTTTTGATTTTCTTCCACTAC
TTGCTGAATATTGGCTTGAGTCACTGTGGGAAGGTACTTTGTCCGTAGTTCGCCATT
AATAAGAATAGTGCGAAAAATAGTCGAACCACACTTTTGAAGTTCAACACAGTGTT
TGAAGATTGAATAATTATTTTTGTATTGTTAATGTCATCCTTGATAGAAGTTAAGAT
AGATAAAGGGATTAGCTTTTCCACATCGATGGTAAATGTCAAGTCATGAAAGATGC
TTTGTAATTTGGAAAACATTGATTGATATAGCCTTTTCAAATCATCAAGACTAAACT
TGTACAAACCATTCACATGATCGCTAACATGTGGGTATGCAGAGTTCACTGGTTTG
AATAAAATTTCTGTTTTTGATATCGCTGCATAGTCTTGAGCTCTTCCTTCTGTTTTGT
TAATTCGATTGATGATTTTGTTGGTGTCACATAGTAAGTCGTAAACATTTCGATTCC
CACGACGAATATAGCAAAACAATTTGTGAAACAGTTCAGAACCCTCCGATTCCATA
TCGGTCATTGTCGATAAATTTACTTTTCCTTCTTTTATCAAATTTTGAAATTCGTAAA
TGCCACACAGTCTCAGTGAAAACAACAAACGAGAAATTAACGGTAATAATTCATAA
GCATATCGCGATCATCCATCTTCATAAATCGACGCACAAGCTAGAAATCTATTGCA
AGGCATATCCACAAGTTCTTCATTGTACTCATATCGTTTGAAAAAGGCCAGTTTAAG
AACATTGTGAACTTTTTCCAGTACCTTGTCATATTCCTTTAAAGTAAGTTGCGTACA
AACT

λ

JB1174-*SH1L* (1015 bp)

TCCTCTACATTCTCCCTTAGCACACTTCCTTCGTTTCCTTCCAAAGTGCGTAACAAA
AAAGAAAATGTACTGACTTGTTTCAGTGCATAGTTTTCCACTGTTGCTCCTTGTTCT
AAGGGTGGAAAGCCTCTTTCACGAATATTGTCACCTGCATCTTGGGTAAATAAACT
TTTGGTAAAAAATATCCATATTTCTTAGGTACTCCTGTAGGTTTTTTTTGTTGTATT
ATCCGTCCTGAATACTTTTTGCGACGACAAATGTGTGGGTAAAGTGTAGGGTAGGG
TTGATATTTCTCATTAACGCACTACGCAGTAGCACACGTTTTCCACTGTATTTTCGT
GTCTCTCCTTCCTGTTTGCTGTGATACTAAAGCGATGTACTAGTCCTTCAAATCGCTT
GATAAAGAGAAGAAAAAAAAGATGCAGCAGACTTAAAAAATGATAAATAGTATGT
TTAATGAATAAAATCGTTAGTATACTTGACTTTCATCAAGTTACTCACACTTGTGCT
TATTGTCATTTTATTGAACATGAAACATAATTTGAAAGCTACTCTAGTATTCATTTTT
TGCGAAATCATATTTTTTTGTGAATTTTTTGCATGTTATAATTTATTTACTTATCTAA

TTTAATTTGAATTAGTTTTGTTCTATTTATTTTATTTATTTCTACTCTAAAATCACATT
TTGTTTTAATTTATTTATTTATTTTATATTTTATTGATCCTTTTTTTTTTTTTTTTTTT
AATTTTATCTTTTTAATATAAATTCAATGCACAATGGTTTTCTTCGTACAACATTGTT
TCATCAACGACTTGTTAAAAATAAAACAAGTAATAGCTCCTTGTTTACTTATTATTA
TATGAAACCGAGATGGTAGATGGACTTCAAATTGAGTTCATTTGTGGTTCCAAAAA
AACTCCATTTCATCTACTTTACCCCCAGATTCCGCATCATATCTACAACATGATCGT
AAAGCTTGAAATACCATTTACCTGTACTTTGGCAGTTATG

μ

JB1174-*SH1R* (551 bp)

CGTACGCACTTGTAAATATGTAAAAAACGATCCTTTGCGTGCCATATCGAGTATGG
CATGGTTCTTTCACATCTTCTACTTTGTGTTTTACACGAAAATAGTCAACTTGATCA
AATCTCGCCTTTCATTACTTAGTTGCTTATACTAATGAACTCATGTTGATATTGTTTT
GCTATGTTAAAAAATATATATATATATATATATATATATATGTAAACCTAAATACAA
TGTAACACTTGTTCAAGAACGAAATATGCAACGCTGATAAATGGTGATGCTTGCGG
TTTGTTTCTAGTCATGAATTCATAAAAGTTTTATCTTTTACATCCGAATCTAAAAATT
TGTCGGCTATTGCTTGTTTGTAATCCGTTATATTCCATGCATGTAGTACGAATGGTA
TCAAAGTCCCGCGTGACCAAGTAGTACATAGACGAGGAAACGCTCCGTACGCTCCA
TTATGCTCCTCATGATTAAGACAACAAAACAAACAAATATCTTGGTATGTAATGTC
ATGCGGGCAACACTTGTAAGAGTGTTGTTGAAAACAATAA

ν

JB1174-*SH1R* (573 bp)

AAGTTTTTTGTTTTAACAAAGTCGACCGAGTTTTTAAATCATCTTTGAATTGCCTCTT
TGATGGGATAAATGTATGTGGGCTTTTAGTGTTATTAGTGCTATTAGTGACAGCAGT
ACTACTACTATCAGTAGTAGAAGTGGCAATACTAGTACAACTAGTTGTATTTGTAA
CAGACTTTGCCGCCATTTCGAACCACTGTTGGCGAAAAATCTACCAACGTACCACT
ACTGTTACCTCCATTTCCTTTGTCCGTATGAAAAGCTCCTTCCGCTGCCAGAACCTC
CTCTGCCTCACCATCCTCGTTGTTCTATATGATTGAGTGGCGAACACGGTAACAAAG
TCGATATTCAAAGTTGTAGAATTCAATTCGATTCCAAATTCACAAAGACTTGTGCAT
GGTAAACATCTTGAGCACTTGAGTGCATTTGGTAAAAGATCACACGATACAAACGT
ATTATTCATCCCTAATCCTATAAACGCTCGAGCACACTGTTCTTTGTCGTTTATAAA

TGATTGCATATCAGTATCAATATGCAGTTTTTGCAAGTTTGAAACTCCTTATACA
ACAAC

ξ

JB934-*SH2L* (1457 bp)

ATTCATAACAGACTTTGTCCCATTCGAACCACAATTGGCAAAGAATCTACCAACGT
ATTACTACTGCTTCCTCCATTGTTTATTTATATAACTGGCTTTGTCATTAATAAACG
ACTGCAAAGCACCCGTTGTTTACCTTTTCTTCCTACTAGACCTGTTTCCTGAGCGTA
ATCCATCACGTATTCCGGTGCTCCATAATGAACGCCCCGACTCGGCATCCAAAAAG
TTGTCCAAGATTCGCTTCACTTCCTTTTTCGTCCTACAACATATAATAGCTCGATTGT
TTTCCTCATCACAACGATAGCTTTTGTAAAGCATGACAAAGTCTCATGCCTACTCTA
CTACTCTATCTCCATTCATAAATGATTTACGATCCAATGGTTGTAATATATATTGAA
TGTTGTTTTTGTTTGTCAAAGATCGAACTGTTTATGAAATGGGTACAAAACATTTCT
GTTAGTGTCTTTGCATGTGTGATGAAAACGTTGTTAAGATTGACTATGAACAACAC
ACTTCAAGTATGATCGGTAAGCACAGTGTGAGCTTTAACAATCAATACTCTCGTCA
ATCACTACTCTTGCCAAACGATTGTTCCTAAAAAAGCAGACAAGTTTCAGAAAAAG
CAGAATCAAAAGTCAAAATCACGGTATTGGGAAACGAGACATAATTCTGGGTGTG
AATTTTTAAAACATTTCCAATCATTACTGCAATTCAAACCAGCAGCATGATTTTGTT
GCATCATGCCCTATTTCAATGAAATGAGCAGAGACCACTTTTCTTTTCCATATAAGC
TGGCAAAAGATATGTTAACAACTTTCCCGCACCCGTCGGTAACACTACCAGCATGT
TTAGTCGACGCAACAAAGAATATACCACGGCCCTTCCTTGTTCCACCGACTTGAACT
GTGCATTACTGTTTCGTAAAACAATCTCAGACTTGCGATACAACTTTTATAAATCC
TTTTCGGTGGGCATATCACTGGTTGTGCTTTTGGCGTCCATGAATATTCACATGATA
CATCTCGGGTGTTGTGGTTAGATGAAACGGTAGTAGACGAAACGAATTCTGAAAAT
GGTTTTGACGACAATTTGGTTGAAAGATTCGTATTCAGACTAGTTGTTGTTTGCATG
AGTGGGACAGACCGTTCGATATCCAATAACTCCTGCCATAAAGAACTCGCTTTATA
AAAATTAGTAAAAAAGTTTGTGGGTAAATAATTAATTCCTTCATCCAATCGCGCAT
AAATATTCAAAGCTGTTGACCTCGTATGACCAGCCTGTAAATCAAATGTAGAGTTC
CCTTCCTCCTGTTTTTCCAAATTGTATTCTTTAAAAAAATGAGCAATGTGTCTATAGT
TACGAAAACCTAAAGATTTCCTATAACAAACAATTGCCTTATCGAGTTTTTAGAGTT
TGAAATGTTCAGTATGATTGTTCTTGCGATGTTGCTGGCTG

O

JB1174-*SH2L* (4838 bp)

TCAATTTTCATTATCACATTTATTGAAATCAACTTATGAACAACTTGCTTTATGACA
CACAGATTCTTAGGTGTTGGTGTTTAGCTAGTTAATATTGACGAGAAGATTTCTTCA
ATAAAAGTTTCACAAGATGAGTGTGCTTAAAATTAATGTGTACATGGTCAAATACG
GAACACGTTAATTTTGCAATGCGTTGTGTTTTTTTATGAAATGCAATCAGGGCGGAA
AGGATCAAATTTATTTAATTATAAATAGGTCATAAACTGAACCTCCTCTAAATATAA
ATGGTTAATATCTCATGAGCTAGGATTCAAATCCCAGGTTATGTTTTATTTACGAAC
ATTACTCGATTTGTTGGTTTTCCATATTTTCATAGAAGGAGTTACAAGTTTTATACA
AAATATTAACGGACATTCATATCGTCCTTTCTCACGACGACGACGATTGTTCAGACG
AATAATAGTGGTTCTCAGATAAATGGTACCTCAGACTCTAAAGCTGTAAAATGTTG
TTGATTTACAAGCGCTTGATGTCAGAAATGTTGCGTTTATTCAGCTTATGCTTTAGG
AGATGCCGCGATTGGTTTCTTTGTAAGCATCGATGATTCCCAATATGCTACTACCCA
CGCAAACGAGGGCACTCACCAATGTAACTATGATACACACAGTATCTACAGCTTAA
ATGATAAACAAAAAAAAAATATATATATACATATATATATATATATATATCAAATA
ATGTTACACTTCAGAAGAAAAAAAATATGATATCCAATACACGGATAGCGTTTTAC
TAGTTTTTCATTGAAACATGAGTCCGACCTTTACTACCGATACAAATGATCTCCTTC
ACTGCTGAACCATAAGTTTGAGAAGTTTTTAGGAGAAATCTTATCAGTCAGCTTACC
TGATCGAATATTTAATTCGCTTAGAATTTGGTTTAGACTACTATGCTTTATAAGCAA
ATAAACATGTGTATATTCGCTCAATGAATATGTATATTAAATTCAATTATTTTCGTA
AACAAAAAAAAGGATGACAGATTGAGGAAAATTAGTGAGTGACTCCTCAACAATA
GGCAAGTCCATGATGAAATAAAAGCGCACATTGAATGTATACGAATGCAGCCCTCT
GAAAGTATTGTAACACGGAAGAATGAAGCGTAATGCTTTGACTTAGAAACTTAATC
CTTTTATCAAGATGTCGTGCTACTTTAATAAAATTGCTATTACCTTGCGCTGAAAAT
ATCTCATATTTTGTTTACAATTAAATAGCTTTGAAAGCTCATGTTTTTGTACCTCCTT
CTCCCTATTAGATAATTTGATGTACAAGTCCTTCAAATTGCTTCGTCAAGCAAATAC
GAAAGATAAAGCAGATTTAAAAAAAAAAAAAATAACATATTTAATGAAAAAAGTAG
AGGACTTGATATATTTGAATTCCACAATTATATACATTTTACTTTATAAACTCCTTA
AATGATTAAAAACTACATATGCAAGTTATTCCAATATTTTATTTTGTGACAAAAATA
CTTTCTGTTATTTTCAACCTCAATCTTGTTATAGAAGCTGGTTTTGAGCGACTTTTTT
TGATTATTGTTTCAATAACATCGGCTACTATTTTGGTATACAGCTCTATATTAATGG
GGAAATTGATACGCTACCTACCCCAAGCAGGTCTTTGGCGACATGTGTAGAGGATG
ATGTTCTATTTAAAGATCATGATGTTAGTAATATTTCACCATACAGAGCAGGGGAT

GACTTTCAGTACGTGATTATAAAAATGGGGTTAAGAACTGTTCAGGTAATCCTGTG
AATACGCGCAGTTTGGCATTGGAGCTTACGATACAATCACAGAAATACTATACCTC
CATGTTTTTTTAAAAGTATTCAGCATGTAATTACTTATTATGGCTTGTAGTCACGTTA
CAACTGTCAGAAAGGCGGGAAGAGACAACTAGTCTTACAACCTCACGCGTCAAAG
TGTGTTGGAGGGGTGGATTCAACATCTATTCTGTAGTTGGGCCATACTTGATTAGTT
GAAACCTATTAATGATTGAACGTATTGCAAAATATTCTGAAACAATATATGAATTA
CGAAAACAAAAGAAAAAATAATGACATGGATGATCCAACCGTATTTGTTTAAAAA
AAGAGAAGAAAGTGGTAGGATAAGGATATAAACAAAGTTTTACCAATTTGACCCT
GAAAACAATGCTAAGAGGATATTCTGTATGTCAAGTTGATGAAGAGGATTAAATCA
AAGAATGGAAATAATTGGAAAGTAGTATTATATCGAAGAAAAATGGGAAAGCAAA
GAAAAGAAAAGAAACGTCTTTAAGGTTGAAAAAAGCATTCGATAATTTATACATTT
TAGTAAAAGACGTTATGAAATGGTATCACAGGCTGAACTCTCAGAATTCAAGTGCT
TAATACTCTGTCTTTTTTTGACACTAGTGCTCAAGTAAATGCTATAACAAAAGAAAA
TTTTTAAAGCCTGTAAATTATATACCAAACCCTAATCACAAAATGTAACGTATGGTG
CTGAATTACCTCATTCATTCAATAAACAACAGCCTTGATTTATAATATCGTGTCCAA
AGTAGTGTTAAGTAGATACTTTGGCGAAGATCCAACTAGTTTATGTGACGACTTTAT
TGGTTATTTCACAATTTCCAAACTAATTGAAAATATATATAATTCACCAATAAAATA
ATACAAATGATCCCAATGATAAAAAATGAGCGTAAACTTAATCTACAAGAACAGC
AGTGGTCACCAAATAGACCCATAACAAAGGAAAGTAAAACAAAGCGGTCAAGAAT
GGAGCTACAAGTTTACGATGTTAGTAATACAAATTAACAATATCATAGAATAATAC
TAAGATTAAAAGAATCTTTCGACTTAACGCGAAAAAAAATTTTTTTTTTTTTACCT
TTTCTCTTCAGAATTTTCATTATCCAAAATAATTCCAGAATTCACTTTTGTTCGACCC
GTTACATCCTCAAACAATGCAATCTTTATGTTAGTAACAATTAAATTTATGGTAATT
TTCATTCTGGTAATAACATAGTCAGTCGATTACCAAAGAAACTAGGACCAAAGGTC
CGTATATATATATATATATATATATATATAGTAAAACAGACTAACCGGGGATTGTG
GTAGATAAGAAGGTTGGCTGCTTGAGTTTACGTCATAAAAAAAAAGTGTAATCAAC
CAAAAGGTTTTTTTGTAGCTTGAGTTAGTCTTCGCCCGCTTAATAAAACGTGTCTGA
TTATATTATTTTAACAAAAGATAAGTGGTAAGAAATATTCATTCCTCGGATAAAAA
AGTTATTTATATTAGCACAAGTCAACTCACATTAAAATGAATACTGCACTTTTGATT
ATCTAATTCTTTTGAAACGCCCTTGAACGTTTTAAAAATCTTATGATAACTACCCGC
TCAAGTGCCATAGTCAATCAGGATGAAAATATCTAGCTACTATATGATATATAATTT
TCTTCGAAATTTTTAACATATTGAAGTATTCGTAAACAATGTTTAATTCAAAGCGGA
TCACAATATTCTGATTTTTTTACTGACCATAAGTAGCGTTGAAGCAATCAACCGTTC

CTTCACTGAGATGAATCTAAAAAAGAGTAGAAAATTTACGTGTAACTAAGAAAATA
ATTTTATAATGTTAGACTAATAAAATTGCTCGATAAAAGTTGTTTTAAAAAAGTTA
TACAATGTAACGTTTCTAAACACTTTAATCAATATAAAACAACAAATGCAAACTAT
TCTAGTATTCATTTTGTGAAATCATATTTTTTTGTGAATTTTTTTGCATGTTAATTCA
TTACTTATCTAATTTAATCCTGTTTGGTTTTATTTTATTTATTTCTATTTTAAAATTAC
ATTTTTTTTTTGTTTTACTTATTTACTTTATATTTTATATTTTATTGATCCTTTTTTTTT
TTTTATCTTTTTTAATATGGGTTCAATGCACAATTGTTTTCTTCATACAACATTGTTT
CATCAACGACTTGTTAAAAACAAAACAAATAATAGCTCCTTGTTACTTATTATATGA
AACGGAAATGCCAATTGAGATGGTAGATGGACCTAAATGAAGAGTGAATGTCCAA
CCCCACTTACGAACAGCATTTCGACTCCATTTGGGGTCAACAAGTACTAGGTACTTG
ACAGCCCACTTGGTAAAGTCCTAGCGTGAATTCAGCTACCCCTCTTTCCAACAAAG
AAGCAAACATATATTATACTAACTTTTGGTTAATTTTTTCAACACATGTTTGTAATA
TAACTATGAGCTGCACAATTATCAGTCCTTAAAAGGATTCATAATCTATGTCATTAG
TCCCATGCTTGCTCAGATCACCAAATCGTGTAAATTTCTGCTTGACAATGTAATTGT
AGGGTATTTCCATTAAGTAAATTGCTAGCAGGTAAAAATGTCACGGTTTATAAGTT
AGGTCGAGATAAAGATCGAAAACTAATATGGCCAGTATTATTTTTGTGTCGTTCAC
AATCAACAAGTGCTAATTTAAAATATAAATATAAATATAAATATAAATATAAATAT
AAATATACTAGCTACTAATTAAAACCGACAAAATATATTGTATCAAAAAAGCAATA
AGTGTAACTATTACATTACTATAATGTTGTAATAACGTAGCAAAAATGTTAAAATTT
AGAATTCGAAATATTTGAGCAAAATTATTTTGAACAAGACAAAAAAAAAATCATAC
TAAACATAAGGCTTTTTTTACTGCCTATCTCTCTTATACGGTTTCCACACATAATCG
AATATTAATTTTAGGAATAAATTAAATG

π

JB1174-*SH2L* (570 bp)

CGGTAGTTTTAACGTAGTGCAAGGTAAATGTGCATGTTGGACTTGATGTATTGGGG
TAATCTCAATATATGTATGTACCAATTGTTTTTAAATCGTATGAACGTTTTACAAC
CGACTTTGGTTTGTTGAAGCATATAGAAAATAATTACTTTTTGATTCGTTAATCAT
AATCGAAGCAAAGAAACTTTGTGCTTTCCAAATATGGATTGACTTCTAGGAATGAT
ATCAACTTTGAAGGATAACGTAGTTTCCACCCCTAAGGTTGAGTAACGATAACATT
ACCACATTGGAGGGATACTTTTATCTCTTCTTTTCATTTTCTTTTCTTTTCTTTCCTGT
TATGTTAAGCTGACTCTACTTTGTGTATCAATGAAATGTCACAGGTTAGAACATGAG
TTAAAACCCTTATATTTCGGCTAAATTCGAATAAACAGGAATTTTTGGATTATTGTG

AAAGAAGTAAAAACTTAGATTTAAGACTGAGAAGATTAGCGAAGGTTTAAACTATC
ACTAATGAATCCACATGATAAAATAACTCTTCTTGAACTATAATAAAAGCAAATGC
CGA

ρ

JB934-*SH1R′* (6406 bp)

TTCCGCAAATACGGTAGGCAGGAGTCCTAATCCAAGAGATTGGATACCTTGATCAT
GAATGCTATTGACGAAAACGGGAGAAGAACCTAGAATAGAAAGGAGTTGTCCGGA
ATGAGCAGTAACAGTATGCGCCAAATCATCTCTTTCTAAGTTTGGTCGATGAATGTC
TAAATACCCAGCATTGGATACTTTTTGACACAAAGATCCTCCAAAATAAACATTTAT
TAGTTGGAATCCTCGGCATATGCCCAATATTGGGAGTTTACGTCTCATTGCTAAATC
GACAATTGCCCACTCAAATTCATCTCGTTCAATATCCATTGAAATAGAAACATTTTG
GTGAAATTTTTCTCCATATCTCCTTGGATGAACGGATTCACCTCCAGTTAATACAAC
TGCATTGATTTGTGCTGGTATCTTGCTTATCGGAAAATTTGGATACACGACGATTGG
TATTCCACCAGCCTTATTTATGGCCTTGATATATAAGGGTACTATATTGGAATAACC
CACACTTAGAAGAACAACAGGTTTTGACATGAATCACTTGCCAATTTTTATGCAAA
GAAATATGAGAAATGAGTAAATGAATAAATGAAGAAGCTGTTATGAAATAAAATT
AAAAAGAAAGTTCTTCTATTTCCAGTTTTATAAAAGAAAAAACGCTAATTGTATAC
TCAGTATACGTTCACCCTACCAAAGAAAAGATAAATTTTTTTATTCACGTTACGCTG
CTCATGAATATATAGAAAATCGGCTACCTTATTTGCAAGGTTTTTGCTAATTTACGA
ATCCCAGCTCCGAAAAGATTTTGTCCATTTTTTCTACATAATTGTTCATACTTAGCA
ATACCCGCGAGAGTTGTCGTGATTTGTATAAAATATGCTGAGCTATATTACGAAAA
TTACTGAAAAAAAAAGATTAAAAATTATTAACGGTTAACTATTGATTAAAAGTGAA
AATATTTAATAACCGAAGAAAAAGAAACTTAACAAACGAATGAATTTAATCGTTGT
GCTAACGTTTACAAATGTTCACCAAGTAGAATTGTAGGGATTTAATTTTACTACAAC
TACGCAGATCGGTATTTGTTTTACGACACGTAAAACTGCTGTGAGTTTATTTGTGAT
CTAGTAAGTAGCGGTAACCAGGATGTAGGAGTTACGAAAGTTATAATTATTGTTAA
AAAATGAAAAAGTCTTTAGTTATAACTCATGTTTAGCTCACAGCTTAATTAAGGAA
CAAGATTTAGTGGGAGCATTATTGGCAGCAGGTCTGTCTCAATATCAAATTGACTA
TGAGGCGATGTATCAATCGAGCATGCGATACATGGTGATAGGCAATGCAGTTTAGT
ATTGCTAACATTTACGACTTTATTACATAGAGAAACTACTAGTTAATTATTTAATAA
AAAAAAAATATGTTATACCTTATCTACGCTATTGTTTTTTATCTCAAACAAGGAAA
ATTATACTTTTGAAACCCACAATAATTTGATAAAAGAATAATTCATGAATTTGCACT

GTGAAGCAAAGGTAAAACGTTTTGCTCATCTCAAAGGAATATAAGATTGGATTGCG
TTGAAAAAGGTGGTATCAATAAAGCTATAATCTATTTTATTTGTTTTACTAGTTATT
TTCATTTTCAATACTTTAAACATTTAGCAAAATATTTTATAATGGTTAAAGGAAACG
TTTTCGCTAACTAGCAAAAAAAGCTGACGTATATTTTGAAAGAATTGATATACTAA
TAAGAGCATTAAAAGTTGTTCACTTTTTGATTCCGTTTCGAATCAACTTAATTAAAG
GAATCCGGTTATACATACGAGATATAATCCTGATAATTGCTAGTTGAATAAATAGT
TTGCTAGTAAATTTAAAACAATTTGGTCAATGGCTATACAGAATAAGCTACTGTTCT
TTCAAATGGTATTTATAGAAATATCGGAAATAAAACAAATAGGAGCAAATTGTACT
TAAATATTCTTTATTTGAACTGACTCATTTGCATTTATATTTGTAACGTTTAAATTTT
TATTTATATTACATTTCGAGCGTAAAGTCTTATTTTGAATTTCAAAAACCTTGGAGC
AGAGAGTAATTTATCTTTAATTTTTGGAGATATAAGTGTATGATGATATTTCTTATT
TTTGTAGATTCTATTTGTTTGATTATTCCTTTTTAACGCTTATTATACTCGCATCATTT
TAGTCATCTTTACTATACCGATTTAATCTTTTCCTATACTAAACTATTGTTAAGTAAT
TAATACAACAGAGACATTAGACATTTGTCGAAATTCATTTAGGCTTTAATATTCTAA
AAGTTCATTACCTTCCGATTTATTAACTATGAATGCGGTCACTTTGTATGTATCCGA
ATACTTCATATTTCTATAAAAGAAAATTGACGATTCATGTTTGAAATTTAGAACAAG
AGAACTAAATGATTAAAAATATTACAAAAAATTTCTAATACACAAAATCAGATTAC
TATTTGATGGCCATTTACGCTTTGCTTAGTATGTTGAACACATTATTCTTAATGTGAC
GATATAAAGAGTATGGTTTCTTAAAATTTTTAAAAAAAAAGGAAAGAATAAGCGA
GTTGACATTTACTTTGTTACTCAAGACTCACGATGAAGTTTTTTAACGGCCTTCACT
ATAATGCAATTTAAGTTGCGAATGATACATGTATTGGGAATGCACTTAATAAAAAG
ATCGCTTCTACATTTCACATTTTTTATGCTTTAAGTGTTCATTTTCGACCAATGGTGG
AAAGGGTTAAAATTGCCAATTCGAAAATTATAATTATCCTAGAATTTTCTGAACCC
AGATAACTTTACACTCAGTTTAAGATTGTAAAATTAATTGTCATGACATAAGTATAC
GCTACGCGAATTTTTTTAATAAGTACTTGATAATCAAAGCGAGCAAATGTTTGGCA
AAAATAAATTAGATACTATGCTTACGAATAGTATCGTATTTGACAGTGTTTTCGCCA
TCCCCTTTTGACATACTAAGTTGACTAATTTTTTCATTGAAAGATATTAAATAGTGA
AGCAAGTTTAAGCTACTACTTCCAGCATCGAACGTTACGATAATGATTTTGGGAAA
TATTAAATCAAATAATATAGGTCCCCCCCGCTCAATGCTCCTTATTTGTTATACTAA
TTTCGTGTTACTAGTGTATGTAAGTATGCAATTCCCGCTCTAATACTTTTTGGACTA
GATTTTTTTTTCATTCTTAACGCAGAATTTTCCACTTTACTATTGCTCTCAGAGCTAC
ACAAATCTAAAGCTAAAATGAGTACACATAAAACCCATAGTCCATTAAGTAAAATT
TTGGACAAGACTTGTGAATTGTTTTGTATATCAGAATGCTATGAAATTTTATAGCAA

TCAACTTTGCTTATTATATACGTTTTATCAAATTAGCTTTCTATCATCAATACGTTTA
ATATTTTGAAGGATCGGATTTTTGGAAATTTCGAATTTGCTTTACGTATTTGAAAAA
AAGAAAAGAAATCAGCAGCCATATGCAACTAAAATGAAAGCAAATAAGTAAAACC
TCACCAATAACTAAAATTGGCCAAATAAACTTTCAGCAATTAGTGGAAAGAAGGCT
CCAAATAATAGTTAAAAATTTTTGACAGTTGCCAGTGCACTGTTGAATAAATTTCAT
ACGTATCAACTACACAAATGTTAAAGGGATTAGAATCATAATGCATCCTAACCTAA
AATGGTTGGCCCAATCACAGGTACAAACCAAACAATTTTGTATTGGACAGTCCAGC
CAAAAAGAAGAATACCAATTTCAGAAAGTGTGATCCCAATGAAACCCATGGGAAG
CTTAAATTTGGGTTTGCTTTCACCTTTCTTTCGCTTTATAAGGTATAAGTAAAGTTTT
TTGATTAAAAGGTAAAGCGGTGAAGACCGCTGCAAAAGGTTCGAGGATAGTGGAT
ACATACCTTAAACCACTAAGTCCAAAATTAAAATTGCAAAACAATTTCCATACTTC
GGAAAAAAGCTGTAAGTACCAAATTCACCATCACATATATAGAGCCAATCGTCAAG
GCTATACAATTGATATAGGTTGCGATATTAGCATGTGAATTAGATTTTTCATACTTA
ATAAAGCTAAATACTTCTTTAGAAATTGCTCGCCAGCAAGACGAAATGCCACTGGT
TCATTTTCTCCTATATGACCATACTTTTTCATTTTTTTTAAAATTATGAACAGATAAG
TGAATTTTTTTGTAGTAAAACTAAAACCCAACAAGCTTAAATTCTGATAAAATTTAA
CGAAATTAAAGCTCTCATTGCCAGCCAATTGAGCTTTGTCTGATGAATCATGAGATT
ATTGTAGCAATTCCTGGGCCGCCTGAGAGAAGCGCAAAAAAAAAATTACCATGCCT
TTCCCTTTATCCTTTTTGCCCGAACAGGTTAGCTAGTGAACCAGATTCATTTGGTAG
GCCAACAGATCCTAAAAGTCCGGAAAAAATCCGGGATACCAAAAAACATTGGCAA
ATTTACGGACAAGGCAACATGAATTAGTAAAAGCGCGACATACTGTACCCCACTAA
ATATATAGACTGTCTACAAAATTTTTCACTAAGGGGAGCAAAAACTAAGTTTCCAG
TTAACGTCCCTAAGGTATAAAGAGTAGTTTCTAAAGTGACTAATATACAAGAAGCA
TGGAAGTGCTTTGCAATATTAGGAACACTAGGTGCAAAATGCTATTACAACACTGG
TTTGAAAATATATAGATCCGATGACAACCACATTCTTCACTTTTATATTAATTGGCC
ATAATTCGGGATTGCATTTGTACTGTAATTCCACCTGTAAAAGATATGGATGGTGA
GTCAATTATACAACGTAGTTCAATTTCTTTTTTGAGGCTTAACTTTTGTTCATAGAA
AATAAATGATATACAATTTTGAAATATTTCAACAGTCATTTCTAGAAATTTTTATTG
TTTGATTAATAAAACAAATGATTTGATTAATTACAAGGTGCATCCGGCATTACCCTG
AGTTGTCATTTATTCAAAAAAAAAATAAGTTCAAGATTAAAAAGAATTTTTTCAGTT
TATCCGGCACTCCACTGGATACATTTGGAAACAAAACTTATGTATTAATTACTAGCA
TAAATAATCCTTATTCACGGAAAACTCGAACTACGGAAAGGGTTCCGCGAAGCTCC
GCGGTAATAAAACGGAGAATGTTGAGAAGTTAGGAATATTTGATGACATGAATGCT

CTTAGTTAAAAAAAAAAAAATGATTTTTTTTCACATTTGTTAAAGTTTTACCTGTCAA
AAATCTTATATGGTTCGGAATATTTGCAACGAGTTGAAGTGCGAGAAAAACATCTA
TTCTAAAATGAATATATTTAAAAAATGCCAATGTTAACGTAGTGAAATTGAAAATG
ATAAAAGTTATTTTTGAATCCGAGGTTGGAATTTTTTTAAGAAAGTAGTAAAGCCAT
GTGATAGGCTTGTTATCGAATGAAGGTTTGATGAGCTAAATTTCCGTTTTAAAAATT
TCCTATTGTACTAATTATTATTCTCATATGATTATGCACTATAATAATAAGAATACT
ATGAATTCTTTTGATTTTTCATTCATTTTAATCTTTGTGCGTTCAGTGGTTTACTGCT
AACAGTATTTTTTTTGTAAGTGTTTTCATCTTTGCTATAGCTCATAAGTAAATAAAG
TATTTTTTTATATATGAGATAATTATAAGAACTTTTCAAAAAAATTTAAAAACCATC
ACGAATGTCGGACTGTGCAGTTGGAAATTCATTTTCAGAAGACTATCCATATGCTTT
AATAGTTGGAGCAGGGCAAAGAGTTGCCTCATTTCAAAAAATTCAAATAAAGTTTG
ACTACGTGTCTAACTATAGTACAATAATAAGATTATTTGAATTTAATAAGAAATCCC
AAAATTTCCTGAATTTAATCGAGAACGGTAGTTTTCATTTTCTATTCGTTATTTTTCA
TAATCAGCTTAGCGTTCCATATTTGAATGCAAACTGTGAGGGTACACAGAATTAGT
TTTTTAATCTATAAATGTATAGAATCATGTCAGCAAAAAAATGTTCCAAATAACCGT
ATCACCAACAAGTTATTGTAACTATGATATATTAGGACGTTGTATTCTACGGCTTAT
GCAAAAAAGCAAATATTAAAGCGCAAATATTTAAAATTGCAATATTAAATTGGAAA
ATTACCCGGACATATTAAAGATAGTTCGTAGATCAAAAAAGGTTGTCAGTTATTGT
TAACACCAAAAATATAGACACATTTTAAGTCGGTAAATACACGTTGCCAAATGATT
TTGTCACAAGTTCTTTTAAAATTCAAATACAACTTTACCTAACTTAGACCCAAGTGA
CTTTTTAG

σ

JB858-*SH2L* (450 bp)

TGGGGGGAGCAGGTAGTTGTCGCTCATTAGAAGTATCGCAAGCCTCACTGTTTGCC
TCAACAATGTTTGCTTTATCCTTTATCTCATCCTGAATGAACTTGTCTGTTTCATAGA
CGACAAGATCATCGTCTTCATATCATCTGCTTAATTATTTTTCTTTATTATATAATTG
ATGGTTGGAAGTGAGTATCGACATTCCTTGAAATGTTCAACGATTCTTTGTACGTTA
ACAAAATGCTTACATACAAACAAGCAACTGACAGACACCCACTTTGTAAAAAATAT
CCGTATTTCTTATGTACTCTAATCCCTTCCAATAAAATCGGGTGAAAGCAGCAACAA
CGAGTGTAGAGTCGGCGGCTTGATGAATTTGAGCTCATACTGCACCGCTAGTTATT
GCCATCGAAAGTATTCGTTCACCAGACTTCGCCCATGCATACTATTCTTACG

τ

JB858-*SH2R* (1280 bp)

GAATACATAAGCTTGAATGAAAGCAATACCAAAAAATACTAATAAAGAAATCGCT
CGATTATCTTTACGCTTAAGTATTTCTATTTGAATTGGTGCTACTATTTTAGGATTAC
AGCAACATAAAGCTAGGCGAATTGAGTTTCTACGTAATAATCTACTTGGATGTTGC
TCGCACTCGTAAAAGCAAACTATGAATTAAAAAAAAAAAAAAAATATATATATATAT
ATATATAAAAGAAAAAATAAAATAAGATTGGTTTTGTACATGCTAAATTTCAGTCT
TCAAATAAAATATGATGCGTGAACAACCATTTATTTGTGTTTTCTTTTTTCCTATAAT
CAATCTTCCCACAATATTTCAATGTTAGCGGTATGCAACGTGGAGCACGGAAACTC
CAAAACGAAAGGTATAAAAAGCTAATCATTCCTAATCTGCGTTTCCCTTGAGCAAG
CATCATTTATCAACTTTTTAAAGATGCGTCTTCATGGAGCACTACTATTAATTTTAG
GCAATCTTGCTTTCCACACTTTAGTTAGAGGTGAAGTAGACGTCAATTCAAGTTCTC
TTACTACTGAAAATGAAAATCCTACTTATGAGGAACTAGGTTGGTCGCCTGAAGGC
ACCAGGAAGCTTACGCTTAAGTGCAGTTGGTTTACTGCGAAAAAATCGAAATTCAA
GAGTTATGCTGAATTTTTCGTGCATACCTTCGATGATGGAGAAGTTGCTTTATCTTT
TTTCAAGGCAAATGAATGGCAGGATGTAATTGGTTGGAATGAACAAGTTGAGGAG
ATGATCAATTTTGCAAAAGCAAGAGATACGGTGGTTGAAAAGTCTTTTTCTATGGA
ATCTTTTTCACAGGATCAAGCGGTGGGTCCGTTTGTATATCTTGGTGACTCGAAATC
TGTATGGACGGGTGATAATAGCATTACAAAACTCGTTTGGTAAATGAAACCGGTTC
TTACTGTATAGCTGCTAGACCAATGTCAACAGATGGACCTGAATTTGCTTTGGGCG
AAATGAGATATTTGGATTGGTACAATGACAGTGAAGTTTATCATCACCAAGTTAGT
ATTTTCGATTATATTTCCTTATTACAGATGGGAGGGGTATTCGCATTTTTTATATATT
GGATATGGGCTTGCTTGCATTATGGCGGGATAATTCTTCCCTCCCAAAAGATAATAT
GCCTTTATATTTTTCTTTTTTGCATTAAACGAAAATTTATGGCATATGTTTTCGCTAC
CATTAACATTTTCTCTCTATGAAATTAAAAATAATGC

Supplementary Fig. 5 Homologous box sequences shared by the subtelomeres in JB strains (except for JB22) but not in *972*.

Representative sequences of black boxes (α–δ and θ–τ) are shown.

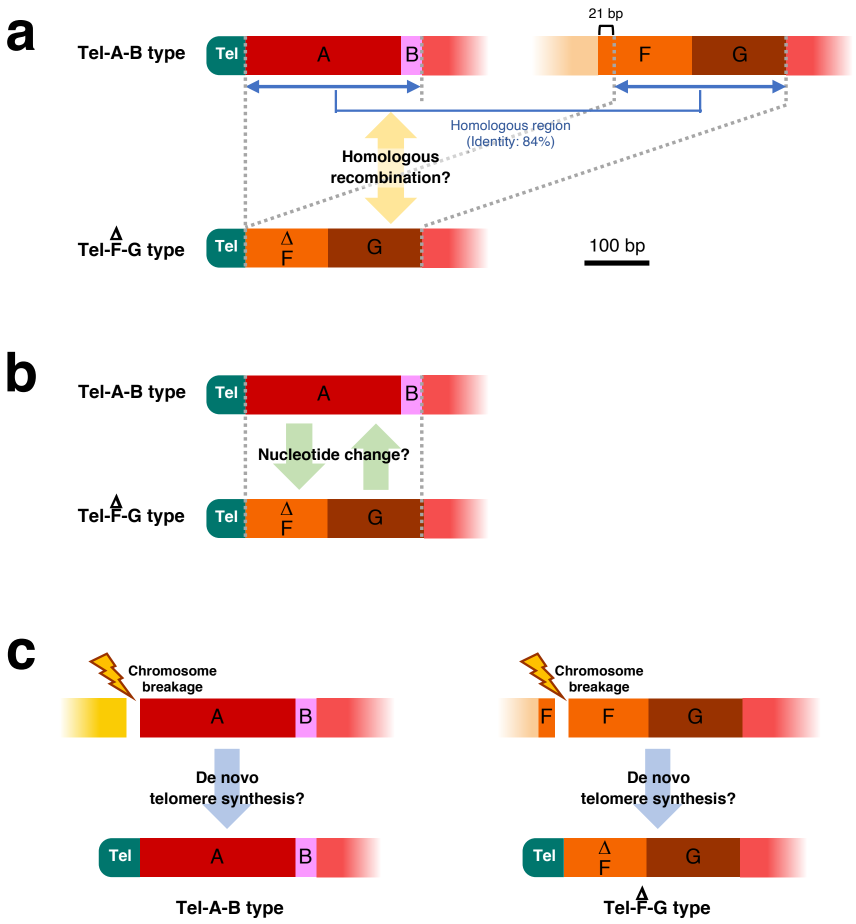

Supplementary Fig. 6 Hypothetical models for the two types of chromosome ends.

(a) Homologous recombination occurred between the homologous sequences, A-B and ΔF-G, in the same or different subtelomeres, which resulted in formation of the two types of chromosome ends, Tel-A-B and Tel-ΔF-G.

(b) Mutations were introduced into either A-B or ΔF-G sequences, which produced the two types of sequences at chromosome ends.

(c) G-rich sequences at the ends of segments A and ΔF induced chromosome breakage and *de novo* telomere synthesis.

a

```
ΨSH1L-L         1 ATTGATTGCTTTAATTTCAAACATGTCCCCTTTAATAGTTGGGACTTTAATAATTATCCTATTGTCAGGA  70
ΨSH1L-R, ΨSH1R  1 ATTGATTGCTTTAATTTCAAACATGTCCCCTTTAATAGTTGGGACTTTAATAATTATCCTATTGTCAGGA  70
ΨSH2L, ΨSH2R    1 ATTGATTGCTTTAATTTCAAACATGTCCCCTTTAATAGTTGGGACTTTAATAATTATCCTATTGTCAGGA  70

               71 CTCGCAACTGCTTTTTATGTTACGTGGCAAGGCAGACTCATTTGTGCTGGTGTAGGGCTCATACTTGAAC 140
               71 CTCGCAACTGCTTTTTATGTTACGTGGCAAGGCAGACTCATTTGTGCTGGTGTAGGGCTCATACTTGAAC 140
               71 CTCGCAACTGCTTTTTATGTTACGTGGCAAGGCAGACTCATTTGTGCTGGTGTAGGGCTCATACTTGAAC 140

              141 AGGCTTATGAGGGTGGTCAGATGTTTAACACATTGATGGCACATTGCTTTGAAACGTACAATGGTGTTGA 210
              141 AGGCTTATGAGGGTGGTCAGATGTTTAACACATTGATGGCACATTGCTTTGAAACGTACAATGGTGTTGA 210
              141 AGGCTTATGAGGGTGGTCAGATGTTTAACACATTGATGGCACATTGCTTTGAAACGTACAATGGTGTTGA 210

              211 GAAAAGTGGAACGCAGTGTGTGGCCGATTGGCTTAAAGTAGGGCTTTTGGCTGTCACATTTGGGGCTGGA 280
              211 GAAAAGTGGAACGCAGTGTGTGGCCGATTGGCTTAAAGTAGGGCTTTTGGCTGTCACATTTGGGGCTGGA 280
              211 GAAAAGTGGAACGCAGTGTGTGGCCGATTGGCTTAAAGTAGGGCTTTTGGCTGTCACATTTGGGGCTGGA 280

              281 GGACCTAGATTGGTTAACACATTAGGTGGTACTTTTCTCACTTCTCCTACTGCAAAACGGAGCAATCTAT 350
              281 GGACCTAGATTGGTTAACACATTAGGTGGT---------TCTTCGCCTACTACAAAACGG-GTAATCTAT 340
              281 GGACCTAGATTGGTTAACACATTAGGTGGT---------TCTTCGCCTACTACAAAACGG-GTAATCTAT 340

              351 ATTGTGATGATTTTACTGGTGCTGATTACTTTAGCTGTGAACTTGAAACATTAAGGCCTTACACATTGAT 420
              341 ATTGTGATGATTTTACTGGTGCTGATTACTTTAGCTGTGAACTTGAAACATTAAGGCCTTACACATTGAT 410
              341 ATTGTGATGATTTTACTGGTGCTGATTACTTTAGCTGTGAACTTGAAACATTAAGGCCTTACACATTGAT 410

              421 GAGAAAGAGCCTGCCGTATGGTAATATACACGATGTATGGATTAACACAACCGACACTCATCAGATGATA 490
              411 GAGAAAGAGCCTGCCGTATGGTAATATACACGATGTATGGATTAACACAACCGACACTCATCAGATGATA 480
              411 GAGAAAGAGCCTGCCGTATGGTAATATACACGATGTATGGATTAACACAACCGACACTCATCAGATGATA 480

              491 GGTGTACATATGACATTGAATGGAACAGATATGATTCATTATTATAATAAAACTTATGTAATTAATTATT 560 (bp)
              481 GGTGTACATATGACATTGAATGGAACAGATATGATTCATTATTATAATAAAACTTATGTAATTAATTATT 550 (bp)
              481 GGTGTACATATGACATTGAATGGAACAGATATGATTCATTATTATAATAAAACTTACGTAATTAATTATT 550 (bp)
```

b

	ΨSH1L-R, ΨSH1R		*ΨSH2L, ΨSH2R*	
	Match /Total (bp)	Identity (%)	Match /Total (bp)	Identity (%)
ΨSH1L-L	546/560	97.50	545/560	97.32
ΨSH1L-R, ΨSH1R			549/550	99.82

c

```
ΩSH1L-L             1 GTTGTAAGGATTACGAGATGCCAACATCATAATGCTTTTCCTAAATAAATGAGACACCTCAGAACGTAAA  70
ΩSH1L-R             1 GTTGTAAGGATTACGAGATGCCAACATCATAATGCTTTTCCTAAATAAATGAGACACCTCAGAACGTAAA  70
ΩSH1R               1 GTTGTAAGGATTACGAGATGCCAACATCATAATGCTTTTCCTAAATAAATGAGACACCTCAGAACGTAAA  70
ΩSH2L-L, ΩSH2R-L    1 GTTGTAAGGATTACGAGATGCCAACATCATAATGCTTTTCCTAAATAAATGAGACACCTCAGAACGTAAA  70
ΩSH2L-R, ΩSH2R-R    1 GTTGTAAGGATTACGAGATGCCAACATCATAATGCTTTTCCTAAATAAATGAGACACCTCAGAACGTAAA  70

 71 ACATTCACCATTTTGG--TTTTTTTTTTAATTTTCTCGTTTATGGCTTAAAATATTTTAGTATATTTTCT 138
 71 ACATTCACCATTTTGG--TTTTTTTTTTAATTTTCTCGTTTATGGCTTAAAATATTTTAGTATATTTTCT 138
 71 ACATTCACCATTTTGG--TTTTTTTTTTAATTTTCTCGTTTATGGCTTAAAATATTTTAGTATATTTTCT 138
 71 ATATTCACCATTTTGG--TTTTTTTTTTAATTTTCTCGTTTATGGCTTAAAATATTTTAGTATATTTTCT 138
 71 ACATTCACCATTTTGGTTTTTTTTTTTTAATTTTCTCGTTTATGGCTTAAAATATTTTAGTATATTTTCT 140

139 TTTTTATTTTCCATAAACCACTCTTTTTAATTTTACTTTGGGGAAAAATACTAGTGAATCAGAAGTCATC 208
139 TTTTTATTTTCCATAAACCACTCTTTTTAATTTTACTTTGGGGAAAAATACTAGTGAATCAGAAGTCATC 208
139 TTTTTATTTTCCATAAACCACTCTTTTTAATTTTACTTTGGGGAAAAATACTAGTGAATCAGAAGTCATC 208
139 TTTTTATTTTCCATAAACCACTCTTTTTAATTTTACTTTGGGGAAAAATACTAGTGAATCAGAAGTCATC 208
141 TTTTTATTTTCCATAAACCACTCTTTTTAATTTTACTTTGGGGAAAAATACTAGTGAATCAGAAGTCATC 210

209 AAATTACAGTGCTGTCGTTCTATTCCAATGTCAAATCCAGAAAGCTTGAAAAAACAGGTTGAACCTCCTG 278
209 AAATTACAGTGCTGTCGTTCTATTCCAATGTCAAATCCAGAAAGCTTGAAAAAACAGGTTGAACCTCCTG 278
209 AAATTACAGTGCTGTCGTTCTATTCCAATGTCAAATCCAGAAAGCTTGAAAAAACAGGTTGAACCTCCTG 278
209 AAATTACAGTGCTGTCGTTCTATTCCAATGTCAAATCCAGAAAGCTTGAAAAAACAGGTTGAACCTCCTG 278
211 AAATTACAGTGCTGTCGTTCTATTCCAATGTCAAATCCAGAAAGCTTGAAAAAACAGGTTGAACCTCCTG 280

279 GTTACAATGAGTTATTTATGGTGGAAGATGTTTGTAATGTGGACCTAGAGCAGGGACTTGATTTGTGTAA 348
279 GTTACAATGAGTTATTTATGGTGGAAGATGTTTGTAATGTGGACCTAGAGCAGGGACTTGATTTGTGTAA 348
279 GTTACAATGAGTTATTTATGGTGGAAGATGTTTGTAATGTGGACCTAGAGCAGGGACTTGATTTGTGTAA 348
279 GTTACAATGAGTTATTTATGGTGGAAGATGTTTGTAATGTGGACCTAGAGCAGGGACTTGATTTGTGTAA 348
281 GTTACAATGAGTTATTTATGGTGGAAGATGTTTGTAATGTGGACCTAGAGCAGGGACTTGATTTGTGTAA 350

349 GCCTGAAAAGGTAAACAAACAATCTCAACGATCTCGACAATCCCGACAATCCCTCTTTACCAACACCATT 418
349 GCCTGAAAAGGTAAACAAACAATCTCAACGATCTCGACAATCCCGACAATCCCTCTTTACCAACACCATT 418
349 GCCTGAAAAGGTAAACAAACAATCTCAACGATCTCGACAATCCCGACAATCCCTCTTTACCAACACCATT 418
349 GCCTGAAAAGGTAAACAAACAATCTCAACGATCTCGACAATCCCGACAATCCCTCTTTACCAACACCATT 418
351 GCCTGAAAAGGTAAACAAACAATCTCAACGATCTCGACAATCCCGACAATCCCTCTTTACCAACACCATT 420

419 AAGCCTCAAAAAGACAAGATGAATATTAAAACAAATAAAATAAAAGAGTTTTTAAATGACCTTTTTACTG 488
419 AAGCCTCAAAAAGACAAGATGAATATTAAAACAAATAAAATAAAAGAGTTTTTAAATGACCTTTTTACTG 488
419 AAGCCTCAAAAAGACAAGATGAATATTAAAACAAATAAAATCAAAGAGTTTTTAAATGACCCTTTTACTG 488
419 AAGCCTCAAAAAGACAAGATGAATATTAAAACAAATAAAATAAAAGAGTTTTTAAATGACCTTTTTACTG 488
421 AAGCCTCAAAAAGACAAGATGAATATTAAAACAAATAAAATAAAAGAGTTTTTAAATGACCTTTTTACTG 490

489 AATTTTCTAAATTCCACAATAGCTATTATCCTAATGGAAGAATTTCTACTCAGGACAAATCTCGATGGGT 558
489 AATTTTCTAAATTCCACAATAGCTATTATCCTGATGGAAGGATTTCTACCCGCAGTAATTTTCGTTGGCC 558
489 AATTTTCTAAATTCCACAATAGCTATTATCCTGATGGAAGGATTTCTACCCGCAGTAATTTTCGTTGGCC 558
489 AATTTTCTAAATTCCACAATAGCTATTATCCTGATGGAAGAATTTCTACCCGCAGTAATTTTCGTTGGCC 558
491 AATTTTCTAAATTCCACAATAGCTATTATCCTGATGGAAGGATTTCTACCCGCAGTAATTTTCGTTGGCC 560

559 CTTGCTTATTATTTGGTCTATTATCACTATTTTAACAATAGACAAGAAATTTAAAATAAAAGAGTCATAT 628
559 CTTGCTTATTATTTGGTCTATTATCATTGTATTCGCAGTAGACAAAAAGTTCGAAGTCCAAAAGTTTCTT 628
559 CTTGCTTATTATTTGGTCTATTATCATTGTATTCGCAGTAGACAAAAAGTTCGAAGTCCAAAAGTTTCTT 628
559 CTTGCTTATTATTTGGTCTATTATCATTGTATTCGCAGTAGACAAAAAGTTCGAAGTCCAAAAGTTTCTT 628
561 CTTGCTTATTATTTGGTCTATTATCATTGTATTCGCAGTAGACAAAAAGTTCGAAGTCCAAAAGTTTCTT 630

629 TTAGAATGGATAGGTGAAAATCAGTCCCACAGTGAAATTTGGGGGCCTATTGTTATTTATGTTGGCTTAT 698
629 TCAATTTGGATAAATGAAAATCGGTTCTACAGTGAAATTTGGGTGCCTATTGCTATTTACGTTTGTTTGC 698
629 TCAATTTGGATAAATGAAAATCGGTTCTACAGTGAAATTTGGGTGCCTATTGCTATTTACGTTTGTTTGC 698
629 TCAATTTGGATAAATGAAAATCGGTTCTACAGTGAAATTTGGGTGCCTATTGCTATTTACGTTTGTTTGC 698
631 TCAATTTGGATAAATGAAAATCGGTTCTACAGTGAAATTTGGGTGCCTATTGCTATTTACGTTTGTTTGC 700

699 TCATACTCTTATTGTCTGCTTTTAACTGTACGTTTCCTTCACACTGAAGCTGTTTTT---TCTCTTTACT 765
699 TTGTTTTAATGTTACTTAGTTTAATTTGTATGTTTCCTTTATAGTGATGCTTTTTTTCTTTTTTTTTGCT 768
699 TTGTTTTAATGTTACTTAGTTTAATTTGTATGTTTCCTTTATAGTGATGCTTTTTTTCTTTTTTTTTGCT 768
699 TTGTTTTAATGTTACTTAGTTTAATTTGTATGTTTCCTTTATAGTGATGCTTTTTTTCTTTTTTTTTGCT 768
701 TTGTTTTAATGTTACTTAGTTTAATTTGTATGTTTCCTTTATAGTGATGCTTTTTTTCTTTTTTTTTGCT 770
```

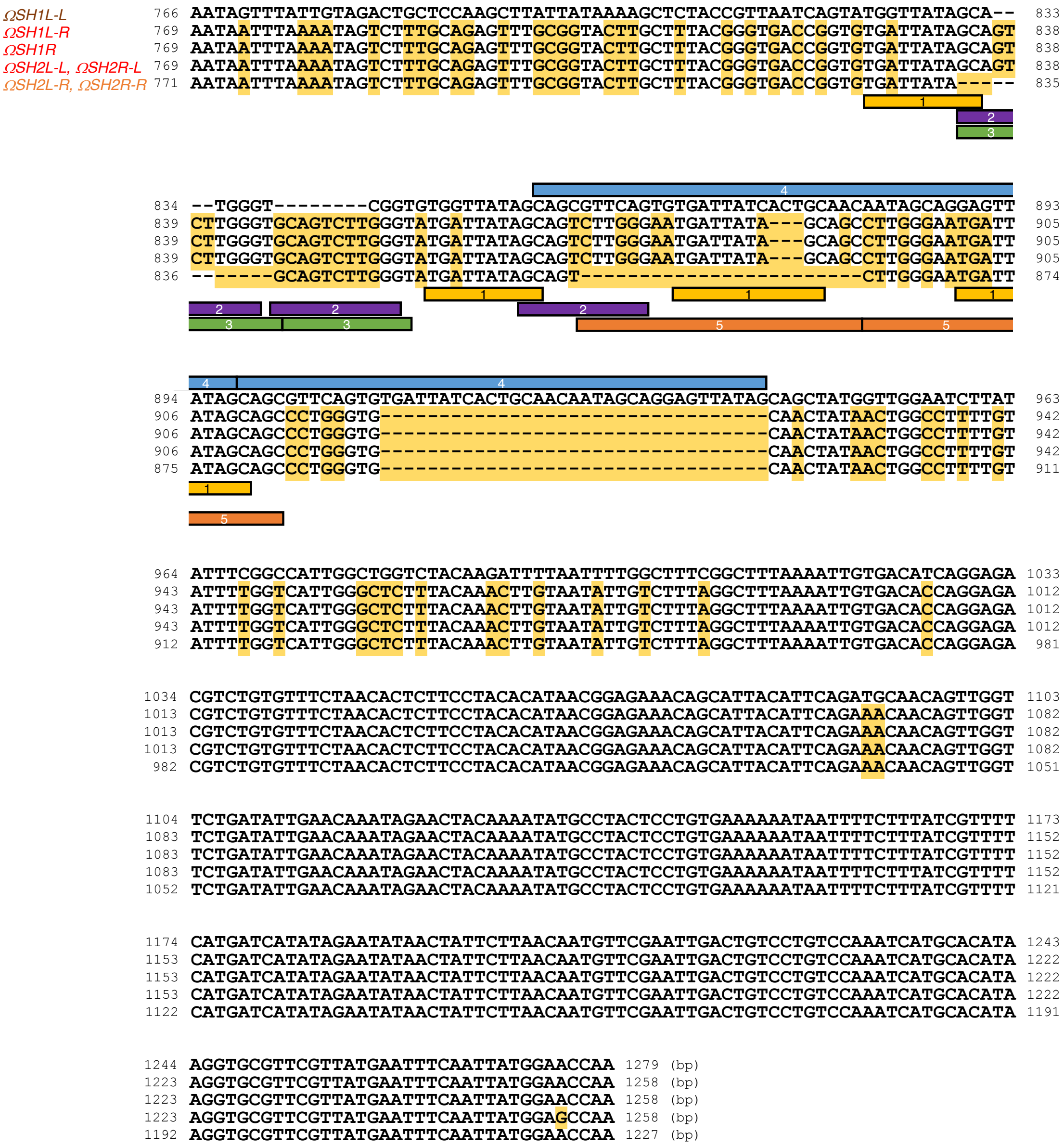

d

	ΩSH1L-R		ΩSH1R		ΩSH2L-L, ΩSH2R-L		ΩSH2L-R, ΩSH2R-R	
	Match /Total (bp)	Identity (%)	Match /Total (bp)	Identity (%)	Match /Total (bp)	Identity (%)	Match /Total (bp)	Identity (%)
ΩSH1L-L	1099/1294	84.93	1097/1294	84.78	1098/1294	84.85	1077/1292	83.36
ΩSH1L-R			1256/1258	99.84	1255/1258	99.76	1225/1260	97.22
ΩSH1R					1253/1258	99.60	1223/1260	97.06
ΩSH2L-L, ΩSH2R-L							1222/1260	96.98

Supplementary Fig. 7 Homologous sequences at the ends of changes in *SH-D* regions.

(a) Homologous *Ψ* sequences located at the ends of the 3.7 kb-change (purple and pink boxes in Fig. 5a–b). *ΨSH1L-L* (purple) and *ΨSH1L-R* (pink) are *Ψ* sequences on the left side and the right side of the 3.7 kb-insertion in *SH1L* in Fig. 5a–b, respectively; *ΨSH1R*, *ΨSH2L*, and *ΨSH2R* (pink) are *Ψ* sequences in *SH1R*, *SH2L*, and *SH2R*, respectively. Differences in sequences between *ΨSH1L-L* and the others are highlighted in yellow. Note that sequences of the pairs, *ΨSH1L-R* and *ΨSH1R*, and *ΨSH2L* and *ΨSH2R* are 100% identical.

(b) Sequence identities between *Ψ* sequences in (a).

(c) Homologous *Ω* sequences at the ends of the 7.1 kb-change (brown, red, and orange boxes in Fig. 5a–b). *ΩSH1L-L* (brown) and *ΩSH1L-R* (red) are sequences on the left side and the right side of the 7.1 kb-insertion, respectively, in *SH1L* in Fig. 5a–b; *ΩSH1R* (red), that in *SH1R*; *ΩSH2L-L* (red) and *ΩSH2L-R* (orange) are on the left side and the right side of the insertion in *SH2L*, respectively; *ΩSH2R-L* (red) and *ΩSH2R-R* (orange) are on the left side and the right side of the insertion in *SH2R*, respectively. Note that the region containing multiple repeat units (boxes 1–5) shows high sequence variation. Differences in sequences between *ΩSH1L-L* and the others are highlighted in yellow. Note that sequences of the pairs, *ΩSH2L-L* and *ΩSH2R-L*, and *ΩSH2L-R* and *ΩSH2R-R* are 100% identical.

(d) Sequence identities between *Ω* sequences in (c).

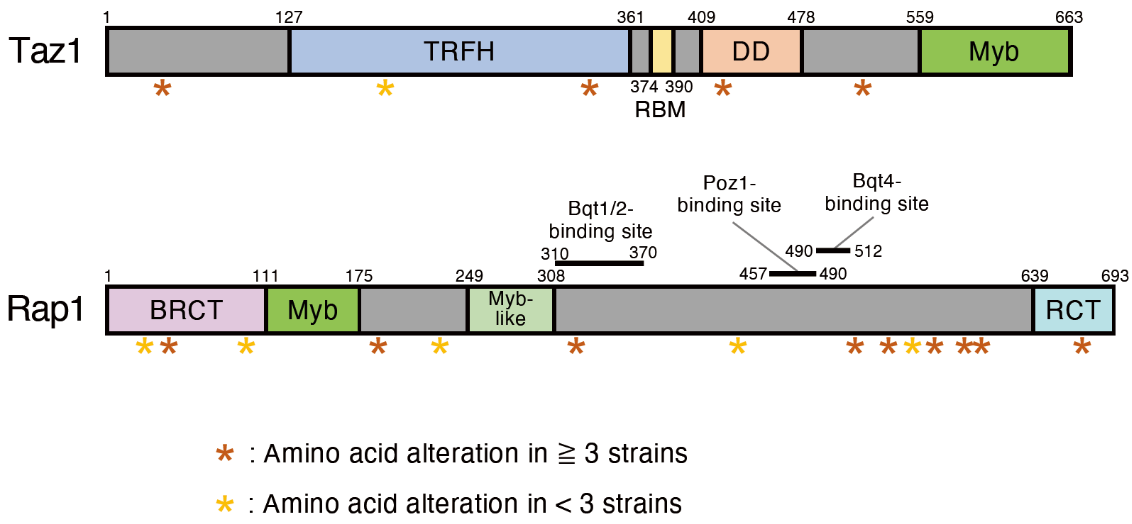

Supplementary Fig. 8 Variation of amino acid sequences of Taz1 and Rap1 in JB strains.

Amino acid alterations of Taz1 and Rap1 in JB strains compared with those in PomBase-*972*. Orange asterisk, amino acid alteration found in no less than three JB strains; yellow asterisk, that in less than three JB strains. TRFH, TRF-homology domain, RBM, Rap1-binding motif; DD, dimerization domain; Myb, Myb domain[3,4]. BRCT, BRCA1 C-terminal domain; Myb, Myb domain; Myb-like, Myb-like domain; RCT, Rap1 C-terminal domain; black bars, regions for interactions with Bqt1/2, Poz1 or Bqt4 proteins[3,5-8].

```
                        4525719                                                                     4525788
PomBase-tlh2            TATTCGTGATGAGAATCAACGTTGCAATTTTGTTTGTAGAAAATTGCCCTTCGAAAATTCGAAAATTTGG
tlh1 (972SD4[1L+])      TATTCGTGATGAGAATCAACGTTGCAATTTTGTTTGTAGAAAATTGCCCTTCGAAAATTCGAAAATTTGG
tlh3 (972SD4[1R+])      TATTCGTGATGAGAATCAACGTTGCAATTTTGTTTGTAGAAAATTGCCCTTCGAAAATTCGAAAATTTGG
tlh4 (972SD4[2L+])      TATTCGTGATGAGAATCAACGTTGCAATTTTGTTTGTAGAAAATTGCCCTTCGAAAATTCGAAAATTTGG
tlh2 (972SD4[2R+])      TATTCGTGATGAGAATCAACGTTGCAATTTTGTTTGTAGAAAATTGCCCTTCGAAAATTCGAAAATTTGG

                        4525789                                                                     4525858
                        ATTTGTATGCATTTTTTTTTGTTGATTTCTACGTCGATCATTATTTACTTATTCTATTTTTGTTTTTGATT
                        ATTTGTATGCATTTTTTTTTGTTGATTTCTACGTCGATCATTATTTACTTATTCTATTTTTGTTTTTGATT
                        ATTTGTATGCATTTTTTTTTGTTGATTTCTACGTCGATCATTATTTACTTATTCTATTTTTGTTTTTGATT
                        ATTTGTATGCATTTTTTTTTGTTGATTTCTACGTTGATCATTATTTACTTATTCTATTTTTGTTTTTGATT
                        ATTTGTATGCATTTTTTTTTGTTGATTTCTACGTCGATCATTATTTACTTATTCTATTTTTGTTTTTGATT

                        4525859                                                                     4525928
                        TTGTTTTTTTACTTGTTTGTTACGAAAAAAAGAGCCTATTTGCTGCTGCACAAAGCTTTTTGTTTGATTT
                        TTGTTTTTTTACTTGTTTGTTACGAAAAAAAGAGCTTATTTGCTGCTGCACAAAGCTTTTTGTTTGATTT
                        TTGTTTTTTTACTTGTTTGTTACGAAAAAAAGAGCCTATTTGCTGCTGCACAAAGCTTTTTGTTTGATTT
                        TTGTTTTTTTACTTGTTTGTTACGAAAAAAAGAGCTTATTTGCTGCTGCACAAAGCTTTTTGTTTGATTT
                        TTGTTTTTTTACTTGTTTGTTACGAAAAAAAGAGCTTATTTGCTGCTGCACAAAGCTTTTTGTTTGATTT

                        4525929                                                                     4525998
                        TTCATCCTACTTCTGTATTGGCAGTTTTCCTCATTTACAAAGACTAGCAACAGCCAGTCATTCATTTTCT
                        TTCATCCTACTTCTGTATTGGCAGTTTTCCTCATTTACAAAGACTAGCAACAGCCAGTCATTCATTTTCT
                        TTCATCCTACTTCTGTATTGGCAGTTTTCCTCATTTACAAAGACTAGCAACAGCCAGTCATTCATTTTCT
                        TTCATCCTACTTCTGTATTGGCAGTTTTCCTCATTTATAAAGACTAGCAACAGCCAGTCATTCATTTTCT
                        TTCATCCTACTTCTGTATTGGCAGTTTTCCTCATTTACAAAGACTAGCAACAGCCAGTCATTCATTTTCT

                        4525999                                                                     4526068
                        ACACGCTCTATTATTTCTTTTTTCATCACAATAAACTAACATGTCCGATTAGACTCTCAGATCCATTGCT
                        ACACGCTCTATTATTTCTTTTTTCATCACAATAAACTAACATGTCCGATTAGACTCTCAGATCCATTGCT
                        ACACGCTCTATTATTTCTTTTTTCATCACAATAAACTAACATGTCCGATTAGACTCTCAGATCCATTGCT
                        ACACGCTCTATTATTTCTTTTTTCATCACAATAAACTAACATGTCCGATTAGACTCTCAGATCCATTGCT
                        ACACGCTCTATTATTTCTTTTTTCATCACAATAAACTAACATGTCCGATTAGACTCTCAGATCCATTGCT

                        4526069                                                                     4526138
                        AGCAATTAATTGTCACTCTCCATTTTCCGTCTTTTCCTTTTTGATATTTTTTCTCTTTATATTATATTAT
                        AGCAATTAATTGTCACTCTCCATTTTCCGTCTTTTCCTTTTTGATATTTTTTCTCTTTATATTATATTAT
                        AGCAATTAATTGTCACTCTCCATTTTCCGTCTTTTCCTTTTTGATATTTTTTCTCTTTATATTATATTAT
                        AGCAATTAATTGTCACTCTCCATTTTCCGTCTTTTCCTTTTTGATATTTTTTCTCTTTATATTATATTAT
                        AGCAATTAATTGTCACTCTCCATTTTCCGTCTTTTCCTTTTTGATATTTTTTCTCTTTATATTATATTAT

                        4526139                                                                     4526206
                        ATTATATTCATTCTTCATTTTCTCTCTTGGCCATTATTTTTTTTTTTTTTTT--AATACCTCTGTGTCTC
                        ATTATATTCATTCTTCATTTTCTCTCTTGGCCATTATTTTTTTTTTTTTTTTTTAATACCTCTGTGTCTC
                        ATTATATTCATTCTTCATTTTCTCTCTTGGCCATTATTTTTTTTTTTTTTT---AATACCTCTGTGTCTC
                        ATTATATTCATTCTTCATTTTCTCTCTTGGCCATTATTTTTTTTTTTTTTTT--AATACCTCTGTGTCTC
                        ATTATATTCATTCTTCATTTTCTCTCTTGGCCATTATTTTTTTTTTTTTTTTT-AATACCTCTGTGTCTC

                        4526207     uM'                                                             4526275
                        TCGATTCAACATGGGTGAACTCAA-GAAATCTTGAAACAGAGGTATCATGAGTTGATTGATTGGAATGTT
                        TCGATTCAACATGGGTGAACTCAAGGAAATCTTGAAACAGAGGTATCATGAGTTGATTGATTGGAATGTT
                        TCGATTCAACATGGGTGAACTCAA-GAAATCTTGAAACAGAGGTATCATGAGTTGATTGATTGGAATGTT
                        TCGATTCAACATGGGTGAACTCAAGGAAATCTTGAAACAGAGGTATCATGAGTTGATTGATTGGAATGTT
                        TCGATTCAACATGGGTGAACTCAAGGAAATCTTGAAACAGAGGTATCATGAGTTGATTGATTGGAATGTT

                        4526276                                                                  uM 4526345
                        AAAGCTCCTCATGTTCCTCTTAGTAAACGATTAAAGCATTTTACATGGTCTTGGTTTGCGTGTACTATGG
                        AAAGCTCCTCATGTTCCTCTTAGTAAACGATTAAAGCATTTTACATGGTCTTGGTTTGCGTGTACTATGG
                        AAAGCTCCTCATGTTCCTCTTAGTAAACGATTAAAGCATTTTACATGGTCTTGGTTTGCGTGTACTATGG
                        AAAGCTCCTCATGTTCCTCTTAGTAAACGATTAAAGCATTTTACATGGTCTTGGTTTGCGTGTACTATGG
                        AAAGCTCCTCATGTTCCTCTTAGTAAACGATTAAAGCATTTTACATGGTCTTGGTTTGCGTGTACTATGG

                        4526346                                                                     4526415
                        CAACTGGTGGTGTTGGCTTGATCATTGGTTCTTTCCCCTTTCGTTTCTATGGTCTTAATACAATTGGCAA
                        CAACTGGTGGTGTTGGCTTGATCATTGGTTCTTTCCCCTTTCGTTTCTATGGTCTTAATACAATTGGCAA
                        CAACTGGTGGTGTTGGCTTGATCATTGGTTCTTTCCCCTTTCGTTTCTATGGTCTTAATACAATTGGCAA
                        CAACTGGTGGTGTTGGCTTGATCATTGGTTCTTTCCCCTTTCGTTTCTATGGTCTTAATACAATTGGCAA
                        CAACTGGTGGTGTTGGCTTGATCATTGGTTCTTTCCCCTTTCGTTTCTATGGTCTTAATACAATTGGCAA
```

```
4526416                                                                  4526485
AATTGTTTACATTCTTCAAATCGTTTTATTAACTCTCTTTGGATCGTGTATGCTTTTTCGTTTTATTAAA
AATTGTTTACATTCTTCAAATCGTTTTATTAACTCTCTTTGGATCGTGTATGCTTTTTCGTTTTATTAAA
AATTGTTTACATTCTTCAAATCGTTTTATTAACTCTCTTTGGATCGTGTATGCTTTTTCGTTTTATTAAA
AATTGTTTACATTCTTCAAATCGTTTTATTAACTCTCTTTGGATCGTGTATGCTTTTTCGTTTTATTAAA
AATTGTTTACATTCTTCAAATCGTTTTATTAACTCTCTTTGGATCGTGTATGCTTTTTCGTTTTATTAAA

4526486                                                                  4526555
TATCCTTCAACTATTAAAGATTCATGGAACCATCATTTGGAAAAGCTTTTCATTGCTACTTGTCTTCTTT
TATCCTTCAACTATTAAAGATTCATGGAACCATCATTTGGAAAAGCTTTTCATTGCTACTTGTCTTCTTT
TATCCTTCAACTATTAAAGATTCATGGAACCATCATTTGGAAAAGCTTTTCATTGCTACTTGTCTTCTTT
TATCCTTCAACTATTAAAGATTCATGGAACCATCATTTGGAAAAGCTTTTCATTGCTACTTGTCTTCTTT
TATCCTTCAACTATTAAAGATTCATGGAACCATCATTTGGAAAAGCTTTTCATTGCTACTTGTCTTCTTT

4526556                                                                  4526625
CGTTATCCACTTTCATCGACATGCTTGCCATATACGCCTATGCTGATACGGGCGAGTGGATGGTATGGGT
CGTTATCCACTTTCATCGACATGCTTGCCATATACGCCTATGCTGATACGGGCGAGTGGATGGTATGGGT
CGTTATCCACTTTCATCGACATGCTTGCCATATACGCCTATGCTGATACGGGCGAGTGGATGGTATGGGT
CGTTATCCACTTTCATCGACATGCTTGCCATATACGCCTATGCTGATACGGGCGAGTGGATGGTATGGGT
CGTTATCCACTTTCATCGACATGCTTGCCATATACGCCTATGCTGATACGGGCGAGTGGATGGTATGGGT

4526626                                                                  4526695
GATTCGAATTCTTTATTACATTTTTGTTGCAGTAGCTTTTATATACTGTGTAAGTGCTTTTTTCACAATT
GATTCGAATTCTTTATTACATTTTTGTTGCAGTAGCTTTTATATACTGTGTAAGTGCTTTTTTCACAATT
GATTCGAATTCTTTATTACATTTTTGTTGCAGTAGCTTTTATATACTGTGTAAGTGCTTTTTTCACAATT
GATTCGAATTCTTTATTACATTTTTGTTGCAGTAGCTTTTATATACTGTGTAAGTGCTTTTTTCACAATT
GATTCGAATTCTTTATTACATTTTTGTTGCAGTAGCTTTTATATACTGTGTAAGTGCTTTTTTCACAATT

4526696                                                                  4526765
TTCAACAATCATGTATATACCATTGAAACCGCATCTCCTGCTTGGATTCTTCCCATTTTTCCTCCTATGA
TTCAACAATCATGTATATACCATTGAAACCGCATCTCCTGCTTGGATTCTTCCCATTTTTCCTCCTATGA
TTCAACAATCATGTATATACCATTGAAACCGCATCTCCTGCTTGGATTCTTCCCATTTTTCCTCCTATGA
TTCAACAATCATGTATATACCATTGAAACCGCATCTCCTGCTTGGATTCTTCCCATTTTTCCTCCTATGA
TTCAACAATCATGTATATACCATTGAAACCGCATCTCCTGCTTGGATTCTTCCCATTTTTCCTCCTATGA

4526766                     dM'                        T      dM'        4526835
TTTGTGGTGTTATTGCTGGCGCTGTCAATGCTACACAACCTGCTCGTCAATTAAAAAATATGGTTATCTT
TTTGTGGTGTTATTGCTGGCGCTGTCAATGCTACACAACCTGCTCGTCAATTATAAAATATGGTTATCTT
TTTGTGGTGTTATTGCTGGCGCTGTCAATGCTACACAACCTGCTCGTCAATTAAAAAATATGGTTATCTT
TTTGTGGTGTTATTGCTGGCGCTGTCAATGCTACACAACCTGCTCGTCAATTATAAAATATGGTTATCTT
TTTGTGGTGTTATTGCTGGCGCTGTCAATGCTACACAACCTGCTCGTCAATTATAAAATATGGTTATCTT

4526836                                               dM                 4526905
TGGTATCCTTTTTTTTTTTTTTTTTTTTTTTTTTCACATTTCTACTGCCATGGTCGTCGCTTCAGAAATT
TGGTATCCTTTTTTTTTTTTTTTTTTTTTTTT--CACATTTCTACTGCCATGGTCGTCGCTTCAGAAATT
TGGTATCCTTTTTTTTTTTTTTTTTTTTTTT---CACATTTCTACTGCCATGGTCGTCGCTTCAGAAATT
TGGTATCCTTTTTTTTTTTTTTTTTTTTTTTTTTCACATTTCTACTGCCATGGTCGTCGCTTCAGAAATT
TGGTATCCTTTTTTTTTTTTTTTTTTTTTT----CACATTTCTACTGCCATGGTCGTCGCTTCAGAAATT

4526906                                                                  4526975
GCTAAAGTCGCTTCAAAAACTGCTAGAGATATCGCCGGATGCTTTACTTGTCAATGTGGAACTCAATTTG
GCTAAAGTCGCTTCAAAAACTGCTAGAGATATCGCCGGATGCTTTACTTGTCAATGTGGAACTCAATTTG
GCTAAAGTCGCTTCAAAAACTGCTAGAGATATCGCCGGATGCTTTACTTGTCAATGTGGAACTCAATTTG
GCTAAAGTCGCTTCAAAAACTGCTAGAGATATCGCCGGATGCTTTACTTGTCAATGTGGAACTCAATTTG
GCTAAAGTCGCTTCAAAAACTGCTAGAGATATCGCCGGATGCTTTACTTGTCAATGTGGAACTCAATTTG

4526976                                                                  4527045
ATAATGTAGAAAGGATCGTTCAACATTTCAAGGAATGTCGATACAGAGACGAAACTTGTAAAGACGATGA
ATAATGTAGAAAGGATCGTTCAACATTTCAAGGAATGTCGATACAGAGACGAAACTTGTAAAGACGATGA
ATAATGTAGAAAGGATCGTTCAACATTTCAAGGAATGTCGATACAGAGACGAAACTTGTAAAGACGATGA
ATAATGTAGAAAGGATCGTTCAACATTTCAAGGAATGTCGATACAGAGACGAAACTTGTAAAGACGATGA
ATAATGTAGAAAGGATCGTTCAACATTTCAAGGAATGTCGATACAGAGACGAAACTTGTAAAGACGATGA

4527046                                                                  4527115
TATCGTCGTCTATGAACCCAGCTCTTTCGTTCAGGACGAGAAAAAGGATAAGCCAATCATCGTTGAGGCT
TATCGTCGTCTATGAACCCAGCTCTTTCGTTCAGGACGAGAAAAAGGATAAGCCAATCATCGTTGAGGCT
TATCGTCGTCTATGAACCCAGCTCTTTCGTTCAGGACGAGAAAAAGGATAAGCCAATCATCGTTGAGGCT
TATCGTCGTCTATGAACCCAGCTCTTTCGTTCAGGACGAGAAAAAGGATAAGCCAATCATCGTTGAGGCT
TATCGTCGTCTATGAACCCAGCTCTTTCGTTCAGGACGAGAAAAAGGATAAGCCAATCATCGTTGAGGCT
```

4527116 4527185
GCCAGTGAGGCTACCAGTGAGGAGGCTTGCAATTCTTCCAAGGAGCGTCAACTACCCGCTCTCTCTGCTC
GCCAGTGAGGCTACCAGTGAGGAGGCTTGCAATTCTTCCAAGGAGCGTCAACTACCCGCTCTCTCTGCTC
GCCAGTGAGGCTACCAGTGAGGAGGCTTGCAATTCTTCCAAGGAGCGTCAACTACCCGCTCTCTCTGCTC
GCCAGTGAGGCTACCAGTGAGGAGGCTTGCAATTCTTCCAAGGAGCGTCAACTACCCGCTCTCTCTGCTC
GCCAGTGAGGCTACCAGTGAGGAGGCTTGCAATTCTTCCAAGGAGCGTCAACTACCCGCTCTCTCTGCTC

4527186 4527255
TTTCTGCTCTCTCTACTCTCACTACGAGTGCAAATGACGACTTGTGGACTGCACGGCTAATTTGGCAAAG
TCTCTGCTCTCTCTACTCTCACTACGAGTGCAAATGACGACTTGTGGACTGCACGGCTAATTTGGCAAAG
TTTCTGCTCTCTCTACTCTCACTACGAGTGCAAATGACGACTTGTGGACTGCACGGCTAATTTGGCAAAG
TCTCTGCTCTCTCTACTCTCACTACGAGTGCAAATGACGACTTGTGGACTGCACGGCTAATTTGGCAAAG
TCTCTGCTCTCTCTACTCTCACTACGAGTGCAAATGACGACTTGTGGACTGCACGGCTAATTTGGCAAAG

4527256 4527325
TACAAATGACACCAAATTGGACAACAGCCCATCATCAAATTATACTGACTTGAATCACAAGTTGGCTAAT
TACAAATGACACCAAATTGGACAACAGCCCATCATCAAATTATACTGACTTGAATCACAAGTTGGCTAAT
TACAAATGACACCAAATTGGACAACAGCCCATCATCAAATTATACTGACTTGAATCACAAGTTGGCTAAT
TACAAATGACACCAAATTGGACAACAGCCCATCATCAAATTATACTGACTTGAATCACAAGTTGGCTAAT
TACAAATGACACCAAATTGGACAACAGCCCATCATCAAATTATACTGACTTGAATCACAAGTTGGCTAAT

4527326 4527395
TATGGACTCTCCATTTTGTCTATTCATGCTTTGATGTGCGTAGAATGTGAATGCTTGTTAAACGTGATTC
TATGGACTCTCCATTTTGTCTATTCATGCTTTGATGTGCGTAGAATGTGAATGCTTGTTAAACGTGATTC
TATGGACTCTCCATTTTGTCTATTCATGCTTTGATGTGCGTAGAATGTGAATGCTTGTTAAACGTGATTC
TATGGACTCTCCATTTTGTCTATTCATGCTTTGATGTGCGTAGAATGTGAATGCTTGTTAAACGTGATTC
TATGGACTCTCCATTTTGTCTATTCATGCTTTGATGTGCGTAGAATGTGAATGCTTGTTAAACGTGATTC

4527396 4527465
ACACTGCGCAACATATGCAAATTGTGCATAAACTAGAGTTAAACGAAGATCTTTTGTGGTTTCAAGAACT
ACACTGCGCAACATATGCAAATTGTGCATAAACTAGAGTTAAACGAAGATCTTTTGTGGTTTCAAGAACT
ACACTGCGCAACATATGCAAATTGTGCATAAACTAGAGTTAAACGAAGATCTTTTGTGGTTTCAAGAACT
ACACTGCGCAACATATGCAAATTGTGCATAAACTAGAGTTAAACGAAGATCTTTTGTGGTTTCAAGAACT
ACACTGCGCAACATATGCAAATTGTGCATAAACTAGAGTTAAACGAAGATCTTTTGTGGTTTCAAGAACT

4527466 4527535
AAGGACTTTAAAGTTAAAAAGTCCAACAAATGTTTTACAAACTCATTCTTCTCAAACCCATGTATACCCA
AAGGACTTTAAAGTTAAAAAGTCCAACAAATGTTTTACAAACTCATTCTTCTCAAACCCATGTATACCCA
AAGGACTTTAAAGTTAAAAAGTCCAACAAATGTTTTACAAACTCATTCTTCTCAAACCCATGTATACCCA
AAGGACTTTAAAGTTAAAAAGTCCAACAAATGTTTTACAAACTCATTCTTCTCAAACCCATGTATACCCA
AAGGACTTTAAAGTTAAAAAGTCCAACAAATGTTTTACAAACTCATTCTTCTCAAACCCATGTATACCCA

4527536 4527605
TACATCAGAGGACTACCAGTACTTTTAAATGGATATGAGTGTGTGCCGTGCACAAAAAAACGGAACAGGAT
TACATCAGAGGACTACCAGTACTTTTAAATGGATATGAGTGTGTGCCGTGCACAAAAAAACGGAACAGGAT
TACATCAGAGGACTACCAGTACTTTTAAATGGATATGAGTGTGTGCCGTGCACAAAAAAACGGAACAGGAT
TACATCAGAGGACTACCAGTACTTTTAAATGGATATGAGTGTGTGCCGTGCACAAAAAAACGGAACAGGAT
TACATCAGAGGACTACCAGTACTTTTAAATGGATATGAGTGTGTGCCGTGCACAAAAAAACGGAACAGGAT

4527606 4527675
TCGTACATGCAATCATGGATACCTTTCGCCATCATGTTCGACGTACTCATGGAAAAGTTATAAAATTGGA
TCGTACATGCAATCATGGATACCTTTCGCCATCATGTTCGACGTACTCATGGAAAAGTTATAAAATTGGA
TCGTACATGCAATCATGGATACCTTTCGCCATCATGTTCGACGTACTCATGGAAAAGTTATAAAATTGGA
TCGTACATGCAATCATGGATACCTTTCGCCATCATGTTCGACGTACTCATGGAAAAGTTATAAAATTGGA
TCGTACATGCAATCATGGATACCTTTCGCCATCATGTTCGACGTACTCATGGAAAAGTTATAAAATTGGA

4527676 4527745
AAATTGTATACGTCGAACAGCTTTGCAAACGGTTAAGAACAAGTATGCTCAACGATGTCAGTTCTTCAAA
AAATTGTATACGTCGAACAGCTTTGCAAACGGTTAAGAACAAGTATGCTCAACGATGTCAGTTCTTCAAA
AAATTGTATACGTCGAACAGCTTTGCAAACGGTTAAGAACAAGTATGCTCAACGATGTCAGTTCTTCAAA
AAATTGTATACGTCGAACAGCTTTGCAAACGGTTAAGAACAAGTATGCTCAACGATGTCAGTTCTTCAAA
AAATTGTATACGTCGAACAGCTTTGCAAACGGTTAAGAACAAGTATGCTCAACGATGTCAGTTCTTCAAA

4527746 4527815
GTCGATTATGTACCTTTGAATGGAGGAGAAGAAGAAGAAGAGGAGGAGGGGGAGGAGAAAGAAGATGCAC
GTCGATTATGTACCTTTGAATGGAGGAGAAGAAGAAGAAGAGGAGGAGGGGGAGGAGAAAGAAGATGCAC
GTCGATTATGTACCTTTGAATGGAGGAGAAGAAGAAGAAGAGGAGGAGGGGGAGGAGAAAGAAGATGCAC
GTCGATTATGTACCTTTGAATGGAGGAGAAGAAGAAGAAGAGGAGGAGGGGGAGGAGAAAGAAGATGCAC
GTCGATTATGTACCTTTGAATGGAGGAGAAGAAGAAGAAGAGGAGGAGGGGGAGGAGAAAGAAGATGCAC

4527816 4527885
AAAACATCAAGGAAAGAATGGTGGACTTTTGCTTTTCAAAATTTATGGAGAAGAATCAACAGCGGCGGGA
AAAACATCAAGGAAAGAATGGTGGACTTTTGCTTTTCAAAATTTATGGAGAAGAATCAACAGCGGCGGGA
AAAACATCAAGGAAAGAATGGTGGACTTTTGCTTTTCAAAATTTATGGAGAAGAATCAACAGCCGTGGGA
AAAACATCAAGGAAAGAATGGTGGACTTTTGCTTTTCAAAATTTATGGAGAAGAATCAACAGCGGCGGGA
AAAACATCAAGGAAAGAATGGTGGACTTTTGCTTTTCAAAATTTATGGAGAAGAATCAACAGCGGCGGGA

4527886 4527955
GCAGCAAGACAAAGGTGAAAATAAAAAACGACAAGATGATGTTGACCAAGCAACAGACAACAACACTAAC
GCAGCAAGACAAAGGTGAAAATAAAAAACGACAAGATGATGTTGACCAAGCAACAGACAACAACACTAAC
GCAGCAAGACAAAGGTGAAAATAAAAAACGACAAGATGATGTTGACCAAGCAACAGACAACAACACTAAC
GCAGCAAGACAAAGGTGAAAATAAAAAACGACAAGATGATGTTGACCAAGCAACAGACAACAACACTAAC
GCAGCAAGACAAAGGTGAAAATAAAAAACGACAAGATGATGTTGACCAAGCAACAGACAACAACACTAAC

4527956 4528025
ACTATTCTTGAAGATGATGAAAAAGATAACGATGAAGAGGAAGAGGAAGAGATTGTCAATGCACGAGAGA
ACTATTCTTGAAGATGATGAAAAAGATAACGATGAAGAGGAAGAGGAAGAGATTGTCAATGCACGAGAGA
ACTATTCTTGAAGATGATGAAAAAGATAACGATGAAGAGGAAGAGGAAGAGATTGTCAATGCACGAGAGA
ACTATTCTTGAAGATGATGAAAAAGATAACGATGAAGAGGAAGAGGAAGAGATTGTCAATGCACGAGAGA
ACTATTCTTGAAGATGATGAAAAAGATAACGATGAAGAGGAAGAGGAAGAGATTGTCAATGCACGAGAGA

4528026 4528095
AAAATCTTTTAAATCAACAATTCAATTGGACGGCAATTGTGAAAAAACTTGGAGAAAATTGGGATCAGCT
AAAATCTTTTAAATCAACAATTCAATTGGACGGCAATTGTGAAAAAACTTGGAGAAAATTGGGATCAGCT
AAAATCTTTTAAATCAACAATTCAATTGGACGGCAATTGTGAAAAAACTTGGAGAAAATTGGGATCAGCT
AAAATCTTTTAAATCAACAATTCAATTGGACGGCAATTGTGAAAAAACTTGGAGAAAATTGGGATCAGCT
AAAATCTTTTAAATCAACAATTCAATTGGACGGCAATTGTGAAAAAACTTGGAGAAAATTGGGATCAGCT

4528096 4528165
GGTTCGATTCGAATATACGAATGGAATTGTAACTTTGGATACCATTGTAAACCAGTTGATTCGTTATTAC
GGTTCGATTCGAATATACGAATGGAATTGTAACTTTGGATACCATTGTAAACCAGTTGATTCGTTATTAC
GGTTCGATTCGAATATACGAATGGAATTGTAACTTTGGATACCATTGTAAACCAGTTGATTCGTTATTAC
GGTTCGATTCGAATATACGAATGGAATTGTAACTTTGGATACCATTGTAAACCAGTTGATTCGTTATTAC
GGTTCGATTCGAATATACGAATGGAATTGTAACTTTGGATACCATTGTAAACCAGTTGATTCGTTATTAC

4528166 4528235
TATCGAGGATTCCGTCATCTAAGTGGAATGACCATGGGCATGAGACGAATGTTTACTCAAGGAGGAAGCT
TATCGAGGATTCCGTCATCTAAGTGGAATGACCATGGGCATGAGACGAATGTTTACTCAAGGAGGAAGCT
TATCGAGGATTCCGTCATCTAAGTGGAATGACCATGGGCATGAGACGAATGTTTACTCAAGGAGGAAGCT
TATCGAGGATTCCGTCATCTAAGTGGAATGACCATGGGCATGAGACGAATGTTTACTCAAGGAGGAAGCT
TATCGAGGATTCCGTCATCTAAGTGGAATGACCATGGGCATGAGACGAATGTTTACTCAAGGAGGAAGCT

4528236 4528305
ATAGTGCACAAGAGCGAGGACTTTGTCGCTTAGAGCAAAAGGATACAGTTGTGAGATATGCTCAAAGTGC
ATAGTGCACAAGAGCGAGGACTTTGTCGCTTAGAGCAAAAGGATACAGTTGTGAGATATGCTCAAAGTGC
ATAGTGCACAAGAGCGAGGACTTTGTCGCTTAGAGCAAAAGGATACAGTTGTGAGATATGCTCAAAGTGC
ATAGTGCACAAGAGCGAGGACTTTGTCGCTTAGAGCAAAAGGATACAGTTGTGAGATATGCTCAAAGTGC
ATAGTGCACAAGAGCGAGGACTTTGTCGCTTAGAGCAAAAGGATACAGTTGTGAGATATGCTCAAAGTGC

4528306 4528375
AGCACTATACCTAATATTTCTGTTACGTCGTCCATCAGCGGACTCGGGGATAAGAAGACACTTGGAAGCA
AGCACTATACCTAATATTTCTGTTACGTCGTCCATCAGCGGACTCGGGGATAAGAAGACACTTGGAAGCA
AGCACTATACCTAATATTTCTGTTACGTCGTCCATCAGCGGACTCGGGGATAAGAAGACACTTGGAAGCA
AGCACTATACCTAATATTTCTGTTACGTCGTCCATCAGCGGACTCGGGGATAAGAAGACACTTGGAAGCA
AGCACTATACCTAATATTTCTGTTACGTCGTCCATCAGCGGACTCGGGGATAAGAAGACACTTGGAAGCA

4528376 4528445
ATGTGTGGAGCAACAGTGGAGAGAAAAGAAGGTGGCAGTAACAGCAGCAGTAACATCAGCAACGTCGCCA
ATGTGTGGAGCAACAGTGGAGAGAAAAGAAGGTGGCAGTAACAGCAGCAGTAACATCAGCAACGTCGCCA
ATGTGTGGAGCAACAGTGGAGAGAAAAGAAGGTGGCAGTAACAGCAGCAGTAACATCAGCAACGTCGCCA
ATGTGTGGAGCAACAGTGGAGAGAAAAGAAGGTGGCAGTAACAGCAGCAGTAACATCAGCAACGTCGCCA
ATGTGTGGAGCAACAGTGGAGAGAAAAGAAGGTGGCAGTAACAGCAGCAGTAACATCAGCAACGTCGCCA

4528446 4528509
ACTTTGATAGTGCTGAAGACGACAATGACAATGACAATGACAATGACA------GAGACAGTAACAATAA
ACTTTGATAGTGCTGAAGACGACAATGACAATGACAATGACAATGACAATGACAGAGACAGTAACAATAA
ACTTTGATAGTGCTGAAGACGACAATGACAATGACAATGACAATGACA------GAGACAGTAACAATAA
ACTTTGATAGTGCTGAAGACGACAATGACAATGACAATGACAATGACA------GAGACAGTAACAATAA
ACTTTGATAGTGCTGAAGACGACAATGACAATGACAATGACAATGACA------GAGACAGTAACAATAA

4528510 4528579

CAATAACAATAACAACACCAATACTGACGATGATGATAAGCTGGCATACTTGGAACTGCACGAGGCATTA
CAATAACAATAACAACACCAATACTGACGATGATGATAAGCTGGCATACTTGGAACTGCACGAGGCATTA
CAATAACAATAACAACACCAATACTGACGATGATGATAAGCTGGCATACTTGGAACTGCACGAGGCATTA
CAATAACAATAACAACACCAATACTGACGATGATGATAAGCTGGCATACTTGGAACTGCACGAGGCATTA
CAATAACAATAACAACACCAATACTGACGATGATGATAAGCTGGCATACTTGGAACTGCACGAGGCATTA

4528580 4528649

AAATTGGCTTTTCTGCAACAATACGATTTTTCTAAAAATGTTCAAGACTTGGAAATCATGGAGTTTCTTG
AAATTGGCTTTTCTGCAACAATACGATTTTTCTAAAAATGTTCAAGACTTGGAAATCATGGAGTTTCTTG
AAATTGGCTTTTCTGCAACAATACGATTTTTCTAAAAATGTTCAAGACTTGGAAATCATGGAGTTTCTTG
AAATTGGCTTTTCTGCAACAATACGATTTTTCTAAAAATGTTCAAGACTTGGAAATCATGGAGTTTCTTG
AAATTGGCTTTTCTGCAACAATACGATTTTTCTAAAAATGTTCAAGACTTGGAAATCATGGAGTTTCTTG

4528650 4528719

CATGCATGTCTTTGCATAAAGATGGTACTTCAAAATATGCCTATGAAATCTCTGCCTGCTTTGCACCCCT
CATGCATGTCTTTGCATAAAGATGGTACTTCAAAATATGCCTATGAAATCTCTGCCTGCTTTGCACCCCT
CATGCATGTCTTTGCATAAAGATGGTACTTCAAAATATGCCTATGAAATCTCTGCCTGCTTTGCACCCCT
CATGCATGTCTTTGCATAAAGATGGTACTTCAAAATATGCCTATGAAATCTCTGCCTGCTTTGCACCCCT
CATGCATGTCTTTGCATAAAGATGGTACTTCAAAATATGCCTATGAAATCTCTGCCTGCTTTGCACCCCT

4528720 4528789

AATATACACGTGTCGTCTTGTAGCAGCATGTGAGTTACAACGATTGATCGATGAAAAACAAATCGACTTG
AATATACACGTGTCGTCTTGTAGCAGCATGTGAGTTACAACGATTGATCGATGAAAAACAAATCGACTTG
AATATACACGTGTCGTCTTGTAGCAGCATGTGAGTTACAACGATTGATCGATGAAAAACAAATCGACTTG
AATATACACGTGTCGTCTTGTAGCAGCATGTGAGTTACAACGATTGATCGATGAAAAACAAATCGACTTG
AATATACACGTGTCGTCTTGTAGCAGCATGTGAGTTACAACGATTGATCGATGAAAAACAAATCGACTTG

4528790 4528859

TTGTCCATTCCATCGTTTCAAACCGCTGGTTCTATTGCTTATGCTCATGTGTTTTGCTTTATCACGCTTG
TTGTCCATTCCATCGTTTCAAACCGCTGGTTCTATTGCTTATGCTCATGTGTTTTGCTTTATCACGCTTG
TTGTCCATTCCATCGTTTCAAACCGCTGGTTCTATTGCTTATGCTCATGTGTTTTGCTTTATCACGCTTG
TTGTCCATTCCATCGTTTCAAACCGCTGGTTCTATTGCTTATGCTCATGTGTTTTGCTTTATCACGCTTG
TTGTCCATTCCATCGTTTCAAACCGCTGGTTCTATTGCTTATGCTCATGTGTTTTGCTTTATCACGCTTG

4528860 4528929

GTCAAAGAAATTTATATGATGTTCTTTACGAAACCCAAAAAGTCGTTAGGGATATCATTCGAACGGAAGG
GTCAAAGAAATTTATATGATGTTCTTTACGAAACCCAAAAAGTCGTTAGGGATATCATTCGAACGGAAGG
GTCAAAGAAATTTATATGATGTTCTTTACGAAACCCAAAAAGTCGTTAGGGATATCATTCGAACGGAAGG
GTCAAAGAAATTTATATGATGTTCTTTACGAAACCCAAAAAGTCGTTAGGGATATCATTCGAACGGAAGG
GTCAAAGAAATTTATATGATGTTCTTTACGAAACCCAAAAAGTCGTTAGGGATATCATTCGAACGGAAGG

4528930 4528999

GTATGCAAATACACTGCAAGGACTAAGCCCAAGTACCGTATTGTTTCAGCCACGAAGTAATTCTATGTAC
GTATGCAAATACACTGCAAGGACTAAGCCCAAGTACCGTATTGTTTCAGCCACGAAGTAATTCTATGTAC
GTATGCAAATACACTGCAAGGACTAAGCCCAAGTACCGTATTGTTTCAGCCACGAAGTAATTCTATGTAC
GTATGCAAATACACTGCAAGGACTAAGCCCAAGTACCGTATTGTTTCAGCCACGAAGTAATTCTATGTAC
GTATGCAAATACACTGCAAGGACTAAGCCCAAGTACCGTATTGTTTCAGCCACGAAGTAATTCTATGTAC

4529000 4529069

CCGTGCATTGGAGATGCATTCAATAACATGGTGCGATTGGACTTGAGTGAGCTGACAGCTCTGTATGAGG
CCGTGCATTGGAGATGCATTCAATAACATGGTGCGATTGGACTTGAGTGAGCTGACAGCTCTGTATGAGG
CCGTGCATTGGAGATGCATTCAATAACATGGTGCGATTGGACTTGAGTGAGCTGACAGCTCTGTATGAGG
CCGTGCATTGGAGATGCATTCAATAACATGGTGCGATTGGACTTGAGTGAGCTGACAGCTCTGTATGAGG
CCGTGCATTGGAGATGCATTCAATAACATGGTGCGATTGGACTTGAGTGAGCTGACAGCTCTGTATGAGG

4529070 4529139

GGATGTTTGCCAAAGTTCAGGACTTGTTAAAAGAGCTATGTTTTGATATGAATGTGGAAAAACTATTACC
GGATGTTTGCCAAAGTTCAGGACTTGTTAAAAGAGCTATGTTTTGATATGAATGTGGAAAAACTATTACC
GGATGTTTGCCAAAGTTCAGGACTTGTTAAAAGAGCTATGTTTTGATATGAATGTGGAAAAACTATTACC
GGATGTTTGCCAAAGTTCAGGACTTGTTAAAAGAGCTATGTTTTGACATGAATGTGGAAAAACTATTACC
GGATGTTTGCCAAAGTTCAGGACTTGTTAAAAGAGCTATGTTTTGACATGAATGTGGAAAAACTATTACC

4529140 4529209

CATTTCGTTACTAAGGTCAATTGGAGATGACATAAACAATAGTAAATTGGGATATTCATTCTTTAAAGAG
CATTTCGTTACTAAGGTCAATTGGAGATGACATAAACAATAGTAAATTGGGATATTCATTCTTTAAAGAG
CATTTCGTTACTAAGGTCAATTGGAGATGACATAAACAATAGTAAATTGGGATATTCATTCTTTAAAGAG
CATTTCGTTACTAAGGTCAATTGGAGATGACATAAACAATAGTAAATTGGGATATTCATTCTTTAAAGAG
CATTTCGTTACTAAGGTCAATTGGAGATGACATAAACAATAGTAAATTGGGATATTCATTCTTTAAAGAG

4529210 4529279

TCGATAGAGATACGTTCATCTCATAGTGTGCTGTTACGAACAATACTTAAGAACAGTGAACTTTGTCATC
TCGATAGAGATACGTTCATCTCATAGTGTGCTGTTACGAACAATACTTAAGAACAGTGAACTTTGTCATC
TCGATAGAGATACGTTCATCTCATAGTGTGCTGTTACGAACAATACTTAAGAACAGTGAACTTTGTCATC
TCGATAGAGATACGTTCATCTCATAGTGTGCTGTTACGAACAATACTTAAGAACAGTGAACTTTGTCATC
TCGATAGAGATACGTTCATCTCATAGTGTGCTGTTACGAACAATACTTAAGAACAGTGAACTTTGTCATC

4529280 4529349

GGTTTTTCCCATCGATGTCTAAGAAGGATCTGACAAAGTTGTTTGGTGGAGTAAGTGATCAGCAAAGAAA
GGTTTTTCCCATCGATGTCTAAGAAGGATCTGACAAAGTTGTTTGGTGGAGTAAGTGATCAGCAAAGAAA
GGTTTTTCCCATCGATGTCTAAGAAGGATCTGACAAAGTTGTTTGGTGGAGTAAGTGATCAGCAAAGAAA
GGTTTTTCCCATCGATGTCTAAGAAGGATCTGACAAAGTTGTTTGGTGGAGTAAGTGATCAGCAAAGAAA
GGTTTTTCCCATCGATGTCTAAGAAGGATCTGACAAAGTTGTTTGGTGGAGTAAGTGATCAGCAAAGAAA

4529350 4529395

TGAGTGCGACAACTATAGCAACCACTACAACGACAA------------------------CTCTAATGAT
TGAGTGCGACAACTATAGCAACCACTACAACGACAA------------------------CTCTAATGAT
TGAGTGCGACAACTATAGCAACCACTACAACGACAACGACAACGACAACGACAA------CTCTAATGAT
TGAGTGCGACAACTATAGCAACCACTACAACGACAACGACAACGACAACGACAACGACAACTCTAATGAT
TGAGTGCGACAACTATAGCAACCACTACAACGACAACGACAACGACAACGACAACGACAACTCTAATGAT

4529396 4529465

AATGACAATGATGTGTTTCTCAAATTACATTGGTCTAAATCTGCTATTAAAAAGTATGAGACAAAGGCAT
AATGACAATGATGTGTTTCTCAAATTACATTGGTCTAAATCTGCTATTAAAAAGTATGAGACAAAGGCAT
AATGACAATGATGTGTTTCTCAAATTACATTGGTCTAAATCTGCTATTAAAAAGTATGAGACAAAGGCAT
AATGACAATGATGTGTTTCTCAAATTACATTGGTCTAAATCTGCTATTAAAAAGTATGAGACAAAGGCAT
AATGACAATGATGTGTTTCTCAAATTACATTGGTCTAAATCTGCTATTAAAAAGTATGAGACAAAGGCAT

4529466 4529535

CTATCTTCAATGAGTTATTGTTTTGTCTAGTGTACATATCTGCTGGACAACCAGCCAGAGCACAAGAGAT
CTATCTTCAATGAGTTATTGTTTTGTCTAGTGTACATATCTGCTGGACAACCAGCCAGAGCACAAGAGAT
CTATCTTCAATGAGTTATTGTTTTGTCTAGTGTACATATCTGCTGGACAACCAGCCAGAGCACAAGAGAT
CTATCTTCAATGAGTTATTGTTTTGTCTAGTGTACATATCTGCTGGACAACCAGCCAGAGCACAAGAGAT
CTATCTTCAATGAGTTATTGTTTTGTCTAGTGTACATATCTGCTGGACAACCAGCCAGAGCACAAGAGAT

4529536 4529605

GGTGTATTGGACTTTGCGGAATGGCAAGTATAAGACTCGCGAATTGTATTTGATGTTTGGAAGGCTGATG
GGTGTATTGGACTTTGCGGAATGGCAAGTATAAGACTCGCGAATTGTATTTGATGTTTGGAAGGCTGATG
GGTGTATTGGACTTTGCGGAATGGCAAGTATAAGACTCGCGAATTGTATTTGATGTTTGGAAGGCTGATG
GGTGTATTGGACTTTGCGGAATGGCAAGTATAAGACTCGCGAATTGTATTTGATGTTTGGAAGGCTGATG
GGTGTATTGGACTTTGCGGAATGGCAAGTATAAGACTCGCGAATTGTATTTGATGTTTGGAAGGCTGATG

4529606 4529675

ATTTACAGCAGATACGATAAGACTCGTAATATGAAGTTTGCTGAAAAGCCAATCCCCAGGTTTCTTTCTG
ATTTACAGCAGATACGATAAGACTCGTAATATGAAGTTTGCTGAAAAGCCAATCCCCAGGTTTCTTTCTG
ATTTACAGCAGATACGATAAGACTCGTAATATGAAGTTTGCTGAAAAGCCAATCCCCAGGTTTCTTTCTG
ATTTACAGCAGATACGATAAGACTCGTAATATGAAGTTTGCTGAAAAGCCAATCCCCAGGTTTCTTTCTG
ATTTACAGCAGATACGATAAGACTCGTAATATGAAGTTTGCTGAAAAGCCAATCCCCAGGTTTCTTTCTG

4529676 4529745

AGCCGCTTTCCATTTTAGCACTTCGGTACTATGTTTTGGTTCGACCATTGGAAGCATTGATGAAGTATGT
AGCCGCTTTCCATTTTAGCACTTCGGTACTATGTTTTGGTTCGACCATTGGAAGCATTGATGAAGTATGT
AGCCGCTTTCCATTTTAGCACTTCGGTACTATGTTTTGGTTCGACCATTGGAAGCATTGATGAAGTATGT
AGCCGCTTTCCATTTTAGCACTTCGGTACTATGTTTTGGTTCGACCATTGGAAGCATTGATGAAGTATGT
AGCCGCTTTCCATTTTAGCACTTCGGTACTATGTTTTGGTTCGACCATTGGAAGCATTGATGAAGTATGT

4529746 4529815

GACAACCGCTGATAGGTCGAAAGTAGCTGTATACTTGGATTTCATGTTTGTGATTGCTGGCGAACGATTG
GACAACCGCTGATAGGTCGAAAGTAGCTGTATACTTGGATTTCATGTTTGTGATTGCTGGCGAACGATTG
GACAACCGCTGATAGGTCGAAAGTAGCTGTATACTTGGATTTCATGTTTGTGATTGCTGGCGAACGATTG
GACAACCGCTGATAGGTCGAAAGTAGCTGTATACTTGGATTTCATGTTTGTGATTGCTGGCGAACGATTG
GACAACCGCTGATAGGTCGAAAGTAGCTGTATACTTGGATTTCATGTTTGTGATTGCTGGCGAACGATTG

4529816 4529885

CAAAGAGATTTACCGTATCGAATTTTTCCAAAGGCCACCTACCAATGCATTCAAAAACCGTTGGGATTTC
CAAAGAGATTTACCGTATCGAATTTTTCCAAAGGCCACCTACCAATGCATTCAAAAACCGTTGGGATTTC
CAAAGAGATTTACCGTATCGAATTTTTCCAAAGGCCACCTACCAATGCATTCAAAAACCGTTGGGATTTC
CAAAGAGATTTACCGTATCGAATTTTTCCAAAGGCCACCTACCAATGCATTCAAAAACCGTTGGGATTTC
CAAAGAGATTTACCGTATCGAATTTTTCCAAAGGCCACCTACCAATGCATTCAAAAACCGTTGGGATTTC

4529886 4529955
GAAACTACAGGCACATTGCTCACTATTTTAAAGAAAAAAACATCGAGGAAGAAATGACGAGGGAATCATA
GAAACTACAGGCACATTGCTCACTATTTTAAAGAAAAAAACATCGAGGAAGAAATGACGAGGGAATCATA
GAAACTACAGGCACATTGCTCACTATTTTAAAGAAAAAAACATCGAGGAAGAAATGACGAGGGAATCATA
GAAACTACAGGCACATTGCTCACTATTTTAAAGAAAAAAACATCGAGGAAGAAATGACGAGGGAATCATA
GAAACTACAGGCACATTGCTCACTATTTTAAAGAAAAAAACATCGAGGAAGAAATGACGAGGGAATCATA

4529956 4530025
TTTCGATTTACAGGCTGGACATACACGAAACACAGCGCTCTACATCTATGGGCGCACTATGGACAACTTG
TTTCGATTTACAGGCTGGACATACACGAAACACAGCGCTCTACATCTATGGGCGCACTATGGACAACTTG
TTTCGATTTACAGGCTGGACATACACGAAACACAGCGCTCTACATCTATGGGCGCACTATGGACAACTTG
TTTCGATTTGCAGGCTGGACATACACGAAACACAGCGCTCTACATCTATGGGCGCACTATGGACAACTTG
TTTCGATTTGCAGGCTGGACATACACGAAACACAGCGCTCTACATCTATGGGCGCACTATGGACAACTTG

4530026 4530095
CATTATCTGCCGTCGGATTATTTCGCCAACTTTTTTCGTGCAAGCTATAAGTGGCAGGAACTATTACAGA
CATTATCTGCCGTCGGATTATTTCGCCAACTTTTTTCGTGCAAGCTATAAGTGGCAGGAACTATTACAGA
CATTATCTGCCGTCGGATTATTTCGCCAACTTTTTTCGTGCAAGCTATAAGTGGCAGGAACTATTACAGA
CATTATCTGCCGTCGGATTATTTCGCCAACTTTTTTCGTGCAAGCTATAAGTGGCAGGAACTATTACAGA
CATTATCTGCCGTCGGATTATTTCGCCAACTTTTTTCGTGCAAGCTATAAGTGGCAGGAACTATTACAGA

4530096 4530165
TTCGAGATAACCCGACCCATGGACTGTTGGTGGAAACAAAGCACCCATTCATCAAGCGAGTTGATCAATT
TTCGAGATAACCCGACCCATGGACTGTTGGTGGAAACAAAGCACCCATTCATCAAGCGAGTTGATCAATT
TTCGAGATAACCCGACCCATGGACTGTTGGTGGAAACAAAGCACCCATTCATCAAGCGAGTTGATCAATT
TTCGAGATAACCCGACCCATGGACTGTTGGTGGAAACAAAGCACCCATTCATCAAGCGAGTTGATCAATT
TTCGAGATAACCCGACCCATGGACTGTTGGTGGAAACAAAGCACCCATTCATCAAGCGAGTTGATCAATT

4530166 4530235
GGAGGAAGCGCTGAATGAGAAGCTGGCAAGGTTGGTAGGTGAACAAATGGTGGAGGGGGACAAGGAAAAG
GGAGGAAGCGCTGAATGAGAAGCTGGCAAGGTTGGTAGGTGAACAAATGGTGGAGGGGGACAAGGAAAAG
GGAGGAAGCGCTGAATGAGAAGCTGGCAAGGTTGGTAGGTGAACAAATGGTGGAGGGGGACAAGGAAAAG
GGAGGAAGCGCTGAATGAGAAGCTGGCAAGGTTGGTAGGTGAACAAATGGTGGAGGGGGACAAGGAAAAG
GGAGGAAGCGCTGAATGAGAAGCTGGCAAGGTTGGTAGGTGAACAAATGGTGGAGGGGGACAAGGAAAAG

4530236 4530305
GACAAGACAAATGAGGAGAAGAACAAGGACGAGGTAAAGGCTGAAATGACACAGCCCGTTGTAAATCAAG
GACAAGACAAATGAGGAGAAGAACAAGGACGAGGTAAAGGCTGAAATGACACAGCCCGTTGTAAATCAAG
GACAAGACAAATGAGGAGAAGAACAAGGACGAGGTAAAGGCTGAAATGACACAGCCCGTTGTAAATCAAG
GACAAGACAAATGAGGAGAAGAACAAGGACGAGGTAAAGGCTGAAATGACACAGCCCGTTGTAAATCAAG
GACAAGACAAATGAGGAGAAGAACAAGGACGAGGTAAAGGCTGAAATGACACAGCCCGTTGTAAATCAAG

4530306 4530375
ACTCTCATGACTTACAAGACCAATTGGCCACTACGCCCACCGCGCCCACCGCATTTCACTACCGCCCAGG
ACTCTCATGACTTACAAGACCAATTGGCCACTACGCCCACCGCGCCCACCGCATTTCACTACCGCCCAGG
ACTCTCATGACTTACAAGACCAATTGGCCACTACGCCCACCGCGCCCACCGCATTTCACTACCGCCCAGG
ACTCTCATGACTTACAAGACCAATTGGCCACTACGCCCACCGCGCCCACCGCATTTCACTACCGCCCAGG
ACTCTCATGACTTACAAGACCAATTGGCCACTACGCCCACCGCGCCCACCGCATTTCACTACCGCCCAGG

4530376 4530445
ACTTCTTCAACCATCTCAAACTTCTGTTCAACATTGTTGTTGGGCATTGTCTCAATATTATGGTCTCGAA
ACTTCTTCAACCATCTCAAACTTCTGTTCAACATTGTTGTTGGGCATTGTCTCAATATTATGGTCTCGAA
ACTTCTTCAACCATCTCAAACTTCTGTTCAACATTGTTGTTGGGCATTGTCTCAATATTATGGTCTCGAA
ACTTCTTCAACCATCTCAAACTTCTGTTCAACATTGTTGTTGGGCATTGTCTCAATATTATGGTCTCGAA
ACTTCTTCAACCATCTCAAACTTCTGTTCAACATTGTTGTTGGGCATTGTCTCAATATTATGGTCTCGAA

4530446 4530515
GCGAAATTCCGTTCGCTGAAACAATTTCAATCGGTTTATTTTTCTCTTTTAAATCGTATGAATTTGATTA
GCGAAATTCCGTTCGCTGAAACAATTTCAATCGGTTTATTTTTCTCTTTTAAATCGTATGAATTTGATTA
GCGAAATTCCGTTCGCTGAAACAATTTCAATCGGTTTATTTTTCTCTTTTAAATCGTATGAATTTGATTA
GCGAAATTCCGTTCGCTGAAACAATTTCAATCGGTTTATTTTTCTCTTTTAAATCGTATGAATTTGATTA
GCGAAATTCCGTTCGCTGAAACAATTTCAATCGGTTTATTTTTCTCTTTTAAATCGTATGAATTTGATTA

4530516 4530585
CCGTACTCCCAACTGGAGGTGGAAAGTCTTTGTCGTTTTTGATACCGGCGCTCATCGAAAAAAAAAGACA
CCGTACTCCCAACTGGAGGTGGAAAGTCTTTGTCGTTTTTGATACCGGCGCTCATCGAAAAAAAAAGACA
CCGTACTCCCAACTGGAGGTGGAAAGTCTTTGTCGTTTTTGATACCGGCGCTCATCGAAAAAAAAAGACA
CCGTACTCCCAACTGGAGGTGGAAAGTCTTTGTCGTTTTTGATACCGGCGCTCATCGAAAAAAAAAGACA
CCGTACTCCCAACTGGAGGTGGAAAGTCTTTGTCGTTTTTGATACCGGCGCTCATCGAAAAAAAAAGACA

4530586 4530655

AACCCCAGGAAAGGTGATGAATATGGTCACGCTGGTTCTAGTGCCCATGATGTCGTTACGGCAAGATATG
AACCCCAGGAAAGGTGATGAATATGGTCACGCTGGTTCTAGTGCCCATGATGTCGTTACGGCAAGATATG
AACCCCAGGAAAGGTGATGAATATGGTCACGCTGGTTCTAGTGCCCATGATGTCGTTACGGCAAGATATG
AACCCCAGGAAAGGTGATGAATATGGTCACGCTGGTTCTAGTGCCCATGATGTCGTTACGGCAAGATATG
AACCCCAGGAAAGGTGATGAATATGGTCACGCTGGTTCTAGTGCCCATGATGTCGTTACGGCAAGATATG

4530656 4530725

ATGCTCAGAGTGAATGAAAAGGGACTGCTTGTTTGTTCGGGGAATTGGACCGCATTCAAAGATGTACGAT
ATGCTCAGAGTGAATGAAAAGGGACTGCTTGTTTGTTCGGGGAATTGGACCGCATTCAAAGATGTACGAT
ATGCTCAGAGTGAATGAAAAGGGACTGCTTGTTTGTTCGGGGAATTGGACCGCATTCAAAGATGTACGAT
ATGCTCAGAGTGAATGAAAAGGGACTGCTTGTTTGTTCGGGGAATTGGACCGCATTCAAAGATGTACGAT
ATGCTCAGAGTGAATGAAAAGGGACTGCTTGTTTGTTCGGGGAATTGGACCGCATTCAAAGATGTACGAT

4530726 4530795

TGACTTTAGAGACGCAACTTCCCGATTTGTTTATCTTGACATACGAGTCAGCATTAACCAACAGTGGTCT
TGACTTTAGAGACGCAACTTCCCGATTTGTTTATCTTGACATACGAGTCAGCATTAACCAACAGTGGTCT
TGACTTTAGAGACGCAACTTCCCGATTTGTTTATCTTGACATACGAGTCAGCATTAACCAACAGTGGTCT
TGACTTTAGAGACGCAACTTCCCGATTTGTTTATCTTGACATACGAGTCAGCATTAACCAACAGTGGTCT
TGACTTTAGAGACGCAACTTCCCGATTTGTTTATCTTGACATACGAGTCAGCATTAACCAACAGTGGTCT

4530796 4530865

TCGGTTTTTTGAAAGTTTGGCAACACTTGGTCGTTTGGCACGAGTGGTAATTGATGAAGCACATTTGTTA
TCGGTTTTTTGAAAGTTTGGCAACACTTGGTCGTTTGGCACGAGTGGTAATTGATGAAGCACATTTGTTA
TTGGTTTTTTGAAAGTTTGGCAACACTTGGTCGTTTGGCACGAGTGGTAATTGATGAAGCACATTTGTTA
TCGGTTTTTTGAAAGTTTGGCAACACTTGGTCGTTTGGCACGAGTGGTAATTGATGAAGCACATTTGTTA
TCGGTTTTTTGAAAGTTTGGCAACACTTGGTCGTTTGGCACGAGTGGTAATTGATGAAGCACATTTGTTA

4530866 4530935

CTCACCAGTGGAGCATGGAGAACGGCTTTGTCGAGAGCATCGCGGTTGTCTGGCTTGTATGCACCATTGC
CTCACCAGTGGAGCATGGAGAACGGCTTTGTCGAGAGCATCGCGGTTGTCTGGCTTGTATGCACCATTGC
CTCACCAGTGGAGCATGGAGAACGGCTTTGTCGAGAGCATCGCGGTTGTCTGGCTTGTATGCACCATTGC
CTCACCAGTGGAGCATGGAGAACGGCTTTGTCGAGAGCATCGCGGTTGTCTGGCTTGTATGCACCATTGC
CTCACCAGTGGAGCATGGAGAACGGCTTTGTCGAGAGCATCGCGGTTGTCTGGCTTGTATGCACCATTGC

4530936 4531005

ACTTGTTGAGTGCCACCTTTCCCAGGCAACTAGAGATGGTTGCCAGACAAACGTTTTGTACAAACTTTTA
ACTTGTTGAGTGCCACCTTTCCCAGGCAACTAGAGATGGTTGCCAGACAAACGTTTTGTACAAACTTTTA
ACTTGTTGAGTGCCACCTTTCCCAGGCAACTAGAGATGGTTGCCAGACAAACGTTTTGTACAAACTTTTA
ACTTGTTGAGTGCCACCTTTCCCAGGCAACTAGAGATGGTTGCCAGACAAACGTTTTGTACAAACTTTTA
ACTTGTTGAGTGCCACCTTTCCCAGGCAACTAGAGATGGTTGCCAGACAAACGTTTTGTACAAACTTTTA

4531006 4531075

CGTTTTACGAGAAACGTCTACTGCACGGGAAAACATCTTTTACTTTTTGCATCCCTATGATAATACCGAG
CGTTTTACGAGAAACGTCTACTGCACGGGAAAACATCTTTTACTTTTTGCATCCCTATGATAATACCGAG
CGTTTTACGAGAAACGTCTACTGCACGGGAAAACATCTTTTACTTTTTGCATCCCTATGATAATACCGAG
CGTTTTACGAGAAACGTCTACTGCACGCGAAAACATCTTTTACTTTTTGCATCCCTATGATAATACCGAG
CGTTTTACGAGAAACGTCTACTGCACGCGAAAACATCTTTTACTTTTTGCATCCCTATGATAATACCGAG

4531076 4531145

TTTTTGTTGGACTTGCGAACGTTGATGAAACGAACAAAGGTCTTTGAAGGTGATGGACGTGCTATCATCT
TTTTTGTTGGACTTGCGAACGTTGATGAAACGAACAAAGGTCTTTGAAGGTGATGGACGTGCTATCATCT
TTTTTGTTGGACTTGCGAACGTTGATGAAACGAACAAAGGTCTTTGAAGGTGATGGACGTGCTATCATCT
TTTTTGTTGGACTTGCAAACGTTGATGAAACGAACAAAGGTCTTTGAAGGTGATGGACGTGCTATCATCT
TTTTTGTTGGACTTGCAAACGTTGATGAAACGAACAAAGGTCTTTGAAGGTGATGGACGTGCTATCATCT

4531146 4531215

TTTGTCGAACCAAAAAGGATGTCGAATACATTCATCGTCGTCTTCACCAGTCGGACTTGTTCGCTCACAC
TTTGTCGAACCAAAAAGGATGTCGAATACATTCATCGTCGTCTTCACCAGTCGGACTTGTTCGCTCACAC
TTTGTCGAACCAAAAAGGATGTCGAATACATTCATCGTCGTCTTCACCAGTCGGACTTGTTCGCTCACAC
TTTGTCGAACCAAAAAGGATGTCGAATACATTCATCGTCGTCTTCACCAGTCGGACTTGTTCGCTCACAC
TTTGTCGAACCAAAAAGGATGTCGAATACATTCATCGTCGTCTTCACCAGTCGGACTTGTTCGCTCACAC

4531216 4531285

CCATGTAACCATTTACACAGGAGATGTAAGCGACGAAGAACGACAAATGAACTTTGACGCGTTTCGAAAT
CCATGTAACCATTTACACAGGAGATGTAAGCGACGAAGAACGACAAATGAACTTTGACGCGTTTCGAAAT
CCATGTAACCATTTACACAGGAGATGTAAGCGACGAAGAACGACAAATGAACTTTGACGCGTTTCGAAAT
CCATGTAACCATTTACACAGGAGATGTAAGCGACGAAGAACGACAAATGAACTTTGACGCGTTTCGAAAT
CCATGTAACCATTTACACAGGAGATGTAAGCGACGAAGAACGACAAATGAACTTTGACGCGTTTCGAAAT

4531286 4531355

GCAAATGGGAAGACACGAATCATGATCGCTACCAAGGCATTCGGACTCGGTATCAACTATATGGGAGTGC
GCAAATGGGAAGACACGAATCATGATCGCTACCAAGGCATTCGGACTCGGTATCAACTATATGGGAGTGC
GCAAATGGGAAGACACGAATCATGATCGCTACCAAGGCATTCGGACTCGGTATCAACTATATGGGAGTGC
GCAAATGGGAAGACACGAATCATGATCGCTACCAAGGCATTCGGACTCGGTATCAACTATATGGGAGTGC
GCAAATGGGAAGACACGAATCATGATCGCTACCAAGGCATTCGGACTCGGTATCAACTATATGGGAGTGC

4531356 4531425

GTTTAGTAGTACACTATGGATTACCAGCTTCATCTATGGATTATGTACAGGAGACAGGTCGAGCTGGAAG
GTTTAGTAGTACACTATGGATTACCAGCTTCATCTATGGATTATGTACAGGAGACAGGTCGAGCTGGAAG
GTTTAGTAGTACACTATGGATTACCAGCTTCATCTATGGATTATGTACAGGAGACAGGTCGAGCTGGAAG
GTTTAGTAGTACACTATGGATTACCAGCTTCATCTATGGATTATGTACAGGAGACAGGCCGAGCTGGAAG
GTTTAGTAGTACACTATGGATTACCAGCTTCATCTATGGATTATGTACAGGAGACAGGCCGAGCTGGAAG

4531426 4531495

AGATGGCAAGTATGCGATTGCAGCATTGTTTTACGAGAAATATGATTCTACATGGTCGAGCTACGTAGAG
AGATGGCAAGTATGCGATTGCAGCATTGTTTTACGAGAAATATGATTCTACATGGTCGAGCTACGTAGAG
AGATGGCAAGTATGCGATTGCAGCATTGTTTTACGAGAAATATGATTCTACATGGTCGAGCTACGTGGAG
AGATGGCAAGTATGCGATTGCAGCATTGTTTTACGAGAAATATGATTCTACATGGTCGAGCTACGTAGAG
AGATGGCAAGTATGCGATTGCAGCATTGTTTTACGAGAAATATGATTCTACATGGTCGAGCTACGTAGAG

4531496 4531565

GATTCGATGAAAAACTTTCTTAATGATAATACGATGTGTGTTCGATCGTTTCTCGCAAGTGAAATGGATG
GATTCGATGAAAAACTTTCTTAATGATAATACGATGTGTGTTCGATCGTTTCTCGCAAGTGAAATGGATG
GATTCGATGAAAAACTTTCTTAATGATAATACGATGTGTGTTCGATCGTTTCTCGCAAGTGAAATGGATG
GATTCGATGAAAAACTTTCTTAATGATAATACGATGTGTGTTCGATCGTTTCTCGCAAGTGAAATGGATG
GATTCGATGAAAAACTTTCTTAATGATAATACGATGTGTGTTCGATCGTTTCTCGCAAGTGAAATGGATG

4531566 4531635

GCGAATGTGTATGTTGTGCATCGTTTGCTAACTGTGTTTACTGCTCAAGATGCTCAGATTCGTTACTTGG
GCGAATGTGTATGTTGTGCATCGTTTGCTAACTGTGTTTACTGCTCAAGATGCTCAGATTCGTTACTTGG
GCGAATGTGTATGTTGTGCATCGTTTGCTAACTGTGTTTACTGCTCAAGATGCTCAGATTCGTTACTTGG
GCGAATGTGTATGTTGTGCATCGTTTACTAACTGTGTTTACTGCTCAAGATGCTCAGATTCGTTACTTGG
GCGAATGTGTATGTTGTGCATCGTTTACTAACTGTGTTTACTGCTCAAGATGCTCAGATTCGTTACTTGG

4531636 4531705

TGAAGAATCAACTGTGTCTACGATGTATGGAGTGAAACCGACATTGCCAGAAACACCGAAACCAGCCATT
TGAAGAATCAACTGTGTCTACGATGTATGGAGTGAAACCGACATTGCCAGAAACACCGAAACCAGCCATT
TGAAGAATCAACTGTGTCTACGATGTATGGAGTGAAACCGACATTGCCAGAAACACCGAAACCAGCCATT
TGAAGAATCAACTGTGTCTACGATGTATGGAGTGAAACCGACATTGCCAGAAACACCGAAACCAGCCATT
TGAAGAATCAACTGTGTCTACGATGTATGGAGTGAAACCGACATTGCCAGAAACACCGAAACCAGCCATT

4531706 4531775

GCAACACATTCGCGTTATAATGCATCGTTTTCGTCTTCCCCCCCACCACAGCCAGGGAATAGCAGTGGTA
GCAACACATTCGCGTTATAATGCATCGTTTTCGTCTTCCCCCCCACCACAGCCAGGGAATAGCAGTGGTA
GCAACACATTCGCGTTATAATGCATCGTTTTCGTCTTCCCCCCCACCACAGCCAGGGAATAGCAGTGGTA
GCAACACATTCGCGTTATAATGCATCGTTTTCGTCTTCCCCCCTACCACAGCCAGGGAGTAGCAGTGGTA
GCAACACATTCGCGTTATAATGCATCGTTTTCGTCTTCCCCCCTACCACAGCCAGGGAGTAGCAGTGGTA

4531776 4531845

TGAGTGCTATGAACACTAACACTACTAGTACTACGCCAGTGTCTTTGTCGGAATTATCGGAAATCACTTT
TGAGTGCTATGAACACTAACACTACTAGTACTACGCCAGTGTCTTTGTCGGAATTATCGGAAATCACTTT
TGAGTGCTATGAACACTAACACTACTAGTACTACGCCAGTGTCTTTGTCGGAATTATCGGAAATCACTTT
TGAGTGCTATGAACACTAACACTACTAGTACTACGCCAGTGTCTTTGTCGGAATTATCGGAAATCACTTT
TGAGTGCTATGAACACTAACACTACTAGTACTACGCCAGTGTCTTTGTCGGAATTATCGGAAATCACTTT

4531846 4531915

GTTCCCTTCGTCTGTATCGCCAACATGGAAGAAAAGCTTTGGCAATGCTAATACGAATCTAAAGTATGGT
GTTCCCTTCGTCTGTATCGCCAACATGGAAGAAAAGCTTTGGCAATGCTAATACGAATCTAAAGTATGGT
GTTCCCTTCGTCTGTATCGCCAACATGGAAGAAAAGCTTTGGCAATGCTAATACGAATCTAAAGTATGGT
GTTCCCTTCGTCTGTATCGCCAACATGGAAGAAAAGCTTTGGCAATGCTAATACGAATCTAAAGTATGGT
GTTCCCTTCGTCTGTATCGCCAACATGGAAGAAAAGCTTTGGCAATGCTAATACGAATCTAAAGTATGGT

4531916 4531985

TTGGAAGACATGTCGCTCAGCCATCGTCGTGGTCATAAACGCACATACGATGAACATTTGAACAATGTAC
TTGGAAGACATGTCGCTCAGCCATCGTCGTGGTCATAAACGCACATACGATGAACATTTGAACAATGTAC
TTGGAAGACATGTCGCTCAGCCATCGTCGTGGTCATAAACGCACATACGATGAACATTTGAACAATGTAC
TTGGAAGACATGTCGCTCAGCCATCGTCGTGGTCATAAACGCACATACGATGAACATTTGAACAATGTAC
TTGGAAGACATGTCGCTCAGCCATCGTCGTGGTCATAAACGCACATACGATGAACATTTGAACAATGTAC

```
4531986                                                                  4532055
   AACAGGGCGTCAACCATGACATGAATCGTGTTCATGGTAGTGTTGGAGGTATGAGTGGTATTGTTGGTAT
   AACAGGGCGTCAACCATGACATGAATCGTGTTCATGGTAGTGTTGGAGGTATGAGTGGTATTGTTGGTAT
   AACAGGGCGTCAACCATGACATGAATCGTGTTCATGGTAGTGTTGGAGGTATGAGTGGTATTGTTGGTAT
   AACAGGGCGTCAACCATGACATGAATCGTGTTCATGGTAGTGTTGGAGGTATGAGTGGTATTGTTGGTAT
   AACAGGGCGTCAACCATGACATGAATCGTGTTCATGGTAGTGTTGGAGGTATGAGTGGTATTGTTGGTAT

4532056                                                                  4532119
   TGGTATTGGTATTGGTGATGGTGATGGTGATGG------TGATGTTGATTCTCGTACCATTCATTTCGCC
   TGGTATTGGTATTGGTGATGGTGATGGTGATGG------TGATGTTGATTCTCGTACCATTCATTTCGCC
   TGGTATTGGTATTGGTGATGGTGATGGTGATGGTGATGGTGATGTTGATTCTCGTACCATTCATTTCGCC
   TGGTATTGGTATTGGTGATGGTGATGGTGATGT------TGATGTTGATTCTCGTACCATTCATTTCGCC
   TGGTATTGGTATTGGTGATGGTGATGGTGATGT------TGATGTTGATTCTCGTACCATTCATTTCGCC

4532120                                                                  4532189
   GAGTATAAAAGTCGTGTGCAAGCCGTCAAAAAACAATGGGTAGACTCAACTGACATTTCAGCACAACTCG
   GAGTATAAAAGTCGTGTGCAAGCCGTCAAAAAACAATGGGTAGACTCAACTGACATTTCAGCACAACTCG
   GAGTATAAAAGTCGTGTGCAAGCCGTCAAAAAACAATGGGTAGACTCAACTGACATTTCAGCACAACTCG
   GAGTATAAAAGTCGTGTGCAAGCCGTCAAAAAACAATGGGTAGACTCAACTGACATTTCAGCACAACTCG
   GAGTATAAAAGTCGTGTGCAAGCCGTCAAAAAACAATGGGTAGACTCAACTGACATTTCAGCACAACTCG

4532190                                                                  4532259
   AGCGATTCTTTCGAGTGTATAAAGATGAATGTCTAAGTTGCACTCTTGGTAACCCAGACACAGAAATTCG
   AGCGATTCTTTCGAGTGTATAAAGATGAATGTCTAAGTTGCACTCTTGGTAACCCAGACACAGAAATTCG
   AGCGATTCTTTCGAGTGTATAAAGATGAATGTCTAAGTTGCACTCTTGGTAACCCAGACACAGAAATTCG
   AGCGATTCTTTCGAGTGTATAAAGATGAATGTCTAAGTTGCACTCTTGGTAACCCAGACACAGAAATTCG
   AGCGATTCTTTCGAGTGTATAAAGATGAATGTCTAAGTTGCACTCTTGGTAACCCAGACACAGAAATTCG

4532260                                                                  4532329
   TGCTCATACCGGGAAAGCATGCCCCGTACGCTTATCTACTTGTTATAAATGTGGCAAGGCCGACCACAAC
   TGCTCATACCGGGAAAGCATGCCCCGTACGCTTATCTACTTGTTATAAATGTGGCAAGGCCGACCACAAC
   TGCTCATACCGGGAAAGCATGCCCCGTACGCTTATCTACTTGTTATAAATGTGGCAAGGCCGACCACAAC
   TGCTCATACCGGGAAAGCATGCCCCGTACGCTTATCTACTTGTTATAAATGTGGCAAGGCCGACCACAAC
   TGCTCATACCGGGAAAGCATGCCCCGTACGCTTATCTACTTGTTATAAATGTGGCAAGGCCGACCACAAC

4532330                                                                  4532399
   TTGCGAGAATGTAAACTACGTATTCGATTTCAAGGACTTTGTTTATTTTGTGGTTTGACAAAGTTTGAAC
   TTGCGAGAATGTAAACTACGTATTCGATTTCAAGGACTTTGTTTATTTTGTGGTTTGACAAAGTTTGAAC
   TTGCGAGAATGTAAACTACGTATTCGATTTCAAGGACTTTGTTTATTTTGTGGTTTGACAAAGTTTGAAC
   TTGCGAGAATGTAAACTACGTATTCGATTTCAAGGACTTTGTTTATTTTGTGGTTTGACAAAGTTTGAAC
   TTGCGAGAATGTAAACTACGTATTCGATTTCAAGGACTTTGTTTATTTTGTGGTTTGACAAAGTTTGAAC

4532400                                                                  4532469
   ATGCCGACAGTGATATGGCTTACACTTCTGACTGTAGGTCATGGGCTAGAAAGGCAAACTTGATATCGTT
   ATGCCGACAGTGATATGGCTTACACTTCTGACTGTAGGTCATGGGCTAGAAAGGCAAACTTGATATCGTT
   ATGCCGACAGTGATATGGCTTACACTTCTGACTGTAGGTCATGGGCTAGAAAGGCAAACTTGATATCGTT
   ATGCCGACAGTGATATGGCTTACACTTCTGACTGTAGGTCATGGGCTAGAAAGGCAAACTTGATATCGTT
   ATGCCGACAGTGATATGGCTTACACTTCTGACTGTAGGTCATGGGCTAGAAAGGCAAACTTGATATCGTT

4532470                                                                  4532539
   AGTTTACTATGCTTGGAACAATGTACAATACCGAAGGACTATTGCAGATAAATTCTTACAAGGTGATGTA
   AGTTTACTATGCTTGGAACAATGTACAATACCGAAGGACTATTGCAGATAAATTCTTACAAGGTGATGTA
   AGTTTACTATGCTTGGAACAATGTACAATACCGAAGGACTATTGCAGATAAATTCTTACAAGGTGATGTA
   AGTTTACTATGCTTGGAACAATGTACAATACCGAAGGACTATTGCAGATAAATTCTTACAAGGTGATGTA
   AGTTTACTATGCTTGGAACAATGTACAATACCGAAGGACTATTGCAGATAAATTCTTACAAGGTGATGTA

4532540                                                                  4532609
   CGTGATCAAGCTTTTTATGGATTTGTGTGTTGCTCTACAACTACGAATTCTGCATTTGTTCTAGTTATTC
   CGTGATCAAGCTTTTTATGGATTTGTGTGTTGCTCTACAACTACGAATTCTGCATTTGTTCTAGTTATTC
   CGTGATCAAGCTTTTTATGGATTTGTGTGTTGCTCTACAACTACGAATTCTGCATTTGTTCTAGTTATTC
   CGTGATCAAGCTTTTTATGGATTTGTGTGTTGCTCTACAACTACGAATTCTGCATTTGTTCTAGTTATTC
   CGTGATCAAGCTTTTTATGGATTTGTGTGTTGCTCTACAACTACGAATTCTGCATTTGTTCTAGTTATTC

4532610                                T                                 4532679
   ATTACTTGTTGTCTGATGTTTTGCAAATCATGTAATTTTTTACTGATGCAAGACAACGTTTCATATCGTG
   ATTACTTGTTGTCTGATGTTTTGCAAATCATGTAATTTTTTACTGATGCAAGACAACGTTTCATATCGTG
   ATTACTTGTTGTCTGATGTTTTGCAAATCATGTAATTTTTTACTGATGCAAGACAACGTTTCATATCGTG
   ATTACTTGTTGTCTGATGTTTTGCAAATCATGTAATTTTTTACTGATGCAAGACAACGTTTCATATCGTG
   ATTACTTGTTGTCTGATGTTTTGCAAATCATGTAATTTTTTACTGATGCAAGACAACGTTTCATATCGTG
```

```
4532680                                                              4532749
TTGCACAAAAAGTAAGGGAGAGGCAGAAAAAGTCAGAGTGCTTTTCATTGTTAAAGTTACCATCCCCACC
TTGCACAAAAAGTAAGGGAGAGGCAGAAAAAGTCAGAGTGCTTTTCATTGTTAAAGTTACCATCCCCACC
TTGCACAAAAAGTAAGGGAGAGGCAGAAAAAGTCAGAGTGCTTTTCATTGTTAAAGTTACCATCCCCACC
TTGCACAAAAAGTAAGGGAGAGGCAGAAAAAGTCAGAGTGCTTTTCATTGTTAAAGTTACCATCCCCACC
TTGCACAAAAAGTAAGGGAGAGGCAGAAAAAGTCAGAGTGCTTTTCATTGTTAAAGTTACCATCCCCACC

4532750                                                              4532819
CCACCCAACGGATGTTGATGCAAAAGTACGAGTAAAATATAACAAGTTGGTGGAGATGATTGAATATGTT
CCACCCAACGGATGTTGATGCAAAAGTACGAGTAAAATATAACAAGTTGGTGGAGATGATTGAATATGTT
CCACCCAACGGATGTTGATGCAAAAGTACGAGTAAAATATAACAAGTTGGTGGAGATGATTGAATATGTT
CCACCCAACGGATGTTGATGCAAAAGTACGAGTAAAATATAACAAGTTGGTGGAGATGATTGAATATGTT
CCACCCAACGGATGTTGATGCAAAAGTACGAGTAAAATATAACAAGTTGGTGGAGATGATTGAATATGTT

4532820                                                              4532889
AGTAAATGTTGTAAATAGGATAAATTAACGAATGAAATGAAAATAAAGCTGAAATATGCATCAATGATTT
AGTAAATGTTGTAAATAGGATAAATTAACGAATGAAATGAAAATAAAGCTGAAATATGCATCAATGATTT
AGTAAATGTTGTAAATAGGATAAATTAACGAATGAAATGAAAATAAAGCTGAAATATGCATCAATGATTT
AGTAAATGTTGTAAATAGGATAAATTAACGAATGAAATGAAAATAAAGCTGAAATATGCATCAATGATTT
AGTAAATGTTGTAAATAGGATAAATTAACGAATGAAATGAAAATAAAGCTGAAATATGCATCAATGATTT

4532890                                                              4532959
TGAAATGAATGAAAAATAATGTTTTCGCAAAATAATTATTGAAATAGCTTGCATATTATATTATTAATTC
TGAAATGAATGAAAAATAATGTTTTCGCAAAATAATTATTGAAATAGCTTGCATATTATATTATTAATTC
TGAAATGAATGAAAAATAATGTTTTCGCAAAATAATTATTGAAATAGCTTGCATATTATATTATTAATTC
TGAAATGAATGAAAAATAATGTTTTCGCAAAATAATTATTGAAATAGCTTGCATATTATATTATTAATTC
TGAAATGAATGAAAAATAATGTTTTCGCAAAATAATTATTGAAATAGCTTGCATATTATATTATTAATTC

4532960                                                              4533029
AATCAACTATTTACAAAGACAAGTTTTTATAACTTTTCGAAAATCAAAAGTATTCAAAATTTTGTTTGCT
AATCAACTATTTACAAAGACAAGTTTTTATAACTTTTCGAAAATCAAAAGTATTCAAAATTTTGTTTGCT
AATCAACTATTTACAAAGACAAGTTTTTATAACTTTTCGAAAATCAAAAGTATTCAAAATTTTGTTTGCT
AATCAACTATTTACAAAGACAAGTTTTTATAACTTTTCGAAAATCAAAAGTATTCAAAATTTTGTTTGCT
AATCAACTATTTACAAAGACAAGTTTTTATAACTTTTCGAAAATCAAAAGTATTCAAAATTTTGTTTGCT

4533030                                                              4533099
TAGTATAAATGATATTCGTTGGCAAATTTATCTTATCTTTTGTATTCGCTTTACAAGTAAATTTGATAGA
TAGTATAAATGATATTCGTTGGCAAATTTATCTTATCTTTTGTATTCGCTTTACAAGTAAATTTGATAGA
TAGTATAAATGATATTCGTTGGCAAATTTATCTTATCTTTTGTATTCGCTTTACAAGTAAATTTGATAGA
TAGTATAAATGATATTCGTTGGCAAATTTATCTTATCTTTTGTATTCGCTTTACAAGTAAATTTGATAGA
TAGTATAAATGATATTCGTTGGCAAATTTATCTTATCTTTTGTATTCGCTTTACAAGTAAATTTGATAGA

4533100                                      4533144
CTTGTATGTTGTTTTCATATCGGAACATTCATGAAGGAGAAGGCA
CTTGTATGTTGTTTTCATATCGGAACATTCATGAAGGAGAAGGCA
CTTGTATGTTGTTTTCATATCGGAACATTCATGAAGGAGAAGGCA
CTTGTATGTTGTTTTCATATCGGAACATTCATGAAGGAGAAGGCA
CTTGTATGTTGTTTTCATATCGGAACATTCATGAAGGAGAAGGCA
```

Supplementary Fig. 9 Sequence alignment of the *tlh1–4* genes in *972SD4* with the PomBase-*tlh2* gene.

These sequences cover the same region as shown in Fig. 8b. Differences in sequences between PomBase-*tlh2* and the others are highlighted in yellow. Putative first Met codons, uM, uM′, dM, and dM′, and termination codons, T, are indicated in red and blue, respectively (also see Fig. 8b).

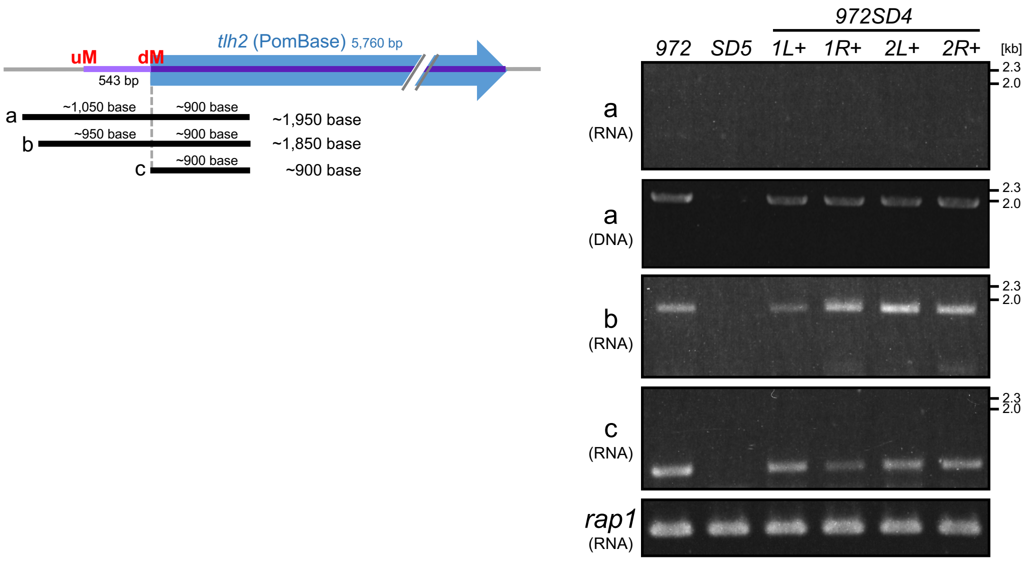

Supplementary Fig. 10 RNA expression from the upstream region of the *tlh* genes.

Expression of RNA from various regions (a–c) of the *tlh* loci and the *rap1* locus (control) was analyzed by RT-PCR in strains *972*, *SD5*, and *972SD4*. The PCR products were analyzed by gel electrophoresis. Genomic DNA of region (a) was also analyzed as a control of PCR (the second row). Note that primers used in PCRs for the a–c regions recognize all of the *tlh1–4* genes. These data were reproduced twice.

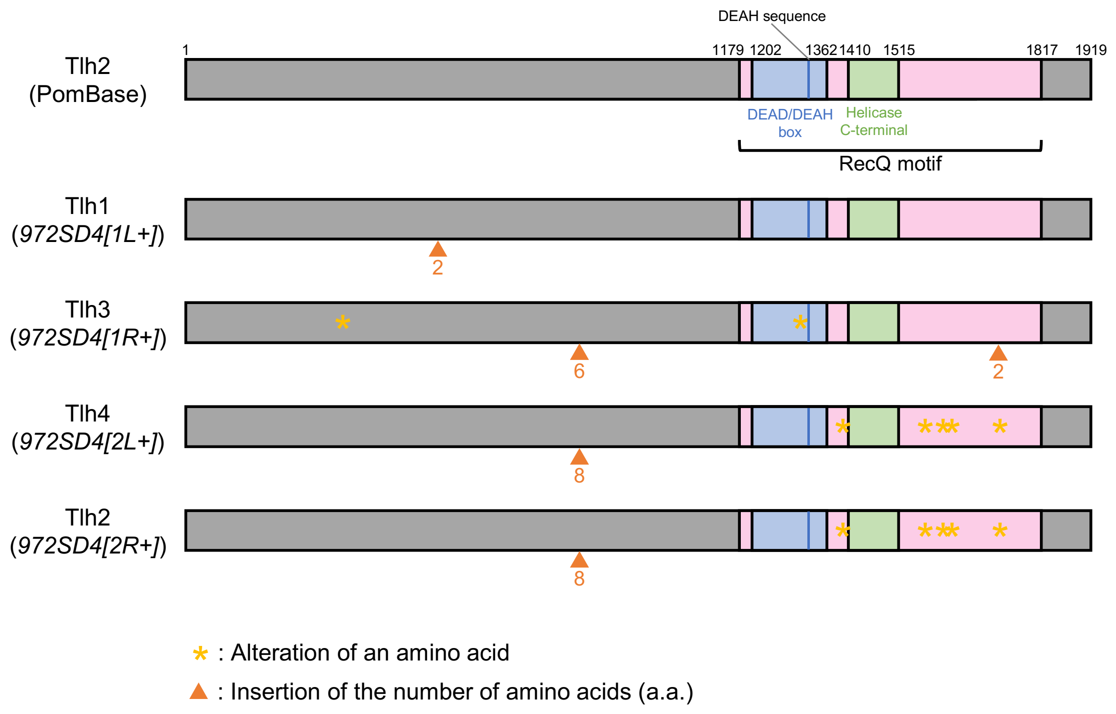

Supplementary Fig. 11 Variation of amino acid sequences among Tlh1–4 in *972SD4*.

Comparison of amino acid sequences of Tlh1–4 proteins in *972SD4* with that of Tlh2 in PomBase. Yellow asterisk, alteration of an amino acid; orange triangle, insertion of the number of amino acids. Conserved domains in RecQ-type helicases are indicated[9].

Supplementary Table 1 Definitions of homologous block and box sequences in *SH-D* regions.

Length and position of each sequence corresponding to PomBase are shown.

		Position in PomBase	
Block or box	Length (bp)	Ch	bp
I	4,629	2	4,532,900 – 4,537,528
II (*tlh*)	6,015	2	4,526,885 – 4,532,899
III	6,471	2	4,520,414 – 4,526,884
ΨSH1L-L	560	1	12,136 – 12,695
IV	3,137	1	12,696 – 15,832
ΨSH2R	550	2	4,519,864 – 4,520,413
V	5,027	2	4,514,837 – 4,519,863
ΩSH1L-L	1,279	1	21,352 – 22,630
ΩSH2R-L	1,258	2	4,513,579 – 4,514,836
VI	5,868	2	4,507,711 – 4,513,578
ΩSH2R-R	1,227	2	4,506,484 – 4,507,710
VII	6,173	2	4,500,311 – 4,506,483
VIII	1,872	2	4,498,439 – 4,500,310
IX	1,347	2	4,497,092 – 4,498,438
X	4,962	2	4,492,130 – 4,497,091
XI	8,659	1	40,622 – 49,280
ε	11,504	1	49,281 – 60,784
ζ	12,489	1	5,545,205 – 5,557,693
η	507	2	29,493 – 29,999

Supplementary Table 2 Identities between genome sequences of *S. pombe* strains and those in PomBase.

				vs. PomBase					
		Positions in PomBase		*972SD4[2R+] #1*		*972SD4[2R+] #2*		JB22 (*972*)	
	Locus	Ch	bp	Match /Total (bp)	Identity (%)	Match /Total (bp)	Identity (%)	Match /Total (bp)	Identity (%)
Subtelomere	*tlh2*	2	4,525,719 – 4,533,144	7,410/7,452	99.44	7,408/7,452	99.41		
	SH-D		4,521,826 – 4,524,375	2,549/2,550	99.96	2,549/2,550	99.96		
			4,510,792 – 4,512,641	1,850/1,850	100.00	1,850/1,850	100.00		
			4,504,812 – 4,506,754	1,943/1,943	100.00	1,943/1,943	100.00		
	SU		4,489,185 – 4,490,293	1,109/1,109	100.00	1,109/1,109	100.00	1,109/1,109	100.00
			4,484,916 – 4,486,915	2,000/2,000	100.00	2,000/2,000	100.00	2,000/2,000	100.00
			4,469,756 – 4,472,805	3,050/3,050	100.00	3,050/3,050	100.00	3,050/3,050	100.00
			4,454,962 – 4,458,161	3,200/3,200	100.00	3,200/3,200	100.00	3,200/3,200	100.00
	Boundary		4,439,149 – 4,443,731	4,583/4,583	100.00	4,583/4,583	100.00	4,583/4,583	100.00
	SA		4,431,856 – 4,435,505	3,650/3,650	100.00	3,650/3,650	100.00	3,650/3,650	100.00
Shelterin complex	*rap1*	2	3,098,366 – 3,100,576	2,211/2,211	100.00	2,211/2,211	100.00	2,211/2,211	100.00
	taz1	1	3,091,237 – 3,093,228	1,992/1,992	100.00	1,992/1,992	100.00	1,992/1,992	100.00
	pot1	1	4,129,529 – 4,131,314	1,786/1,786	100.00	1,786/1,786	100.00	1,786/1,786	100.00
Non-essential genes	*gcn5*	1	4,974,116 – 4,975,480	1,365/1,365	100.00	1,365/1,365	100.00	1,365/1,365	100.00
	clr4	2	456,575 – 458,047	1,473/1,473	100.00	1,473/1,473	100.00	1,473/1,473	100.00
	sty1	1	206,864 – 207,913	1,050/1,050	100.00	1,050/1,050	100.00	1,050/1,050	100.00
	zfs1	2	3,483,454 – 3,484,668	1,215/1,215	100.00	1,215/1,215	100.00	1,215/1,215	100.00
Essential genes	*cdc13*	2	421,123 – 422,571	1,449/1,449	100.00	1,449/1,449	100.00	1,449/1,449	100.00
	act1	2	1,476,149 – 1,477,276	1,128/1,128	100.00	1,128/1,128	100.00	1,128/1,128	100.00

				vs. PomBase					
		Positions in PomBase		JB760		JB1174		JB934	
	Locus	Ch	bp	Match /Total (bp)	Identity (%)	Match /Total (bp)	Identity (%)	Match /Total (bp)	Identity (%)
Subtelomere	*tlh2*	2	4,525,719 – 4,533,144						
	SH-D		4,521,826 – 4,524,375						
			4,510,792 – 4,512,641						
			4,504,812 – 4,506,754						
	SU		4,489,185 – 4,490,293	1,109/1,109	100.00	1,109/1,109	100.00	Homologous sequences were not found	
			4,484,916 – 4,486,915	2,000/2,000	100.00	2,000/2,000	100.00		
			4,469,756 – 4,472,805	3,050/3,050	100.00	3,049/3,050	99.97	3,024/3,058	98.89
			4,454,962 – 4,458,161	3,200/3,200	100.00	3,200/3,200	100.00	3,154/3,209	98.29
	Boundary		4,439,149 – 4,443,731	4,583/4,583	100.00	4,582/4,583	99.98	4,533/4,599	98.56
	SA		4,431,856 – 4,435,505	3,650/3,650	100.00	3,650/3,650	100.00	3,630/3,655	99.32
Shelterin complex	*rap1*	2	3,098,366 – 3,100,576	2,209/2,212	99.86	2,209/2,212	99.86	2,191/2,211	99.10
	taz1	1	3,091,237 – 3,093,228	1,991/1,992	99.95	1,992/1,992	100.00	1,991/1,992	99.95
	pot1	1	4,129,529 – 4,131,314	1,785/1,786	99.94	1,786/1,786	100.00	1,786/1,786	100.00
Non-essential genes	*gcn5*	1	4,974,116 – 4,975,480	1,365/1,365	100.00	1,365/1,365	100.00	1,365/1,365	100.00
	clr4	2	456,575 – 458,047	1,473/1,473	100.00	1,473/1,473	100.00	1,472/1,473	99.93
	sty1	1	206,864 – 207,913	1,050/1,050	100.00	1,050/1,050	100.00	1,048/1,050	99.81
	zfs1	2	3,483,454 – 3,484,668	1,215/1,215	100.00	1,215/1,215	100.00	1,215/1,215	100.00
Essential genes	*cdc13*	2	421,123 – 422,571	1,449/1,449	100.00	1,449/1,449	100.00	1,448/1,449	99.93
	act1	2	1,476,149 – 1,477,276	1,127/1,128	99.91	1,128/1,128	100.00	1,128/1,128	100.00

				vs. PomBase					
		Positions in PomBase		JB858		JB873		JB1197	
	Locus	Ch	bp	Match /Total (bp)	Identity (%)	Match /Total (bp)	Identity (%)	Match /Total (bp)	Identity (%)
Subtelomere	*tlh2*	2	4,525,719 – 4,533,144						
	SH-D		4,521,826 – 4,524,375						
			4,510,792 – 4,512,641						
			4,504,812 – 4,506,754						
	SU		4,489,185 – 4,490,293	1,098/1,109	99.01	1,098/1,109	99.01	1,098/1,109	99.01
			4,484,916 – 4,486,915	1,984/2,000	99.20	1,985/2,000	99.25	1,984/2,000	99.20
			4,469,756 – 4,472,805	3,021/3,058	98.79	3,023/3,058	98.86	3,022/3,057	98.86
			4,454,962 – 4,458,161	3,153/3,217	98.01	3,151/3,221	97.83	3,153/3,219	97.95
	Boundary		4,439,149 – 4,443,731	4,528/4,603	98.37	4,529/4,602	98.41	4,522/4,607	98.15
	SA		4,431,856 – 4,435,505	3,631/3,655	99.34	3,631/3,655	99.34	3,630/3,655	99.32
Shelterin complex	*rap1*	2	3,098,366 – 3,100,576	2,192/2,211	99.14	2,192/2,211	99.14	2,183/2,211	98.73
	taz1	1	3,091,237 – 3,093,228	1,982/1,992	99.50	1,982/1,992	99.50	1,983/1,992	99.55
	pot1	1	4,129,529 – 4,131,314	1,786/1,786	100.00	1,786/1,786	100.00	1,785/1,786	99.94
Non-essential genes	*gcn5*	1	4,974,116 – 4,975,480	1,364/1,365	99.93	1,365/1,365	100.00	1,362/1,365	99.78
	clr4	2	456,575 – 458,047	1,472/1,473	99.93	1,472/1,473	99.93	1,464/1,473	99.39
	sty1	1	206,864 – 207,913	1,049/1,050	99.90	1,048/1,050	99.81	1,049/1,050	99.90
	zfs1	2	3,483,454 – 3,484,668	1,215/1,215	100.00	1,215/1,215	100.00	1,213/1,215	99.84
Essential genes	*cdc13*	2	421,123 – 422,571	1,448/1,449	99.93	1,448/1,449	99.93	1,448/1,449	99.93
	act1	2	1,476,149 – 1,477,276	1,128/1,128	100.00	1,128/1,128	100.00	1,127/1,128	99.91

Supplementary Table 3 Fission yeast strains used in this study.

972/JK107	*h*$^{-}$
JP1225	*h*$^{-}$ *ade6-M216 leu1-32 ura4-D18 his7-366* (CHP429 from C. Hoffman)
ST3524 (*SD5*)	*h*$^{+}$ *ade6-M210 leu1-32 ura4-D18 his7-366 SH1L*::*ura4*$^{+}$ *SH1R*::*his7*$^{+}$ *SH2L*::*his7*$^{+}$ *SH2R*::*his7*$^{+}$ *SH3L*::*ura4*$^{+}$
ST3479 (*SD5*)	*h*$^{-}$ *ade6-M216 leu1-32 ura4-D18 his7-366 SH1L*::*ura4*$^{+}$ *SH1R*::*his7*$^{+}$ *SH2L*::*his7*$^{+}$ *SH2R*::*his7*$^{+}$ *SH3L*::*ura4*$^{+}$
ST5057 (*972SD4[1L+]* #1)	*h*$^{+}$ *ade6-M216 leu1-32 ura4-D18 his7-366 SH1R*::*his7*$^{+}$ *SH2L*::*his7*$^{+}$ *SH2R*::*his7*$^{+}$ *SH3L*::*ura4*$^{+}$
ST5058 (*972SD4[1L+]* #2)	*h*$^{-}$ *ade6-M216 leu1-32 ura4-D18 his7-366 SH1R*::*his7*$^{+}$ *SH2L*::*his7*$^{+}$ *SH2R*::*his7*$^{+}$ *SH3L*::*ura4*$^{+}$
ST5345 (*972SD4[1L+]* #3)	*h*$^{-}$ *ade6-M210 leu1-32 ura4-D18 SH1R*::*his7*$^{+}$ *SH2L*::*his7*$^{+}$ *SH2R*::*his7*$^{+}$ *SH3L*::*ura4*$^{+}$
ST5059 (*972SD4[1R+]* #1)	*h*$^{-}$ *ura4-D18 SH1L*::*ura4*$^{+}$ *SH2L*::*his7*$^{+}$ *SH2R*::*his7*$^{+}$ *SH3L*::*ura4*$^{+}$
ST5346 (*972SD4[1R+]* #2)	*h*$^{+}$ *ade6-M216 leu1-32 ura4-D18 his7-366 SH1L*::*ura4*$^{+}$ *SH2L*::*his7*$^{+}$ *SH2R*::*his7*$^{+}$ *SH3L*::*ura4*$^{+}$
ST5347 (*972SD4[1R+]* #3)	*h*$^{+}$ *ade6-M210 leu1-32 his7-366 SH1L*::*ura4*$^{+}$ *SH2L*::*his7*$^{+}$ *SH2R*::*his7*$^{+}$ *SH3L*::*ura4*$^{+}$
ST4932 (*972SD4[2L+]* #1)	*h*$^{-}$ *ura4-D18 SH1L*::*ura4*$^{+}$ *SH1R*::*his7*$^{+}$ *SH2R*::*his7*$^{+}$*SH3L*::*ura4*$^{+}$
ST5060 (*972SD4[2L+]* #2)	

h$^-$ *ade6-M210 ura4-D18 SH1L*::*ura4*$^+$ *SH1R*::*his7*$^+$
SH2R::*his7*$^+$*SH3L*::*ura4*$^+$

ST5348 (*972SD4[2L+]* #3)
h$^+$ *ade6-M210 leu1-32 ura4-D18 his7-366 SH1L*::*ura4*$^+$ *SH1R*::*his7*$^+$
SH2R::*his7*$^+$*SH3L*::*ura4*$^+$

ST5349 (*972SD4[2R+]* #1)
h$^-$ *ade6-M216 leu1-32 ura4-D18 his7-366 SH1L*::*ura4*$^+$ *SH1R*::*his7*$^+$
SH2L::*his7*$^+$ *SH3L*::*ura4*$^+$

ST5350 (*972SD4[2R+]* #2)
h$^+$ *ade6-M210 leu1-32 ura4-D18 his7-366 SH1L*::*ura4*$^+$ *SH1R*::*his7*$^+$
SH2L::*his7*$^+$ *SH3L*::*ura4*$^+$

ST5351 (*972SD4[2R+]* #3)
h$^-$ *ade6-M210 SH1L*::*ura4*$^+$ *SH1R*::*his7*$^+$ *SH2L*::*his7*$^+$ *SH3L*::*ura4*$^+$

For Fig. 8c

JP3827 (972 *clr4*Δ) *h*$^-$ *clr4*::*kanMX6*

ST3499 (*SD5 clr4*Δ) *h*$^-$ *ade6-M216 leu1-32 ura4-D18 his7-366 SH1L*::*ura4*$^+$ *SH1R*::*his7*$^+$
SH2L::*his7*$^+$ *SH2R*::*his7*$^+$ *SH3L*::*ura4*$^+$ *clr4*::*hphMX6*

JP5831 (*972SD4[1L+] clr4*Δ #1)
h$^+$ *ade6-M216 leu1-32 ura4-D18 his7-366 SH1R*::*his7*$^+$ *SH2L*::*his7*$^+$
SH2R::*his7*$^+$ *SH3L*::*ura4*$^+$ *clr4*::*hphMX6*

JP5833 (*972SD4[1R+] clr4*Δ #1)
h$^-$ *ura4-D18 SH1L*::*ura4*$^+$ *SH2L*::*his7*$^+$ *SH2R*::*his7*$^+$ *SH3L*::*ura4*$^+$
clr4::*hphMX6*

JP5835 (*972SD4[2L+] clr4*Δ #1)
h$^-$ *ura4-D18 SH1L*::*ura4*$^+$ *SH1R*::*his7*$^+$ *SH2R*::*his7*$^+$*SH3L*::*ura4*$^+$
clr4::*hphMX6*

JP5837 (*972SD4[2R+] clr4*Δ #1)
h$^-$ *ade6-M216 leu1-32 ura4-D18 his7-366 SH1L*::*ura4*$^+$ *SH1R*::*his7*$^+$
SH2L::*his7*$^+$ *SH3L*::*ura4*$^+$ *clr4*::*hphMX6*

Supplementary Table 4 PCR primers used in this study.

<u>For amplification of the *SPBCPT2R1.03* ORF</u>

st498	5′- ATGAGTATTGAATTCGATGACAG-3′
st499	5′- ACCATTCAAATTGTATTTCAAGTATG-3′

<u>For amplification of *SH-D* regions</u>

subtel-5.2-7.9 kb

jk618	5′-GCCTACCGCTTGCAGTTGTT-3′
jk621	5′-ACAGTAAACTATGATCGCTTTTGAAGAC-3′

subtel-7.9-9.3 kb

jk620	5′-TTCTTAATCATTATCAAGTATTCATTGCAA-3′
jk452	5′-CAACTTGCGAGAATGTAAACTACGTATTC-3′

subtel-9.1-10.8 kb

jk451	5′-TGCCTTTCTAGCCCATGACCTA-3′
jk385	5′-CAGCATTAACCAACAGTGGTCTTC-3′

subtel-10.7-13.2 kb

jk384	5′-GCTCTCGACAAAGCCGTTCT-3′
jk387	5′-CATCAGCAACGTCGCCAACT-3′

subtel-13.0-15.1 kb

jk386	5′-TTCCAAGTATGCCAGCTTATCATC-3′
jk454	5′-TAAAGATTCATGGAACCATCATTTG-3′

subtel-14.9-17.2 kb

jk453	5′-AAAGAATTCGAATCACCCATACCA-3′
jk683	5′-GGGTCAAAACCTGCGCATAA-3′

subtel-17.1-19.0 kb

jk682	5′-AATTTCAGTTGCCAAGGGACA-3′
jk456	5′-GACCGCTACGCAACCATAAAG-3′

subtel-18.9-21.3 kb

jk455	5′-AACGAGTTGTGCAATGTTAGTAAGGT-3′
jk561	5′-TGACCACCCTCATAAGCCTGT-3′

subtel-21.3-23.3 kb

jk560 5′-TGTTACGTGGCAAGGCAGACT-3′

jk559 5′-AACTCGCTTGATGAATGGGTG-3′

subtel-23.3-26.2 kb

jk558 5′-GACAACCCGACCCATGGAT-3′

jk555 5′-GACCCCAATGGCCGAGAT-3′

subtel-26.2-27.7 kb

jk554 5′-GCTGCATGTTTATATCCTTGGCT-3′

jk689 5′-GAGCCCAATGACCAAAATACAAA-3′

subtel-27.7-29.0 kb

jk688 5′-CAGCCCTGGGTGCAACTATAA-3′

jk389 5′-TGTTACTCATACTTGAAATACAATTTGAATG-3′

subtel-34.9-36.9 kb

tk74 5′-ACATTCAGAAACAACAGTTGG-3′

jk691 5′-GCTCCATCCATTGTCATTGGT-3′

For detection of lengths of *SH-D* regions

subtel2R-4.2-11.1 kb **(i)**

tk121 5′-ATTCTTTTCACTTTCTTTTCCTCG-3′

tk66 5′-CCATGATGTCGTTACGGC-3′

subtel2R-9.3-15.7 kb **(ii)**

jk451 5′-TGCCTTTCTAGCCCATGACCTA-3′

tk79 5′-CATTCATTTTCTACACGCTCTATTATTTC-3′

subtel2R-14.8-20.7 kb **(iii)**

jk1611 5′-CATCCGGCGATATCTCTAGCAG-3′

tk44 5′-CAAAGTTGTATTCATGCTTGTTAC-3′

subtel2R-20.6-22.1 kb **(iv)**

tk114 5′-TCATTACCAAAGTTTAAAACGC-3′

tk113 5′-AGTCTGAATGTGCCAACTC-3′

subtel2R-22.1-26.7 kb **(v)**

tk122 5′-GAGTTGGCACATTCAGACT-3′

tk123 5′-GGTATCGGTCACCGATATAGC-3′

subtel2R-26.7-35.4 kb **(vi)**

tk102 5′-GCTATATCGGTGACCGATACC-3′

tk130 5′-GGGAGTCCAAATTGGTTCAA-3′

***subtel2R-35.4-40.9 kb* (vii)**

tk76 5′-TTGAACCAATTTGGACTCCC-3′

tk144 5′-GGTGTTGCGGGATCTTCA-3′

***subtel2R-40.7-44.1 kb* (viii)**

jk692 5′-CGCGCACTTTTCGGACATA-3′

tk115 5′-GTATGATTTTTCAAACTGTCCC-3′

***subtel2R-44.1-47.5 kb* (ix)**

tk124 5′-GGGACAGTTTGAAAAATCATAC-3′

tk125 5′-CAACGACTAAGTCTATACATCGG-3′

***rap1* (control)**

jk900 5′-AATTGTAAAGCCTTACTCGGCCCAAAAGGATTATACACAA-AG-3′

jk1219 5′-ATGAAGAATCTGGAGTTGACGTAG-3′

For *Ch2R* sequencing

subtel2R-17.1-19.8 kb

jk682 5′-AATTTCAGTTGCCAAGGGACA-3′

tk52 5′-GGAATTGATTATTCAACTCATTGGAC-3′

subtel2R-29.0-30.9 kb

jk388 5′-GGCACAATTTCATTTCGTTTAGTTTAC-3′

jk685 5′-TGCCGGTATTTAAGATGCACAT-3′

subtel2R-34.6-36.9 kb

jk688 5′-CAGCCCTGGGTGCAACTATAA-3′

jk691 5′-GCTCCATCCATTGTCATTGGT-3′

subtel2R-50.8-52.3 kb

jk700 5′-TCCGCAAATTTTGTTAGCCAT-3′

jk703 5′-ACGGGCGGTATGGATCAAT-3′

subtel2R-54.8-57.0 kb

jk704 5′-TTCCAATCCCAGAGTCGTGTC-3′

jk707 5′-AATAGACTTGGTCTGGATGAAGCAG-3′

subtel2R-69.0-72.2 kb

jk710	5′-GCTTTTATGCAATGTCTTGACGA-3′
tk131	5′-CCTTGATACATGGAACCTCG-3′

subtel2R-83.6-86.4 kb

tk133	5′-AGTCCTTGTCCGGCAGAATCT-3′
tk132	5′-AAGCTATCTGGGAAGCCG-3′

subtel2R-98.0-102.7 kb

km38	5′-TTCAGGATTAAGGTAACGCGGT-3′
st104	5′-TTTATGCATCCAACCGTTCGA-3′

2R-106.2-110.0 kb

st214	5′-GCTTTACTCCTTTCAGGCGTATG-3′
km41	5′-CGATCGTCCACTACTTCACGTTT-3′

For sequencing of control gene loci

rap1

jk926	5′-CACTCCACTTACTCATCCAGC-3′
jk1219	5′-ATGAAGAATCTGGAGTTGACGTAG-3′

taz1

jk1918	5′-TGGTATTCTTACATTGTTCTAAGGGATTATG-3′
jk1222	5′-TAACAAAACTATCCGAGTCTTGTC-3′

pot1

jk1916	5′-CTTTTACCAATTCCATTTAGGTTTCTGAG-3′
jk1917	5′-CTTTATAAAATTCTCCCAATATAGAGCTTTCC-3′

gcn5

jk1914	5′-TGCTCGTTTCATCTTGTATCGTTCTT-3′
jk1915	5′-TTTGGAAGGCACAAAAGATATTCGTG-3′

clr4

jk1908	5′-AAATTCGTTCAGGCATCATTTGG-3′
jk1909	5′-TGCTTAGAAAATGAATGACCTTTTTCAG-3′

sty1

jk1912	5′-AGTTACTTTTCGATATAGACGAAGGAC-3′
jk1913	5′-CTAAAATTCCATGGAACCGAGATGAT-3′

zfs1

jk1906 5′-TTCTTTGTTCGTTTATAACTAATC-3′

jk1907 5′-AAGGAGGATAGGAAAGTAAGCGTCATA-3′

cdc13

jk1904 5′-CTCTTTTTTATCATTTCTGTTACCTGAACC-3′

jk1905 5′-AACTAATACACATATAAAGAGCGCTTG-3′

act1

jk1910 5′-ACCTCAGCCAGCCGTGTTATAACTTA-3′

jk1911 5′-AAAGTTCCTCATGAGATGTTAAGAGC-3′

For detection of RNA and DNA from upstream of the *tlh* ORFs

jk567 (a) 5′-TTGCTGCTGCACAAAGCTTTTTG-3′

tk79 (b) 5′-CATTCATTTTCTACACGCTCTATTATTTC-3′

jk1612 (c) 5′-TGGTCGTCGCTTCAGAAATTG-3′

tk41 5′-ATGTTTTGTGCATCTTCTTTCTCC-3′

***rap1* (control)**

jk1300 5′-TATAAAGAATTCTCTAAATGAAGTTTCACGCAAGGT-3′

jk1206 5′-TATAAACCCGGGGTATCCTCAACATTCTCTTGAATC-3′

For detection of RNA from *dh*-homologous regions in the *tlh* genes

dh-homologous region

jk458 5′-TCGATAGAGATACGTTCATCTCATAGTGT-3′

jk457 5′-CACTCATTTCTTTGCTGATCACTTACT-3′

***his1* (control)**

jk1335 5′-CGAAGACGTGCTTCAGCGA-3′

jk1336 5′-TGTCCACCTCGGAATCACTG-3′

Supplementary references

1 Ohno, Y., Ogiyama, Y., Kubota, Y., Kubo, T. & Ishii, K. Acentric chromosome ends are prone to fusion with functional chromosome ends through a homology-directed rearrangement. *Nucleic Acids Res.* **44**, 232-244 (2016).

2 Tashiro, S., Nishihara, Y., Kugou, K., Ohta, K. & Kanoh, J. Subtelomeres constitute a safeguard for gene expression and chromosome homeostasis. *Nucleic Acids Res.* **45**, 10333-10349 (2017).

3 Chen, Y. *et al.* A conserved motif within RAP1 has diversified roles in telomere protection and regulation in different organisms. *Nat. Struct. Mol. Biol.* **18**, 213-221 (2011).

4 Deng, W. *et al.* Fission yeast telomere-binding protein Taz1 is a functional but not a structural counterpart of human TRF1 and TRF2. *Cell Res.* **25**, 881-884 (2015).

5 Kanoh, J. & Ishikawa, F. spRap1 and spRif1, recruited to telomeres by Taz1, are essential for telomere function in fission yeast. *Curr. Biol.* **11**, 1624-1630 (2001).

6 Fujita, I., Tanaka, M. & Kanoh, J. Identification of the functional domains of the telomere protein Rap1 in *Schizosaccharomyces pombe*. *PLoS One* **7**, e49151 (2012).

7 Xue, J. *et al.* Structure of the fission yeast S. pombe telomeric Tpz1-Poz1-Rap1 complex. *Cell Res.* **27**, 1503-1520 (2017).

8 Hu, C. *et al.* The inner nuclear membrane protein Bqt4 in fission yeast contains a DNA-binding domain essential for telomere association with the nuclear envelope. *Structure* **27**, 335-343 (2019).

9 Mandell, J. G., Goodrich, K. J., Bahler, J. & Cech, T. R. Expression of a RecQ helicase homolog affects progression through crisis in fission yeast lacking telomerase. *J. Biol. Chem.* **280**, 5249-5257 (2005).
