# Supplementary material for: Complete sequences of Schizosaccharomyces pombe subtelomeres reveal multiple patterns of genome variation
Source: Nat Commun. 2021 Jan 27;12:611. doi: 10.1038/s41467-020-20595-1 (PMC7840980; doi:10.1038/s41467-020-20595-1)
Supplement: Supplementary file 2 — Reporting Summary [file 41467_2020_20595_MOESM2_ESM.pdf]

nature research

Corresponding author(s): Junko Kanoh

Last updated by author(s): Nov 17, 2020

Reporting Summary

Nature Research wishes to improve the reproducibility of the work that we publish. This form provides structure for consistency and transparency in reporting. For further information on Nature Research policies, see our Editorial Policies and the Editorial Policy Checklist.

Statistics

For all statistical analyses, confirm that the following items are present in the figure legend, table legend, main text, or Methods section.

n/a	Confirmed	
☐	☒	The exact sample size (n) for each experimental group/condition, given as a discrete number and unit of measurement
☐	☒	A statement on whether measurements were taken from distinct samples or whether the same sample was measured repeatedly
☒	☐	The statistical test(s) used AND whether they are one- or two-sided<br>*Only common tests should be described solely by name; describe more complex techniques in the Methods section.*
☒	☐	A description of all covariates tested
☒	☐	A description of any assumptions or corrections, such as tests of normality and adjustment for multiple comparisons
☐	☒	A full description of the statistical parameters including central tendency (e.g. means) or other basic estimates (e.g. regression coefficient) AND variation (e.g. standard deviation) or associated estimates of uncertainty (e.g. confidence intervals)
☒	☐	For null hypothesis testing, the test statistic (e.g. F, t, r) with confidence intervals, effect sizes, degrees of freedom and P value noted<br>*Give P values as exact values whenever suitable.*
☒	☐	For Bayesian analysis, information on the choice of priors and Markov chain Monte Carlo settings
☒	☐	For hierarchical and complex designs, identification of the appropriate level for tests and full reporting of outcomes
☒	☐	Estimates of effect sizes (e.g. Cohen's d, Pearson's r), indicating how they were calculated

Our web collection on statistics for biologists contains articles on many of the points above.

Software and code

Policy information about availability of computer code

Data collection	DNA Sequencing Analysis Software v5.4 (Applied Biosystems)
Data analysis	Porechop, v0.2.4; fastp, v0.20.1; Canu 2.0; MUMmer4; AliView, v1.26; MAFFT, v7.453; Minimap2, v2.17-r941; Racon, v1.4.13; Pilon, v1.23; NCBI nucleotide BLAST (blastn), v2.10.0+,

For manuscripts utilizing custom algorithms or software that are central to the research but not yet described in published literature, software must be made available to editors and reviewers. We strongly encourage code deposition in a community repository (e.g. GitHub). See the Nature Research guidelines for submitting code & software for further information.

Data

Policy information about availability of data

All manuscripts must include a data availability statement. This statement should provide the following information, where applicable:

- Accession codes, unique identifiers, or web links for publicly available datasets
- A list of figures that have associated raw data
- A description of any restrictions on data availability

DNA sequences of newly sequenced SH regions are available in the DNA Data Bank of Japan (DDBJ) under accession codes LC521649 (~1.7 kb of the SH-D region [SH1L] in 972SD4[1L+] #1), LC521650 (~1.7 kb of the SH-D region [SH1L] in 972SD4[1L+] #2), LC521651 (the SH1L region in 972SD4[1L+] #1), LC521652 (the SH1L region in 972SD4[1L+] #2), LC521653 (the SH1R region in 972SD4[1R+] #1), LC521654 (the SH1R region in 972SD4[1R+] #2), LC521655 (the SH2L region in 972SD4[2L+] #1), LC521656 (the SH2L region in 972SD4[2L+] #2), LC521657 (the SH2R region in 972SD4[2R+] #1), and LC521658 (the SH2R region in 972SD4[2R+] #2).

Figs. 3, 5a, 6a, 7, and 8b, Supplementary Figs. 3, 6, 7, 9, and 11, and Supplementary Table 2 are associated with these sequence data.

Field-specific reporting

Please select the one below that is the best fit for your research. If you are not sure, read the appropriate sections before making your selection.

☒ Life sciences ☐ Behavioural & social sciences ☐ Ecological, evolutionary & environmental sciences

For a reference copy of the document with all sections, see nature.com/documents/nr-reporting-summary-flat.pdf

Life sciences study design

All studies must disclose on these points even when the disclosure is negative.

Sample size	For the analysis of dh RNA expression, it is generally thought that three independent experiments are sufficient.
Data exclusions	No data were excluded.
Replication	dh RNA expression was analyzed for three times in two different strain background.
Randomization	Not applicable.
Blinding	Not applicable.

Reporting for specific materials, systems and methods

We require information from authors about some types of materials, experimental systems and methods used in many studies. Here, indicate whether each material, system or method listed is relevant to your study. If you are not sure if a list item applies to your research, read the appropriate section before selecting a response.

Materials & experimental systems

n/a	Involved in the study
☒	☐ Antibodies
☒	☐ Eukaryotic cell lines
☒	☐ Palaeontology and archaeology
☒	☐ Animals and other organisms
☒	☐ Human research participants
☒	☐ Clinical data
☒	☐ Dual use research of concern

Methods

n/a	Involved in the study
☒	☐ ChIP-seq
☒	☐ Flow cytometry
☒	☐ MRI-based neuroimaging
